# Correction: The *Bothriolepis* (Placodermi, Antiarcha) material from the Valentia Slate Formation of the Iveragh Peninsula (middle Givetian, Ireland): Morphology, evolutionary and systematic considerations, phylogenetic and palaeogeographic implications

**DOI:** 10.1371/journal.pone.0320508

**Published:** 2025-06-16

**Authors:** Vincent Dupret, Hannah M. Byrne, Nélia Castro, Øyvind Hammer, Kenneth T. Higgs, John A. Long, Grzegorz Niedźwiedzki, Martin Qvarnström, Iwan Stössel, Per E. Ahlberg

The sixth author’s name is spelled incorrectly. The correct name is: John A. Long. The correct citation is: Dupret V, Byrne HM, Castro N, Hammer Ø, Higgs KT, Long JA, et al. (2023) The *Bothriolepis* (Placodermi, Antiarcha) material from the Valentia Slate Formation of the Iveragh Peninsula (middle Givetian, Ireland): Morphology, evolutionary and systematic considerations, phylogenetic and palaeogeographic implications. PLoS ONE 18(2): e0280208. https://doi.org/10.1371/journal.pone.0280208.

In the Non-Bothriolepis genera subsection of A brief history of systematics and classification under Discussion, there is an error in the sixth sentence of the first paragraph. The correct sentence is: Diagnoses of the following taxa may be copied from original publications without substantial modification deemed possible.

In the Notes on the stratigraphic uncertainties of some taxa subsection of the Discussion, there is an error in the last bulleted paragraph. The correct bulleted paragraph is: Lastly, B. alvesiensis occurs in the Alves beds considered as mid–late Frasnian according to Becker et al. [107] and Parfitt et al. [132]. Rosebrae beds are considered as late Famennian according to Marshall in Rogers [133] (see Stephenson and Gould, 1995 [134], at http://earthwise.bgs.ac.uk/index.php/Devonian,_Grampian_Highlands); Rosebrae beds are considered as early Famennian by Sallan and Coates [96].

In the Fig 15 and S1 File, there is an error in the localities of specimens. The authors have provided the correct version of Fig 15 and its caption and S1 File here.

**Fig 15:**
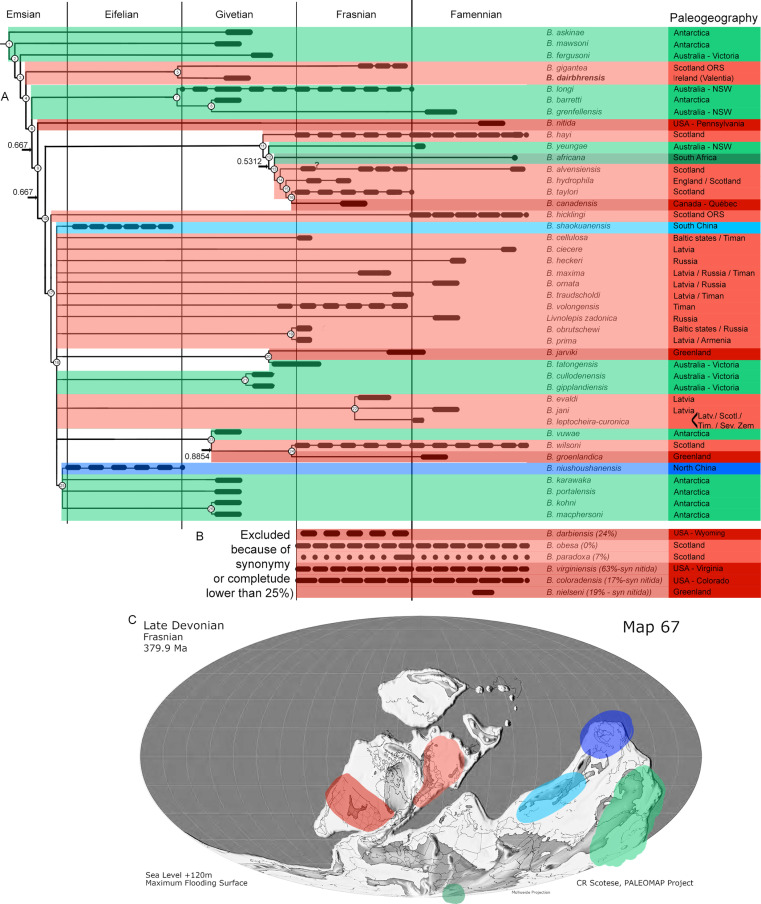
Phylogenetic analysis of the best known species of the genus *Bothriolepis* [8], with special emphasis on their palaeogeographic distribution. A. Stratigraphy correlated majority rule (50%) consensus tree (n = 96; L_50%_ = 251; CI_50%_ = 0.279; RI_50%_ = 0.365; indices for polytomies in the strict consensus tree are given on the branches; uncertain ranges indicated with longer dashes). B. Stratigraphic range and palaeogeographic distribution of taxa not included of phylogenetic analysis because of incompleteness or synonymy. C. Average distribution of *Bothriolepis* species of the phylogenetic analysis plot on a late Devonian palaeomap (palaeomap reprinted and modified from [38] under a CC BY license, with permission from Christopher Scotese, original copyright 2014). Colours in cladogram and palaeomap correspond to the following palaeogeographic zones: Euramerica in red (western part darker than eastern, boundary approximated at Caledonian Mountains), Gondwana in green (western part darker than eastern), Chinese palaeoblocks in blue (darker for Northern, lighter for Southern). For period-wise distribution of the complete Bothriolepididae, please refer to S1 Text. ORS: Old Red Sandstone; NSW: New South Wales.

The Bothriolepididae taxa, strata and localities section of the S1 file contains incorrect information. Please view the correct S1 file below.

## Supporting information

S1 File Corrections for some strata and localities of some Bothriolepididae taxa. (DOCX)

## References

[1] Dupret V, Byrne HM, Castro N, Hammer Ø, Higgs KT, Long JA, et al. The *Bothriolepis* (Placodermi, Antiarcha) material from the Valentia Slate Formation of the Iveragh Peninsula (middle Givetian, Ireland): Morphology, evolutionary and systematic considerations, phylogenetic and palaeogeographic implications. PLoS One. 2023;18(2):e0280208. doi: 10.1371/journal.pone.0280208 36821588 PMC9949654

