# Supplementary material for: The Bothriolepis (Placodermi, Antiarcha) material from the Valentia Slate Formation of the Iveragh Peninsula (middle Givetian, Ireland): Morphology, evolutionary and systematic considerations, phylogenetic and palaeogeographic implications
Source: PLoS One. 2023 Feb 23;18(2):e0280208. doi: 10.1371/journal.pone.0280208 (PMC9949654; doi:10.1371/journal.pone.0280208)
Supplement: S1 Text — (DOCX) [file pone.0280208.s001.docx]

Supplementary material (CT-data, segmentation files, STLs, data matrices, phylogenetic analyses files, 3D pdfs) can be viewed and downloaded at the following URL:

https://snd.gu.se/en/catalogue/study/preview/01477b73-c184-4f67-8e62-cb5f97a42c30#dataset
