# Supplementary material for: The Bothriolepis (Placodermi, Antiarcha) material from the Valentia Slate Formation of the Iveragh Peninsula (middle Givetian, Ireland): Morphology, evolutionary and systematic considerations, phylogenetic and palaeogeographic implications
Source: PLoS One. 2023 Feb 23;18(2):e0280208. doi: 10.1371/journal.pone.0280208 (PMC9949654; doi:10.1371/journal.pone.0280208)
Supplement: S1 File — (DOCX) [file pone.0280208.s002.docx]

SUPPLEMENTARY INFORMATION

Contents

[Elemental Analysis 3](#_Toc121943777)

[Taxon *Bothriolepis* 8](#_Toc121943778)

[Evolution of number of *Bothriolepis* species 8](#_Toc121943779)

[List of *Bothriolepis* species 8](#_Toc121943780)

[Bothriolepididae and *Bothriolepis* (Placodermi, Antiarcha) through the Devonian world 14](#_Toc121943781)

[Palaeogeographic distribution of Bothriolepididae 14](#_Toc121943782)

[Emsian 14](#_Toc121943783)

[Eifelian 15](#_Toc121943784)

[Givetian 16](#_Toc121943785)

[Frasnian 17](#_Toc121943786)

[Famennian 18](#_Toc121943787)

[Late Famennian 19](#_Toc121943788)

[Bothriolepididae taxa, strata and localities 20](#_Toc121943789)

[Data matrix taxa * characters 32](#_Toc121943790)

[Data matrix 32](#_Toc121943791)

[List of characters 32](#_Toc121943792)

[Original complete set 32](#_Toc121943793)

[Final reduced set 41](#_Toc121943794)

[Phylogenetic analyses 49](#_Toc121943795)

[Different outgroups 49](#_Toc121943796)

[all characters and all taxa (but Dianolepis) 50](#_Toc121943797)

[all taxa (but Dianolepis) 51](#_Toc121943798)

[Outgroup = Remigolepis + Grossilepis 52](#_Toc121943799)

[Outgroup = Grossilepis 53](#_Toc121943800)

[Outgroup = B. niushoushanensis 54](#_Toc121943801)

[Outgroup = B. shaokuanensis 55](#_Toc121943802)

[Outgroup = B. askinae 56](#_Toc121943803)

[Outgroup = B. askinae (B. virginiensis removed) 58](#_Toc121943804)

[Outgroup = Remigolepis + Grossilepis + Dianolepis 60](#_Toc121943805)

[Outgroup = Grossilepis + Dianolepis 62](#_Toc121943806)

[Outgroup = Dianolepis + Grossilepis 62](#_Toc121943807)

[Outgroup = Dianolepis 63](#_Toc121943808)

[Successive weighting of character “shape of preorbital recess” 64](#_Toc121943809)

[indices 64](#_Toc121943810)

[Topologies and distribution of states of character #1 65](#_Toc121943811)

[Robustness of the tree: Bremer decay index 73](#_Toc121943812)

[3D pdf captions 77](#_Toc121943813)

[NMING:F35203-UU201ab_HQ 77](#_Toc121943814)

[NMING:F35229-CH003 77](#_Toc121943815)

[NMING:F35216-UUSFB001 77](#_Toc121943816)

[NHM P 59677 (complete, ORG end RELOC files) 77](#_Toc121943817)

[NHM P 59678 77](#_Toc121943818)

[NHM P 59679 77](#_Toc121943819)

[NHM P 59687 77](#_Toc121943820)

[References 79](#_Toc121943821)

# Elemental Analysis


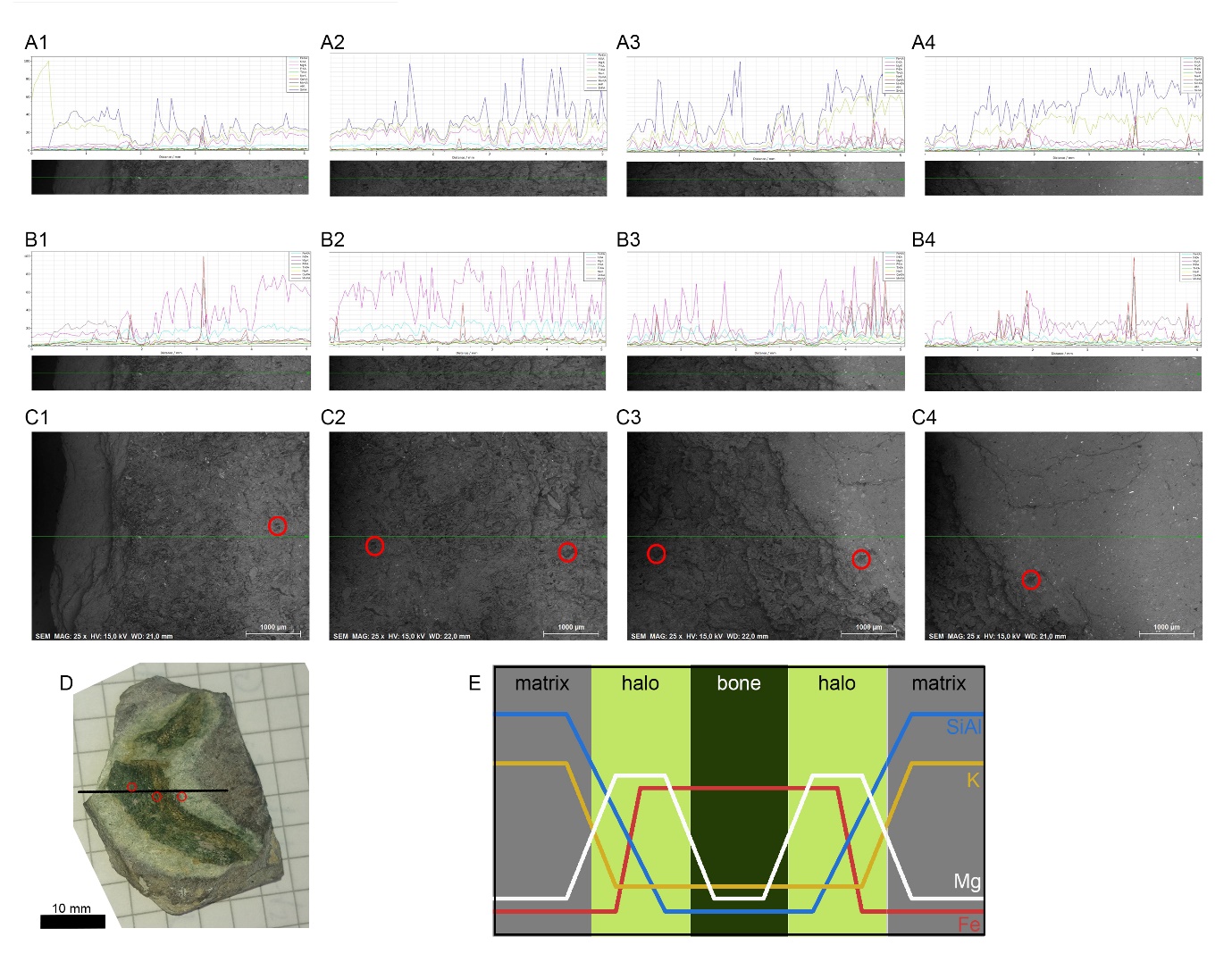


S1 Fig. A. Elemental analysis along transect in C (green line), taking into account Silicon and Aluminium. B. Elemental analysis along transect in C, without taking into account Silicon and Aluminium. C. larger images around transect (green line). D. Specimen NMING:F35231 (Vertebrata indet.) with transect (black line) and markers (red circles). E. Simplification of element distribution along transect and through matrix, halo and bone, for silicon (Si, blue), aluminium (Al, blue), potassium (K, yellow), magnesium (Mg, white), and iron (Fe, red). Below are magnification of values in transect in A1-4 and B1-4.

A1


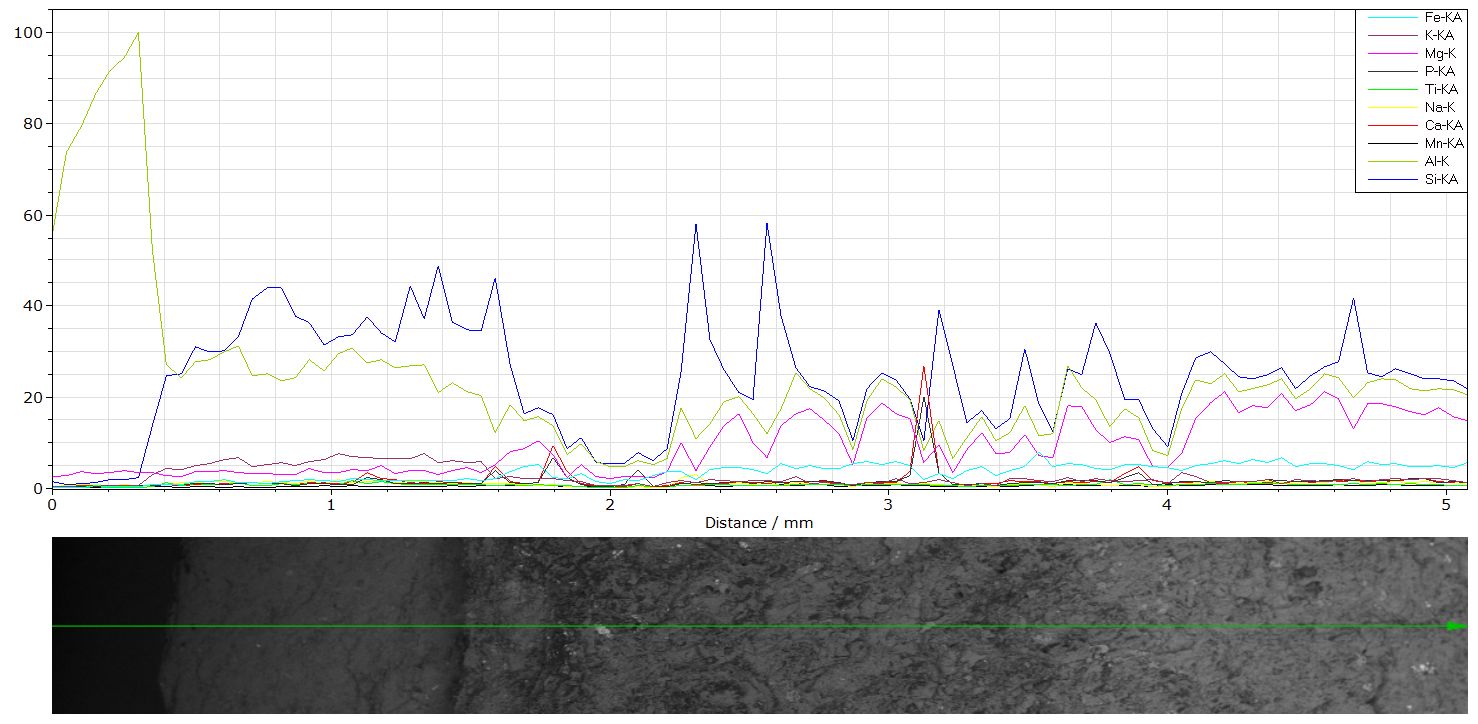


A2


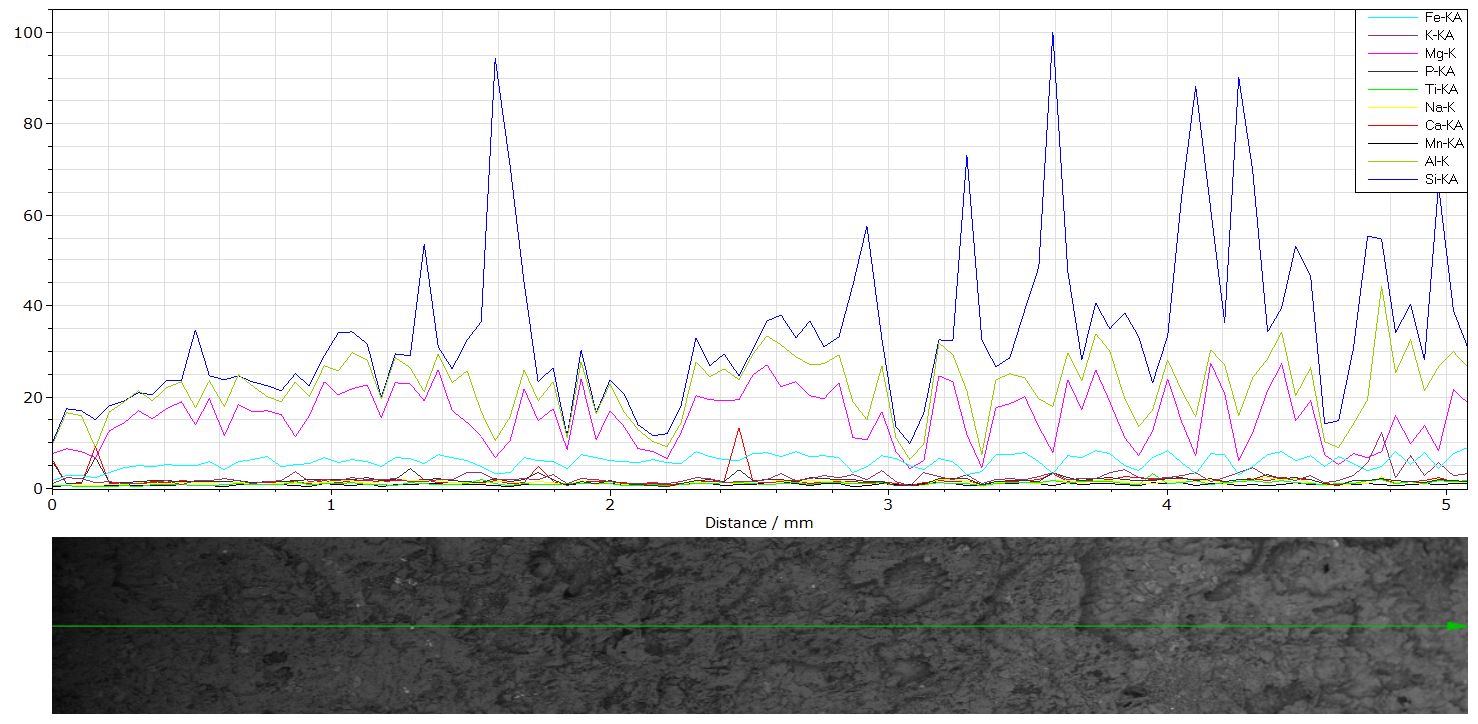


A3


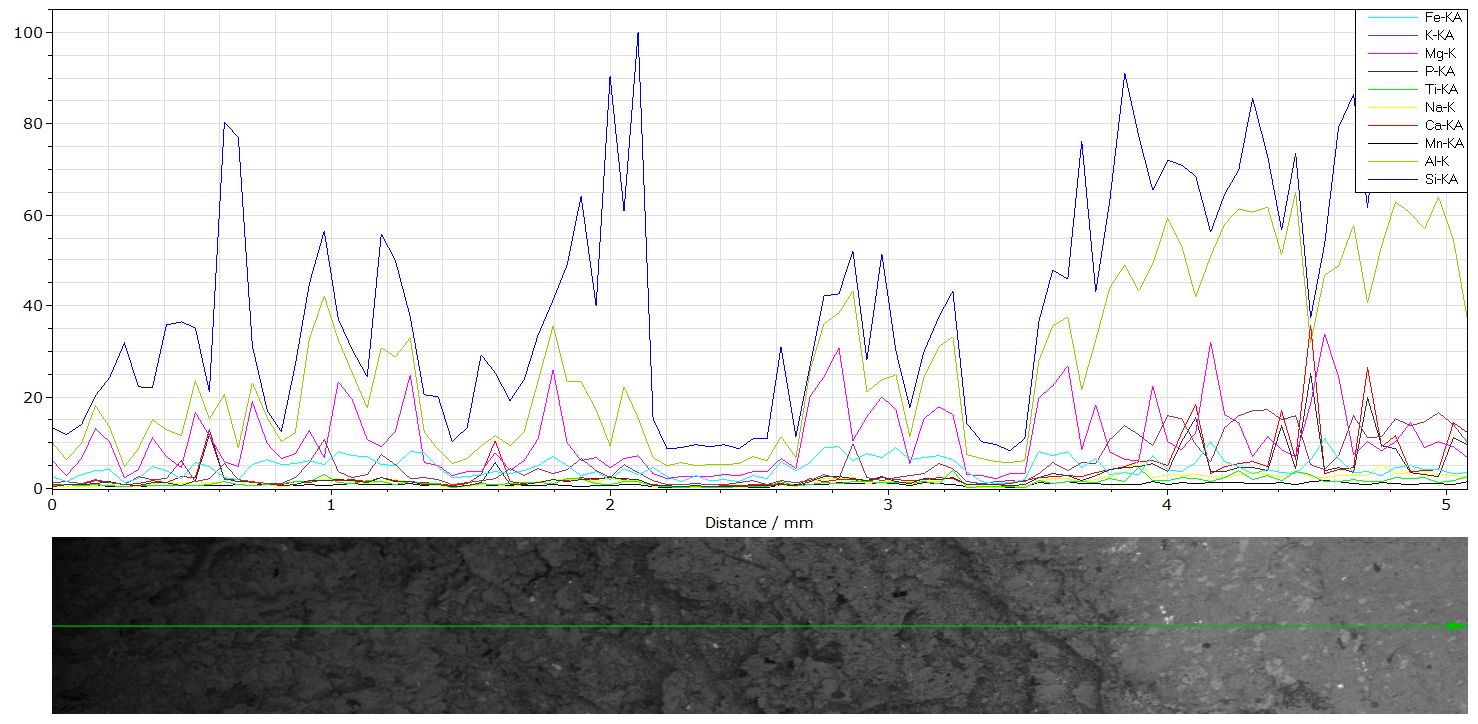


A4


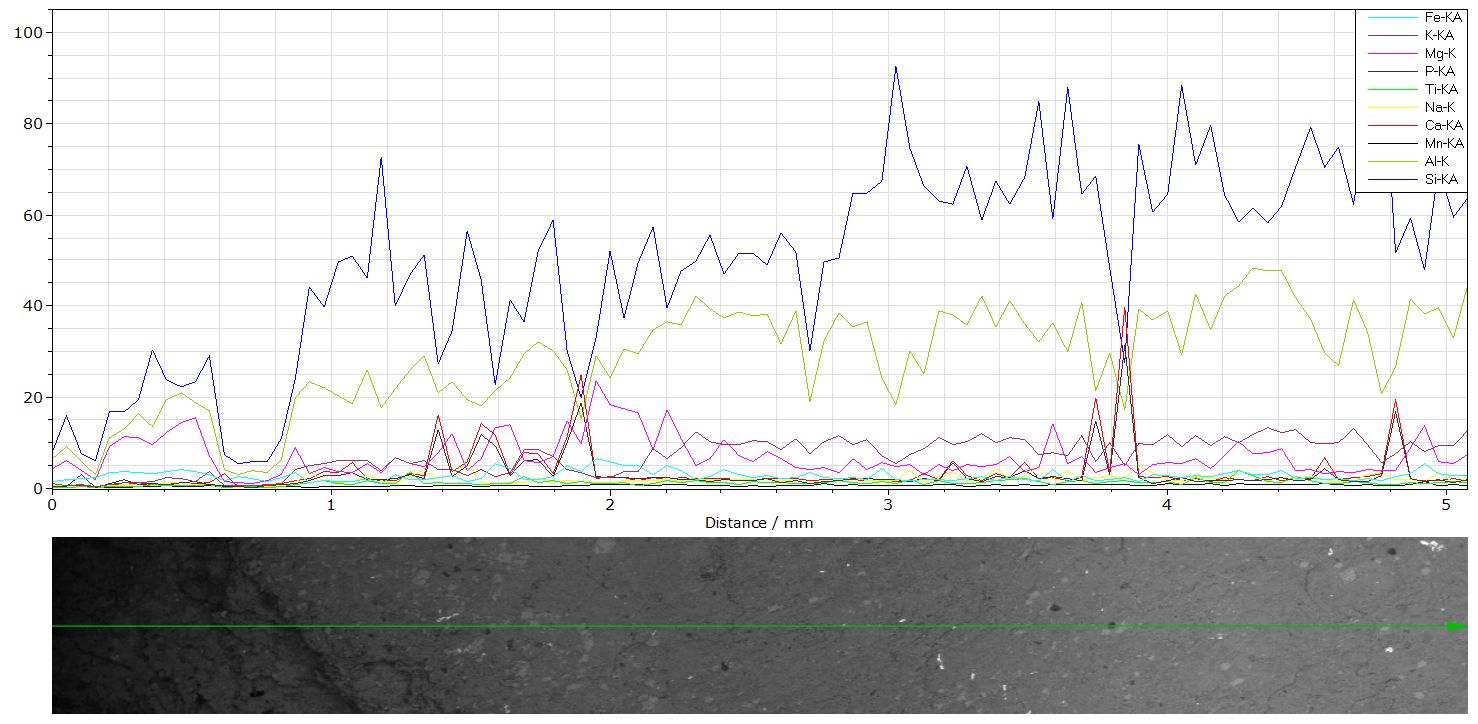


B1


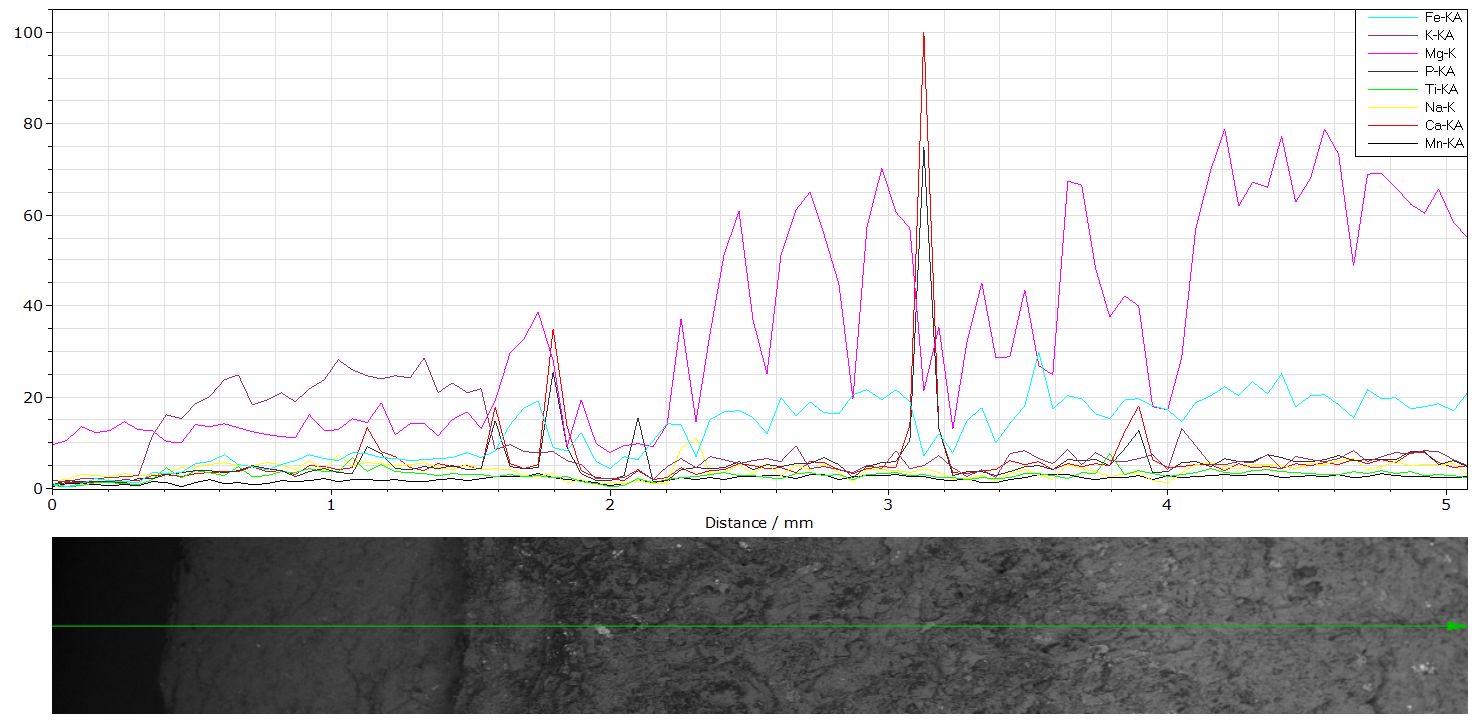


B2


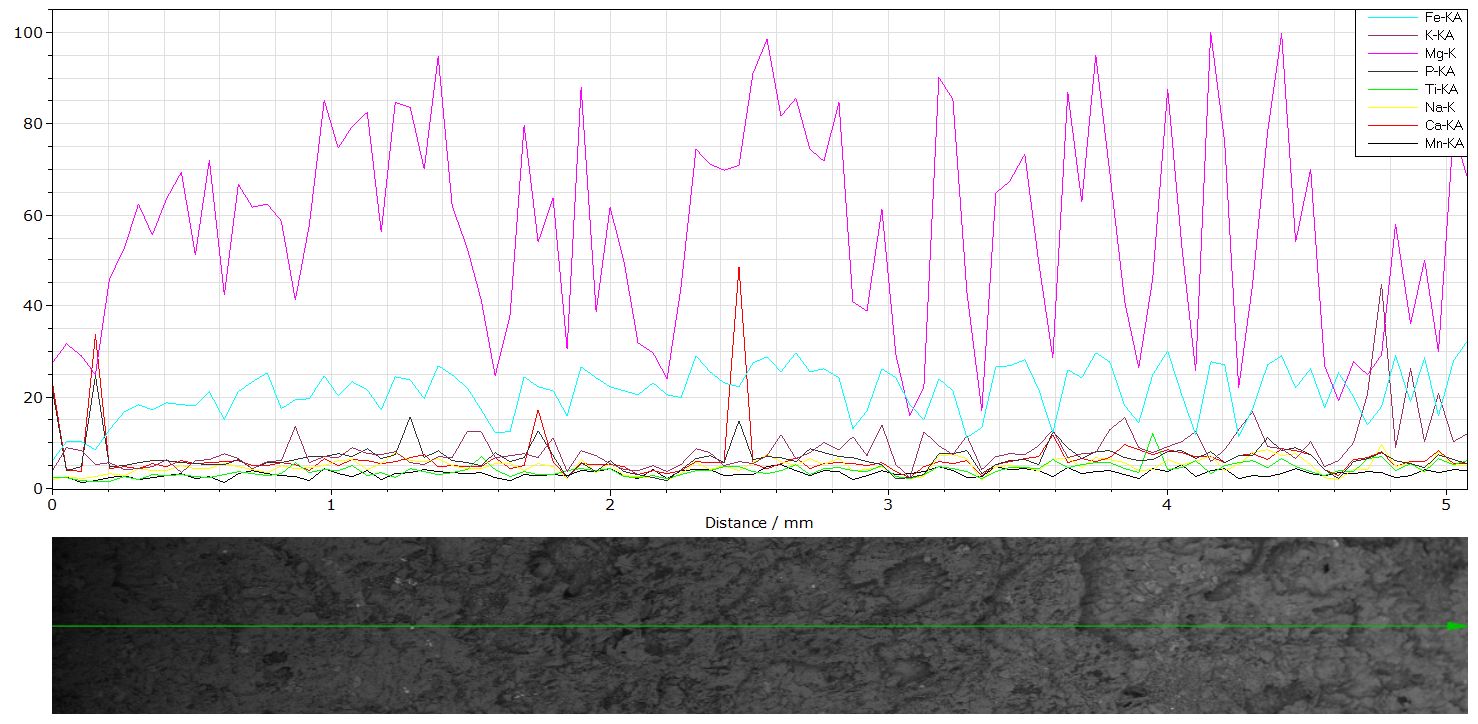


B3


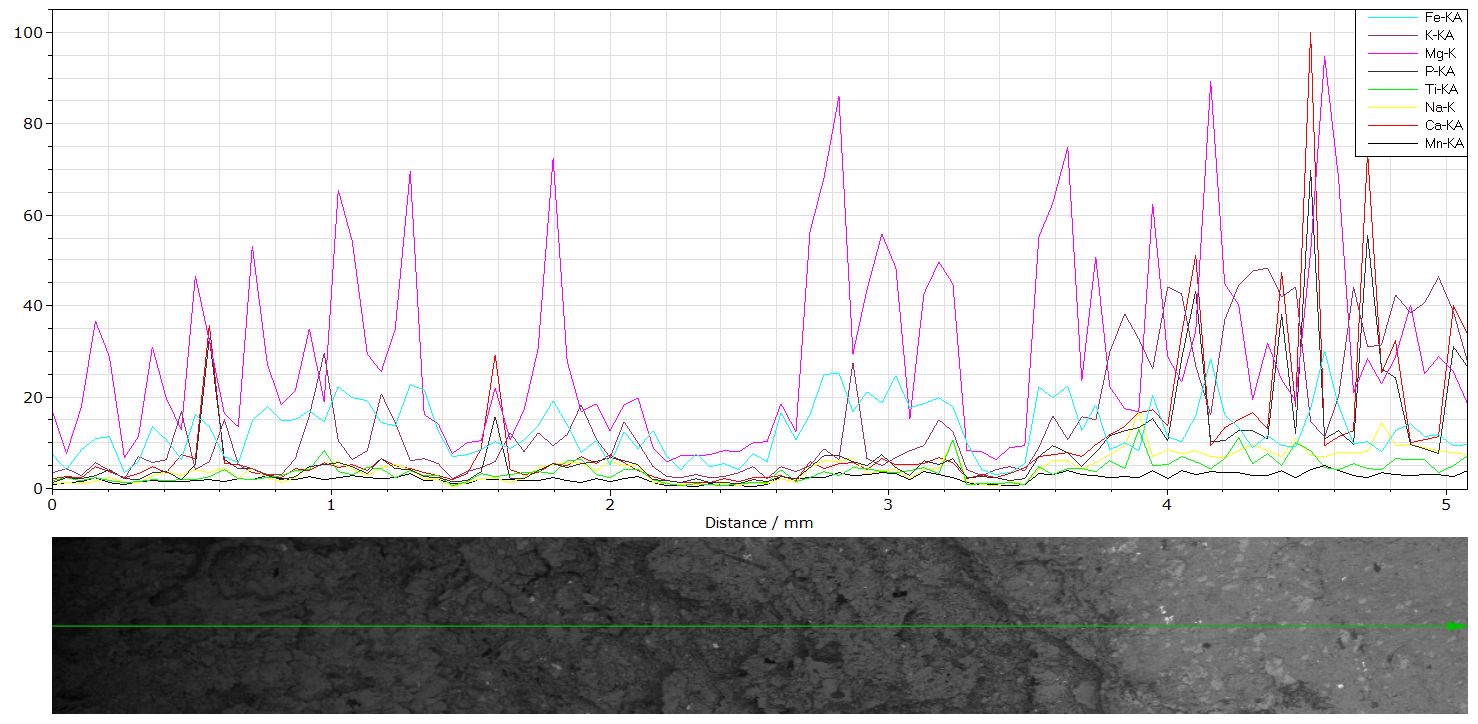


B4


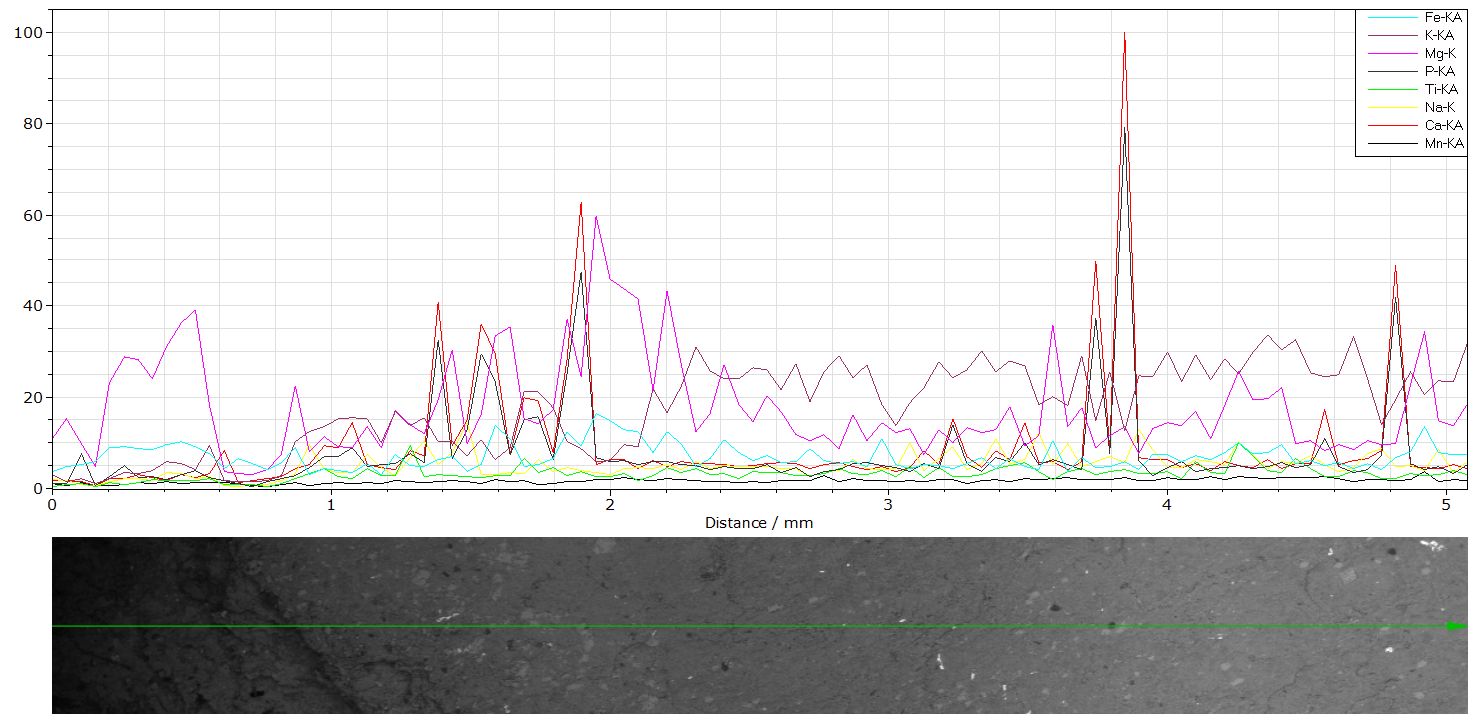


# Taxon *Bothriolepis*

## Evolution of number of *Bothriolepis* species


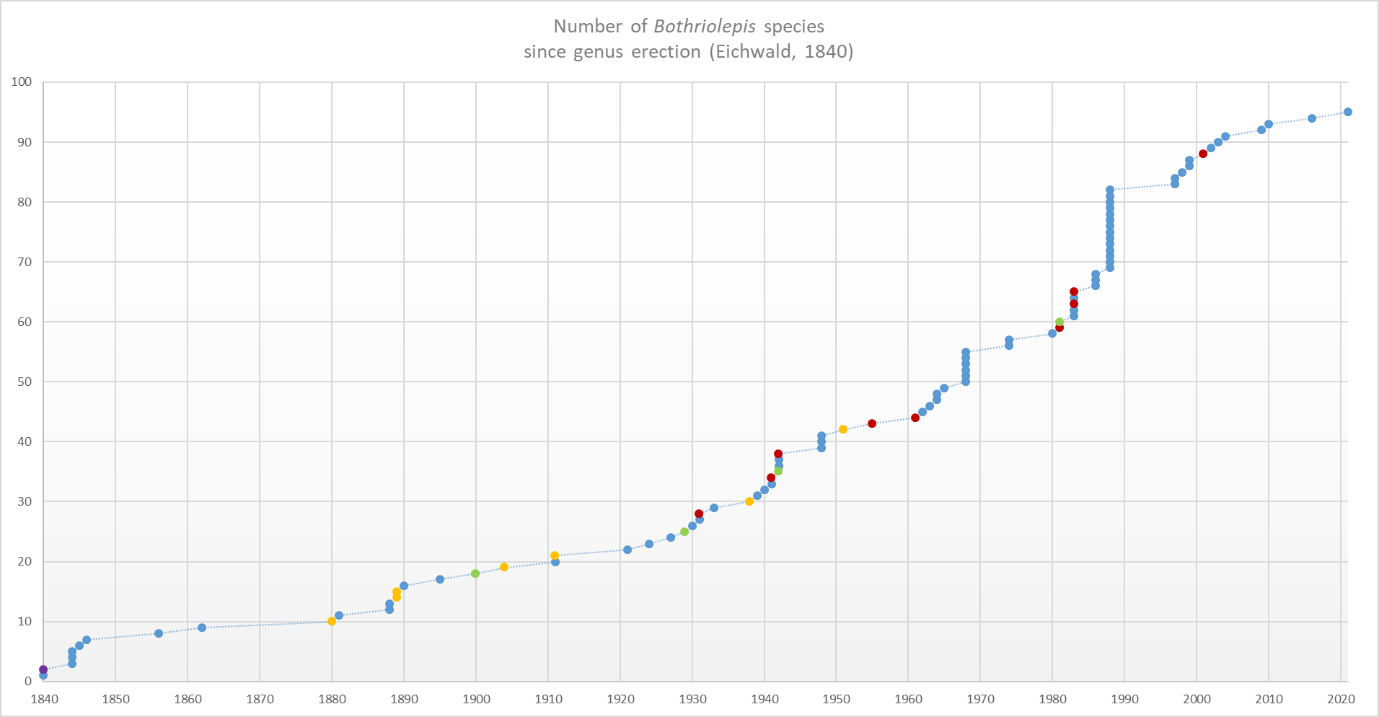


S2 Fig. Number of *Bothriolepis* species through time (blue, normal; purple, *nodem nudum*; red, change of genus; orange, synonym of other *Bothriolepis* species; green, change but back to *Bothriolepis* or new status within *Bothriolepis*).

## List of *Bothriolepis* species

| **Species** | | Taxonomic authority | **Reference of taxonomic authority** | **n** | **Comments** |
| --- | --- | --- | --- | --- | --- |
| *Bothriolepis ornata* | | Eichwald, 1840 | [1] | 1 | Type-species |
| *Botryolepis prisca Eichwald, 1840* | | Eichwald, 1840 | [1] | 2 | *nomen nudum* in Denison, 1978 [2] |
| *Bothriolepis favosa* | | Agassiz, 1844 | [3] | 3 |  |
| *Bothriolepis hydrophila* | | (Agassiz, 1844) | ([3]) | 4 |  |
| *Bothriolepis major* | | (Agassiz, 1844) | ([3]) | 5 |  |
| *Bothriolepis paradoxa* | | (Agassiz, 1845) | ([4]) | 6 |  |
| *Bothriolepis cellulosa* | | (Pander, 1846) in Keyserling, 1846 | ([5]) in [6] | 7 | Pander, 1846 always cited in Keyserling, 1846 Keyserling, 1846 |
| *Bothriolepis nitida* | | Leidy, 1856 | [7] | 8 |  |
| *Bothriolepis macrocephala* | | (Egerton, 1862) | ([8]) | 9 |  |
| *Bothriolepis panderi* | | Lahusen, 1880 | [9] | 10 | synonym of *B. favosa*, in Denison, 1978 [2] |
| *Bothriolepis canadensis* | | (Whiteaves, 1881) | ([10]) | 11 |  |
| *Bothriolepis gigantea* | | Traquair, 1888 | [11] | 12 |  |
| *Bothriolepis obesa* | | Traquair, 1888 | [11] | 13 |  |
| *Bothriolepis minor* | | Newberry, 1889 | [12] | 14 | synonym of *B. nitida* by Thomson and Thomas, 2001 [13] |
| *Bothriolepis leidyi* | | Newberry, 1889 | [12] | 15 | synonym of *B. nitida* by Thomson and Thomas, 2001 [13] |
| *Bothriolepis leptocheira* | | Traquair, 1890-92 | [14] | 16 |  |
| *Bothriolepis cristata* | | Traquair, 1895 | [15] | 17 |  |
| *Bothriolepis jeremijevi* | | Rohon, 1900 | [16] | 18 | Reassigned to *B. leptocheira jeremijevi* by Lukševičs et al., 2017 [17] |
| *Bothriolepis coloradensis* | | Eastman, 1904 | [18] | 19 | synonym of *B. nitida* by Thomson and Thomas, 2001 [13] |
| *Bothriolepis maeandrina* | | Hoffman, 1911 | [19] | 20 |  |
| *Bothriolepis retinata* | | Hoffman, 1911 | [19] | 21 | synonym of *B. cellulosa* in Denison, 1978 [2] |
| *Bothriolepis antarctica* | | Woodward, 1921 | [20] | 22 |  |
| *Bothriolepis traquairi* | | Bryant, 1924 | [21] | 23 |  |
| *Bothriolepis traudscholdi* | | Jaekel, 1927 | [22] | 24 |  |
| *Bothriolepis gippslandiensis* | | Hills, 1929 | [23] | 25 | reassigned at some point to *Hillsaspis* (by Stensiö, 1969 [24]), then reassigned to *Bothriolepis* (Young and Gorter, 1981 [25]) |
| *Bothriolepis groenlandica* | | Heintz, 1930 | Heintz, 1930 | 26 |  |
| *Bothriolepis lohesti* | | Leriche, 1931 | [26] | 27 |  |
| *Bothriolepis ymeri* | | Stensiö, 1931 | [27] | 28 | reassigned to *Remigolepis* by Denison, 1978 [2] |
| *Bothriolepis maxima* | | Gross, 1933 | [28] | 29 |  |
| *Bothriolepis stensioi* | | Sohn, 1938 | [29] | 30 | synonym of *B. canadensis* by Robertson, 1938 [30] |
| *Bothriolepis turanica* | | Obruchev, 1939 | [31] | 31 |  |
| *Bothriolepis sinensis* | | Chi, 1940 | [32] | 32 |  |
| *Bothriolepis siberica* | | Obruchev, 1941 | [33] | 33 |  |
| *Bothriolepis tuberculata* | | Gross, 1941 | [34] | 34 | reassigned to *Grossilepis* by Stensiö, 1948 [35] |
| *Bothriolepis curonica* | | Gross, 1942 | [36] | 35 | reassigned subspecies status by Lukševičs, 2001 [37] |
| *Bothriolepis obrutschewi* | | Gross, 1942 | [36] | 36 |  |
| *Bothriolepis prima* | | Gross, 1942 | [36] | 37 |  |
| *Bothriolepis spinosa* | | Gross, 1942 | [36] | 38 | reassigned to *Grossilepis* by Stensiö, 1948 [35] |
| *Bothriolepis alvesiensis* | | Stensiö, 1948 | [35] | 39 |  |
| *Bothriolepis jarviki* | | Stensiö, 1948 | [35] | 40 |  |
| *Bothriolepis nielseni* | | Stensiö, 1948 | [35] | 41 |  |
| *Bothriolepis darbiensis* | | Denison, 1951 | [38] | 42 | synonym of *B. nitida* by Thomson and Thomas, 2001 [13] |
| *Bothriolepis asiatica* | | Obrucheva, 1955 | [39] | 43 | reassigned to *Stegolepis tuberculata* by Malinovskaya, 1988 [40] (see Denison, 1978 [2]) |
| *Bothriolepis extensa* | | Sergienko, 1961 | [41] | 44 | reassigned to *Tubalepis* by Panteleyev and Moloshnikov, 2003 [42] |
| *Bothriolepis yunnanensis* | | Liu, 1962 | [43] | 45 |  |
| *Bothriolepis shaokuanensis* | | Chang, 1963 | [44] | 47 |  |
| *Bothriolepis kwangtungensis* | | P'an, 1964 | [45] | 48 |  |
| *Bothriolepis lochangensis* | | P'an, 1964 | [45] | 49 |  |
| *Bothriolepis tungseni* | | Chang, 1965 | [46] | 50 |  |
| *Bothriolepis hayi* | | Miles, 1968 | [47] | 51 |  |
| *Bothriolepis hicklingi* | | Miles, 1968 | [47] | 52 |  |
| *Bothriolepis laverocklochensis* | | Miles, 1968 | [47] | 53 |  |
| *Bothriolepis stevensoni* | | Miles, 1968 | [47] | 54 |  |
| *Bothriolepis taylori* | | Miles, 1968 | [47] | 55 |  |
| *Bothriolepis wilsoni* | | Miles, 1968 | [47] | 56 |  |
| *Bothriolepis ciecere* | | Lyarskaya, 1974 in Lyarskaja and Savvaitova, 1974 | Lyarskaya, 1974 in [48] | 57 |  |
| *Bothriolepis pavariensis* | | Lyarskaya, 1974 in Lyarskaja and Savvaitova, 1974 | Lyarskaya, 1974 in [48] | 58 |  |
| *Bothriolepis niushoushanensis* | | Pan et al., 1980 | [49] | 59 |  |
| *Bothriolepis verrucosa* | | Young and Gorter, 1981 | [25] |  | reassigned to genus *Monarolepis* by Young, 1988 [50] |
| *Bothriolepis virginiensis* | | Weems et al., 1981 | [51] | 60 | synonym of *B. nitida* by Thomson and Thomas, 2001 [13]; reconsidered valid by Weems, 2004 [52] |
| *Bothriolepis cullodenensis* | | Long, 1983 | [53] | 61 |  |
| *Bothriolepis fergusoni* | | Long, 1983 | [53] | 62 |  |
| *Bothriolepis warreni* | | Long, 1983 | [53] | 63 | reassigned to *Briagalepis* by Long et al., 1990 [54] |
| *Bothriolepis bindareei* | | Long, 1983 | [53] | 64 |  |
| *Bothriolepis zadonica* | | Obrucheva, 1983 | [55] | 65 | reassigned to *Livnolepis* by Moloshnikov, 2008 [56] |
| *Bothriolepis evaldi* | | Lyarskaja, 1986 | [57] | 66 |  |
| *Bothriolepis jani* | | Lukševičs, 1986 | [58] | 67 |  |
| *Bothriolepis tatongensis* | | Long and Werdelin, 1986 | [59] | 68 |  |
| *Bothriolepis alexi* | | Young, 1988 | [50] | 69 |  |
| *Bothriolepis askinae* | | Young, 1988 | [50] | 70 |  |
| *Bothriolepis barretti* | | Young, 1988 | [50] | 71 |  |
| *Bothriolepis karawaka* | | Young, 1988 | [50] | 72 |  |
| *Bothriolepis kohni* | | Young, 1988 | [50] | 73 |  |
| *Bothriolepis macphersoni* | | Young, 1988 | [50] | 74 |  |
| *Bothriolepis mawsoni* | | Young, 1988 | [50] | 75 |  |
| *Bothriolepis nikitinae* | | Young, 1988 | [50] | 76 |  |
| *Bothriolepis portalensis* | | Young, 1988 | [50] | 77 |  |
| *Bothriolepis vuwae* | | Young, 1988 | [50] | 78 |  |
| *Bothriolepis amankonyrica* | | Malinovskaya, 1988 | [40] | 79 |  |
| *Bothriolepis babichevi* | | Malinovskaya, 1988 | [40] | 80 | reassigned to *Tubalepis* by Panteleyev and Moloshnikov, 2003 [42] |
| *Bothriolepis kassini* | | Malinovskaya, 1988 | [40] | 81 |  |
| *Bothriolepis tastenica* | | Malinovskaya, 1988 | [40] | 82 |  |
| *Bothriolepis africana* | | Long et al., 1997 | [60] | 83 |  |
| *Bothriolepis grenfellensis* | | Johanson, 1997 | [61] | 84 |  |
| *Bothriolepis yeungae* | | Johanson, 1998 | [62] | 85 |  |
| *Bothriolepis longi* | | Johanson and Young, 1999 | [63] | 86 |  |
| *Bothriolepis volongensis* | | Lyarskaja & Lukševičs, 1999, in Lukševičs and Sorokin, 1999 | Lyarskaja & Lukševičs, 1999, in [64] | 87 | This species has an authority different from the article in which it is created (i.e. there is no publication Lyarskaja & Lukševičs, 1999 Lyarskaja & Lukševičs, 1999). |
| *Bothriolepis heckeri* | | Lukševičs, 2001 | [37] | 88 | reassigned to *Livnolepis* by Moloshnikov and Linkevich, 2020[65] |
| *Bothriolepis perija* | | Young and Moody, 2002 | [66] | 89 |  |
| *Bothriolepis sosnensis* | | Moloshnikov, 2003 | [67] | 90 |  |
| *Bothriolepis jazwicensis* | | Szrek, 2004 | [68] | 91 |  |
| *Bothriolepis dorakarasugensis* | | Moloshnikov, 2009 | [69] | 92 |  |
| *Bothriolepis sanzarensis* | | Moloshnikov, 2010b | [70] | 93 |  |
| *Bothriolepis rex* | | Downs et al., 2016 | [71] | 94 |  |
| *Bothriolepis dairbhrensis* | | current article | current article | 96 |  |
|  | |  |  |  |  |
|  | purple: nomen nudum | | | | |
|  | red: genus reassignment | | | | |
|  | orange: synonym of another *Bothriolepis* species | | | | |
|  | green: new status with *Bothriolepis* or change of genus then back to Bothriolepis | | | | |

Table A. List of *Bothriolepis* species

# Bothriolepididae and *Bothriolepis* (Placodermi, Antiarcha) through the Devonian world

## Palaeogeographic distribution of Bothriolepididae

Palaeomaps are taken from [72]. White circles indicate *Bothriolepis* species; black circles indicate other genera than *Bothriolepis* within Bothriolepididae. Chinese taxa are indicated with blue discs; Gondwanan taxa with green discs, Kazakh taxa with orange discs, and Euramerican taxa with red discs; for Gondwana and Euramerica, a darker shade indicates a western location in the supercontinent margin (colour consistent with cladograms and phylogenetic trees of the article). References for locations and stratigraphy are given after maps.

### Emsian

Emsian


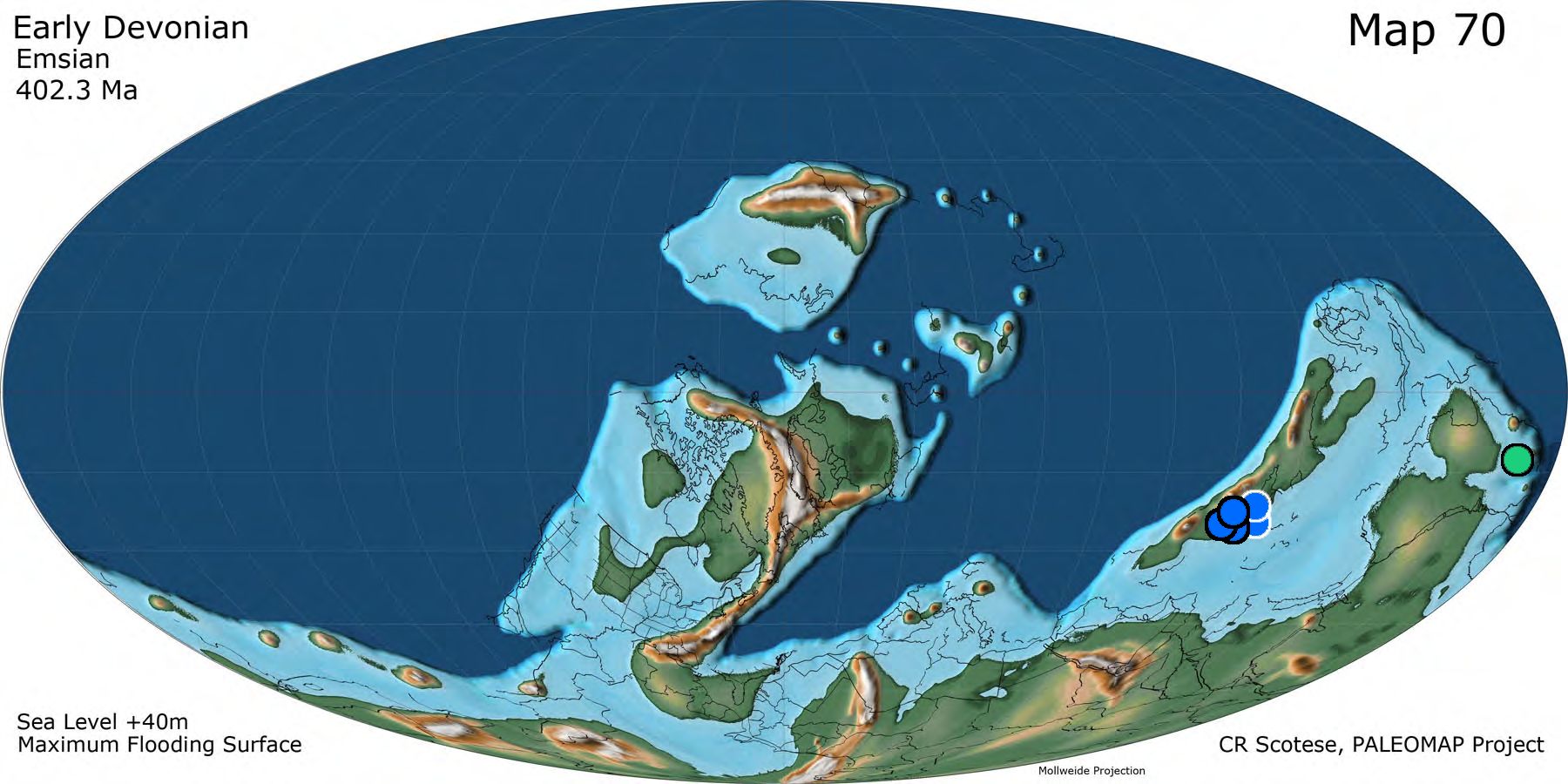


S3 Fig. Distribution of Bothriolepididae during the Emsian (palaeomap reprinted from [72] under a CC BY license, with permission from Christopher Scotese, original copyright 2014).

### Eifelian

Eifelian


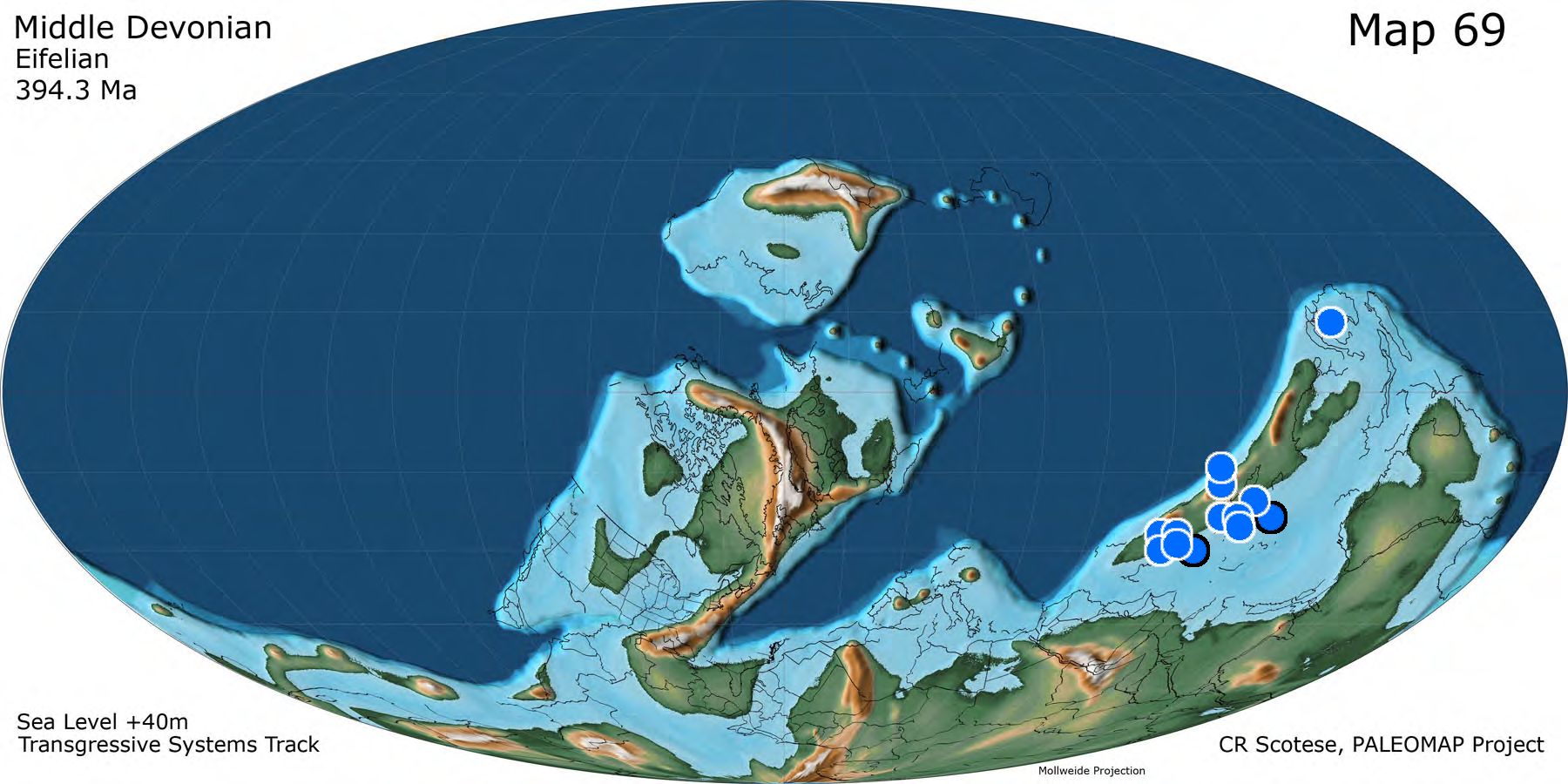


S4 Fig. Distribution of Bothriolepididae during the Eifelian (palaeomap reprinted from [72] under a CC BY license, with permission from Christopher Scotese, original copyright 2014).

### Givetian

Givetian


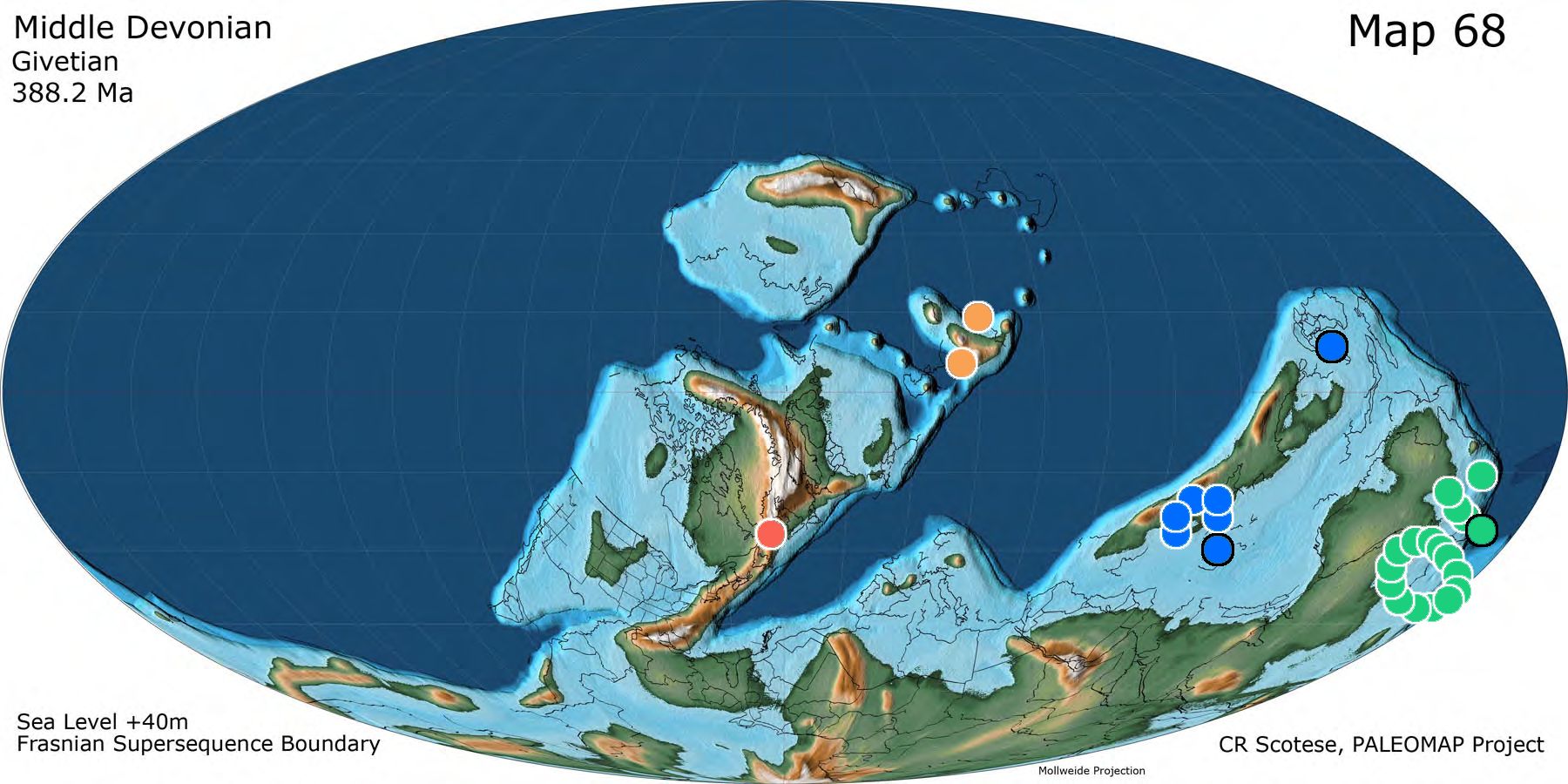


S5 Fig. Distribution of Bothriolepididae during the Givetian (palaeomap reprinted from [72] under a CC BY license, with permission from Christopher Scotese, original copyright 2014).

### Frasnian

Frasnian


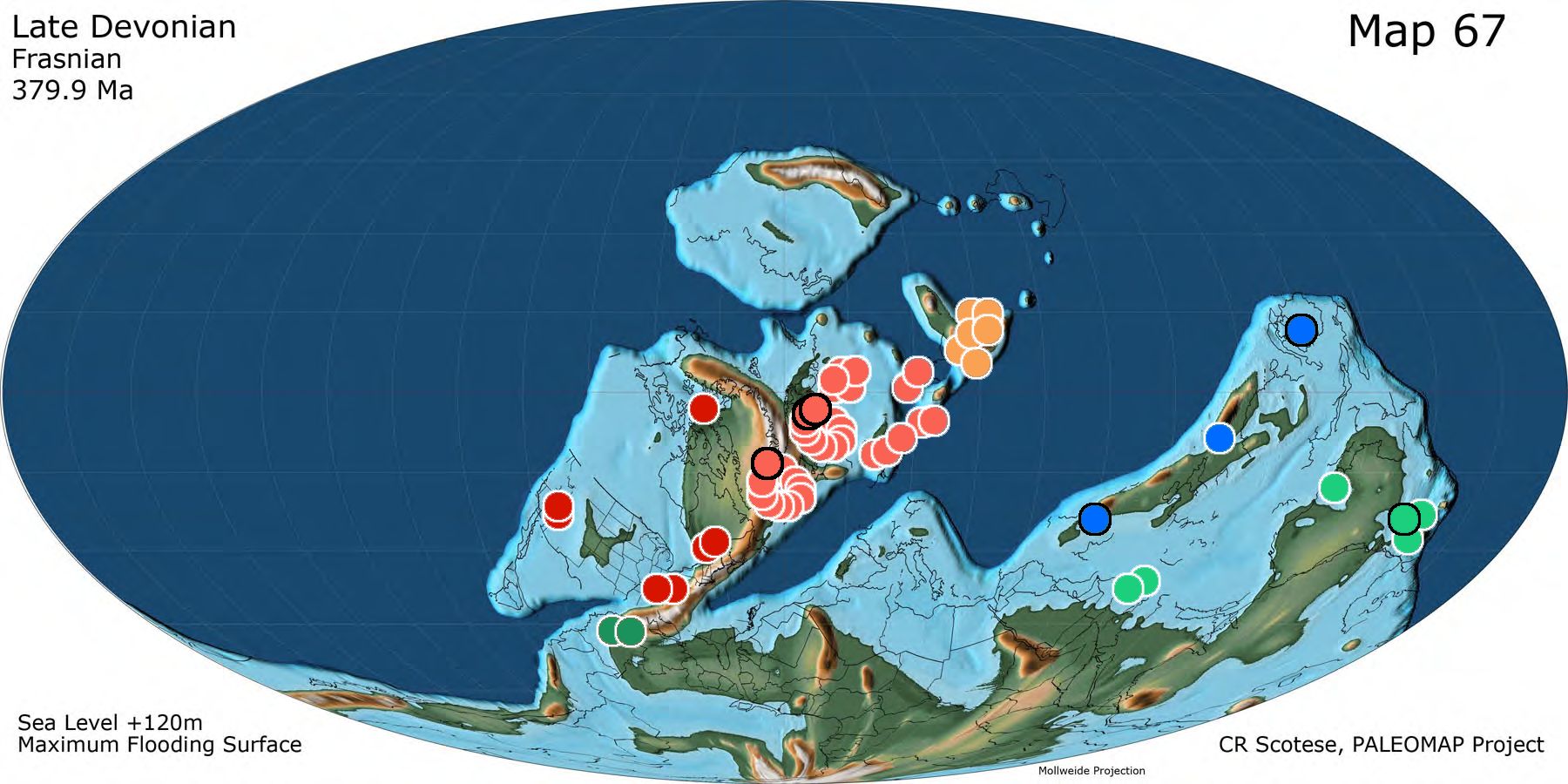


S6 Fig. Distribution of Bothriolepididae during the Frasnian (palaeomap reprinted from [72] under a CC BY license, with permission from Christopher Scotese, original copyright 2014).

### Famennian

Famennian


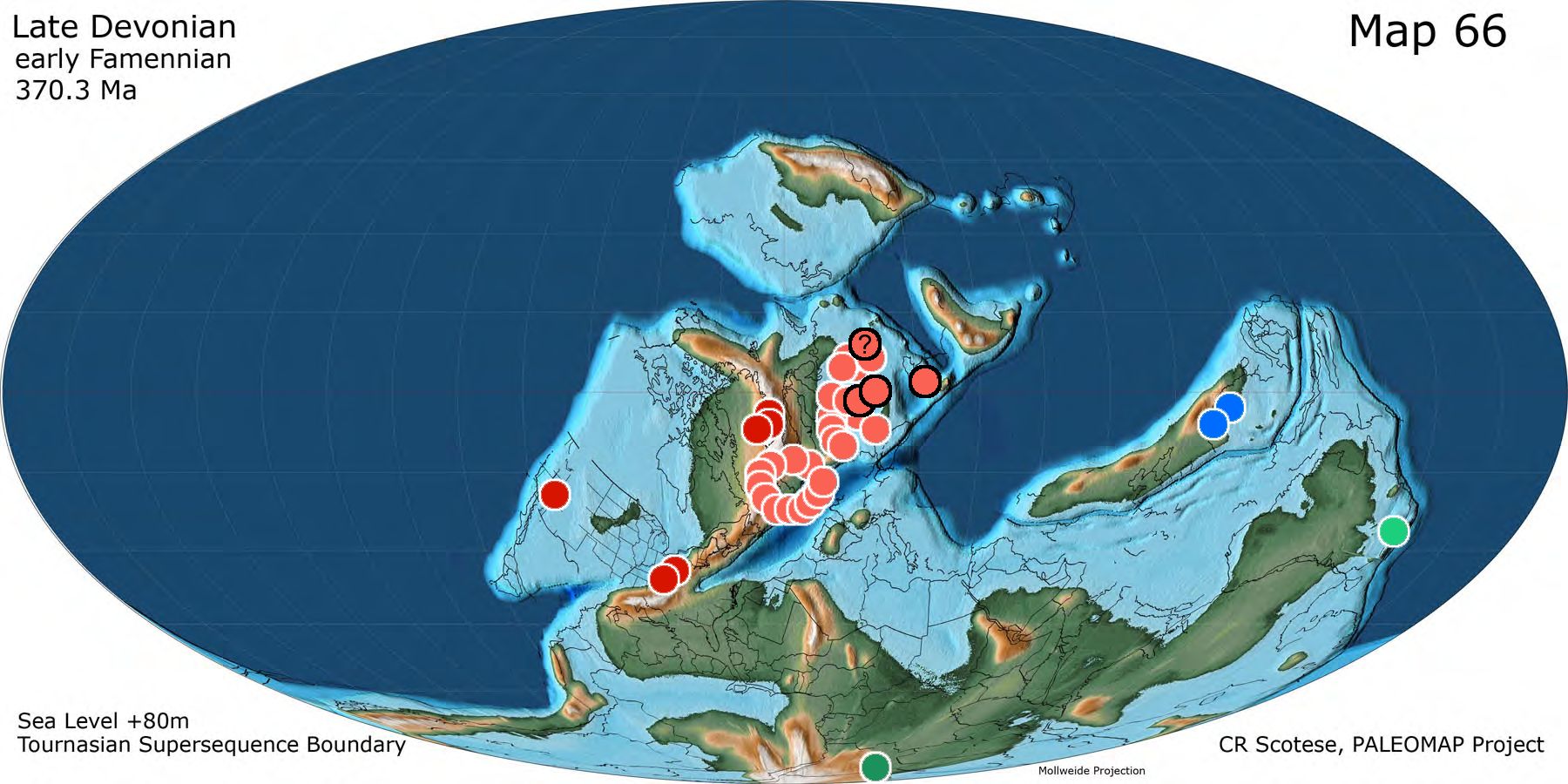


S7 Fig. Distribution of Bothriolepididae during the Famennian (palaeomap reprinted from [72] under a CC BY license, with permission from Christopher Scotese, original copyright 2014).

### Late Famennian


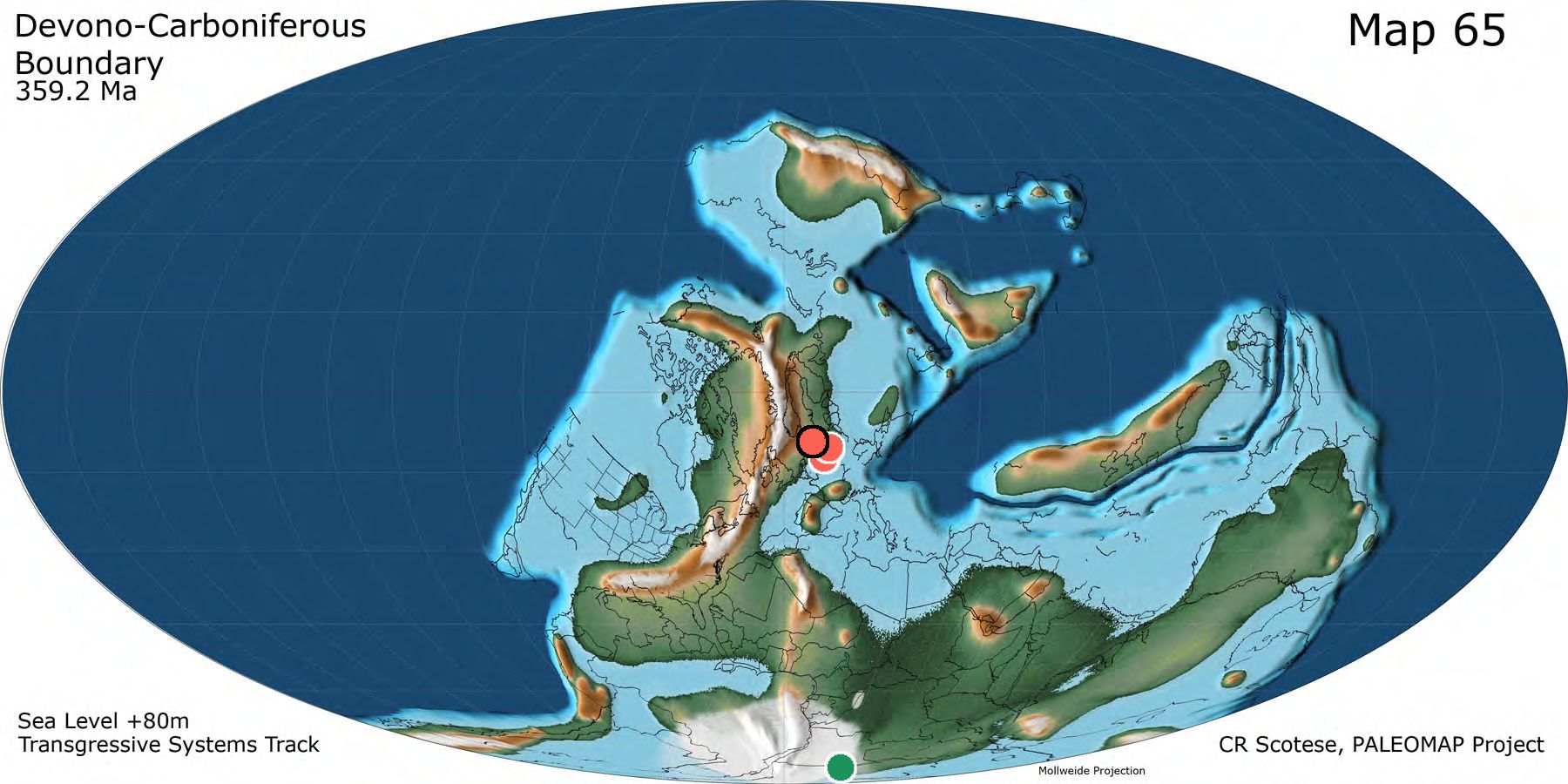


S8 Fig. Distribution of Bothriolepididae during the late Famennian (palaeomap reprinted from [72] under a CC BY license, with permission from Christopher Scotese, original copyright 2014).

## Bothriolepididae taxa, strata and localities

Some of the taxa below are considered as junior synonyms of others (see dedicated section in Supplementary Information); they are however mapped when they belong to different localities than their senior, and listed with their old name for convenience. Non-mapped species because same locality as their senior synonym are: *B. leidyi*, *B. retinata, B. stensioi.*

| **Taxon** | **Geography** | | | **Stratigraphy** | **Comments** |
| --- | --- | --- | --- | --- | --- |
|  | **Locality** | **Province / Area** | **Country** |  |  |
| **Bothriolepidoidei** |  |  |  |  |  |
| *Luquanolepis pileos* | Luquan | Yunnan | China | Emsian | in [76] |
|  |  |  |  |  |  |
| **Bothriolepididae** |  |  |  |  |  |
| Bothriolepididae | Tarim | Xinjiang | China | Frasnian | in [76] |
|  |  |  |  |  |  |
| ***Bothriolepis*** |  |  |  |  |  |
| *Bothriolepis africana* | Witpoort | South Africa | South Africa | Famennian | near Grahamstown, South Africa; in [60] |
| *Bothriolepis alexi* | Aztec | Victoria Land | East Antarctica | Givetian | in [77] |
| *Bothriolepis alvesiensis* | Rosebrae | Scotland | United Kingdom | Famennian | Early Frasnian in [77] (Upper Devonian, in [2]); corrected from Famennian to Frasnian on 20200605; back to Famennian on 20201102 based on Marshall in [78] |
| *Bothriolepis alvesiensis* | Alves and Rosebrae beds | Scotland | United Kingdom | Frasnian | Famennian in Denison, but corrected to Frasnian by [79] (Alves: mid-late Frasnian; Rosebrae: late Famennian) 20201102) |
| *Bothriolepis amankonyrica* | Central Kazakhstan | Central Kazakhstan | Kazakhstan | Frasnian | Central Kazakhstan, Olenty River, 60 km north of the village of Molodezhnyi, Amankonyr locality; Upper Devonian, Frasnian, basal Sofa Formation (in Moloshnikov, 2011) |
| *Bothriolepis antarctica* | Aztec | Victoria Land | East Antarctica | Middle Devonian | Middle or Upper Devonian, Beacon sandstone, in [2] - changed from Victoria Land into Aztec to fit [77] |
| *Bothriolepis askinae* | Aztec | Victoria Land | East Antarctica | Givetian | in [77] |
| *Bothriolepis askinae* | Aztec | Victoria Land | East Antarctica | Givetian | in [77] |
| *Bothriolepis babichevi* | Trudovoe | Central Kazakhstan | Kazakhstan | Givetian | Central Kazakhstan, north east of the city of Karaganda, right bank of the Shiderty River, village of Trudovoe; Middle Devonian, Givetian, Konyr Formation, lower subformation. In [80] |
| *Bothriolepis babichevi* | Trudovoe | Central Kazakhstan | Kazakhstan | Givetian | Central Kazakhstan, north east of the city of Karaganda, right bank of the Shiderty River, village of Trudovoe; Middle Devonian, Givetian, Konyr Formation, lower subformation (in [81]) |
| *Bothriolepis barretti* | Aztec | Victoria Land | East Antarctica | Givetian | in [77] |
| *Bothriolepis bindareei* | Mount Howitt | Victoria | Australia | Givetian |  |
| *Bothriolepis canadensis* | Miguasha | Québec (QBC) | Canada | Frasnian | Escuminac Fm., in [2]; [77] |
| *Bothriolepis cellulosa* | Baltic States | Baltic States | Baltic States | Frasnian | Snetogor stage, Baltic States + NW USSR, in [2]; Earliest Frasnian in [37] |
| *Bothriolepis cellulosa* | Timan | Timan | Russia | Frasnian | Timan and Tuva Depression, in [2]; Earliest Frasnian in [37] |
| *Bothriolepis cf. kwangungensis* | Lechang | Guangdong | China | Givetian | Dahepo Fm., in [76] |
| *Bothriolepis cf. kwangungensis* | Anfu | Jiangxi | China | Givetian | Dahepo Fm., in [76] |
| *Bothriolepis ciecere* | Ketleri | Latvia | Latvia | Famennian | in [77] |
| *Bothriolepis ciecere* | Pavari | Vidzeme | Latvia | Famennian | Upper Ketleri stage, in [2]; locality name from [82] |
| *Bothriolepis coloradensis* | Chaffee | Colorado (CO) | U.S.A. | Famennian | Chaffee Fm., in [2, 38] but noted as Frasnian; relabelled as Famennian following [77]; species is synonym of *B. nitida* ([13]) |
| *Bothriolepis coloradensis* | La Plata County | Colorado (CO) | U.S.A. | Frasnian | Elbert Fm., Colorado, in [2, 38]; species is synonym of *B. nitida* ([13]) |
| *Bothriolepis cristata* | Dura Den | Scotland | United Kingdom | Famennian | in [2]; in [77] |
| *Bothriolepis cristata* | Rosebrae | Scotland | United Kingdom | Famennian | in [2]; in [77] |
| *Bothriolepis cullodenensis* | Mount Howitt | Victoria | Australia | Givetian | in [77] |
| *Bothriolepis curonica* | Latvia | Latvia | Latvia | Frasnian | [37] |
| *Bothriolepis dairbhrensis* | Valentia Island | Kerry | Ireland | Givetian | Current article |
| *Bothriolepis darbiensis* | Fort Washakie | Wyoming (WY) | U.S.A. | Frasnian | in Denison, 1951, 1978 |
| *Bothriolepis darbiensis* | Teton Pass | Wyoming (WY) | U.S.A. | Frasnian | in Denison, 1951, 1978 |
| *Bothriolepis dorakarasungensis* | Tuva Basin | Tuva Republic | Russia | Frasnian | central Tuva, upper reaches of the Dora-Kara-Sug River; Upper Devonian, Frasnian, Kokhai Formation ([69]) |
| *Bothriolepis evaldi* | Kalnamuiza | Kandava | Latvia | Frasnian | Middle Frasnian according to [37] |
| *Bothriolepis evaldi* | Kaibala | Lielvarde | Latvia | Frasnian | Middle Frasnian according to [37] |
| *Bothriolepis favosa* | Baltic States | Baltic States | Baltic States | Frasnian | Snetogor and Shelon-Ilmen stages, Baltic states and western USSR, in [2] |
| *Bothriolepis fergusoni* | Mount Howitt | Victoria | Australia | Givetian | in [77] |
| *Bothriolepis gigantea* | Alves beds | Scotland | United Kingdom | Frasnian | mid-late Frasnian in [79] (modified from Famennian in [2]) |
| *Bothriolepis gippslandiensis* | Mount Howitt | Victoria | Australia | Givetian | in [77] |
| *Bothriolepis grenfellensis* | Grenfell | New South Wales | Australia | Famennian | in [77] |
| *Bothriolepis groenlandica* | Greenland | Greenland | Greenland | Famennian | *Phyllolepis* series, East Greenland, in [2]; early Famennian in [83] |
| *Bothriolepis hayi* | Oxendean beds | Scotland | United Kingdom | Famennian | Upper Devonian, in [2]; not more precise in [84], although lowest member containing *B. hicklingi* is dated as Famennian (based on *Bothriolepis* sp. and *Holoptychius*) |
| *Bothriolepis heckeri* | Bilovo | Novgorod Oblast | Russia | Famennian | Bilovo Beds? (corresponding to Akemene beds/Fm., so just below mid-Famennian in [37]) |
| *Bothriolepis hicklingi* | Redheugh Mudstones | Scotland | United Kingdom | Famennian | Upper Old Red Sandstones in [2]; [84] propose a Famennian Age |
| *Bothriolepis hydrophila* | Portishead | England | United Kingdom | Famennian | in [77] |
| *Bothriolepis hydrophila* | Dura Den | Scotland | United Kingdom | Famennian | in [77]; Upper Devonian in [2] |
| *Bothriolepis jani* | Tervete | Tervete | Latvia | Famennian | in [77] |
| *Bothriolepis jarviki* | Greenland | Greenland | Greenland | Famennian | Phyllolepis series, East Greenland, in [2]; end Frasnian - early Famennian in [83] |
| *Bothriolepis jazwicensis* | Cheiloceras beds | Holy Cross Mountain | Poland | Famennian | in [77] |
| *Bothriolepis jeremijevi* | Timan | Timan | Russia | Upper Devonian | poorly known and undefinable, in [2]; reassigned to *B. leptocheira jeremijevi* by [17] |
| *Bothriolepis karawaka* | Aztec | Victoria Land | East Antarctica | Givetian | in [77] |
| *Bothriolepis kassini* | Central Kazakhstan | Central Kazakhstan | Kazakhstan | Frasnian | central Kazakhstan, right bank of the Shiderty River, Krasnyi Pakhar’ locality; Upper Devonian, Frasnian, Shatskaya Group, basal Sofa Formation. In [85] |
| *Bothriolepis kassini* | Trudovoe | Central Kazakhstan | Kazakhstan | Givetian | Komyr Fm. Revised by [85] (after Malinovskaya's works) |
| *Bothriolepis kohni* | Aztec | Victoria Land | East Antarctica | Givetian | in [77] |
| *Bothriolepis kwangtungensis* | Lechang | Guangdong | China | Givetian | in [2]; [76] |
| *Bothriolepis kwangtungensis* | Anfu | Jiangxi | China | Givetian | in [2]; [76] |
| *Bothriolepis laverocklochensis* | Rosebrae | Scotland | United Kingdom | Famennian | in [77] (upper Devonian in [2]) |
| *Bothriolepis leptocheira* | Gauja | Lode | Latvia | Famennian | in [77]; subspecies *B. leptocheira curonica* according to [37]; age changed from Givetian ([77], who entered it as *B. curonica*) to Famennian ([37]) |
| *Bothriolepis leptocheira* | Latvia | Latvia | Latvia | Frasnian | Horizon e, Latvia, in [2] (subspecies *B. leptocheira curonica* after [37]) |
| *Bothriolepis leptocheira* | Upper Old Red Sandstones (Scotland) | Scotland | United Kingdom | Upper Devonian | in [2] |
| *Bothriolepis leptocheira curonica* | Gauja | Lode | Latvia | Famennian | in [77]; subspecies *B. leptocheira curonica* according to [37]; age changed from Givetian (Sallan & Coates 2010, who entered it as *B. curonica*) to Famennian ([37]) |
| *Bothriolepis leptocheira curonica* | Latvia | Latvia | Latvia | Frasnian | Very dubious stratigraphy, not mentioned by Lukševičs: Horizon e, Latvia, in [2] (subspecies *B. leptocheira curonica* after [37]) |
| *Bothriolepis leptocheira curonica* | Upper Old Red Sandstones (Scotland) | Scotland | United Kingdom | Upper Devonian | in [2] |
| *Bothriolepis leptocheira jeremejevi* | Timan | Timan | Russia | Famennian | earliest Famennian of Sosnogorsk Fm., South Timan (Komi Republic), new subspecies combination by [17] |
| *Bothriolepis leptocheira ssp.* | Severnaya Zemlya | Siberia | Russia | Famennian | in [37], [17] |
| *Bothriolepis lochangensis* | Lechang | Guangdong | China | Givetian | in [2]; [76] |
| *Bothriolepis lochangensis* | Anfu | Jiangxi | China | Givetian | in [2]; [76] |
| *Bothriolepis lohesti* | Chèvremont | Wallonie | Belgium | Famennian | [86] |
| *Bothriolepis lohesti* | Evieux | Wallonie | Belgium | Famennian | in [77]; obviously duplicate of several localities in the Evieux Fm |
| *Bothriolepis lohesti* | Vaux-sous-Chèvremont | Wallonie | Belgium | Famennian | [86] |
| *Bothriolepis longi* | Nettleton's Creek | New South Wales | Australia | Middle - Upper Devonian | Givetian or Frasnian according to [63]; strong affinities with Aztec fauna (Antarctica) |
| *Bothriolepis macphersoni* | Aztec | Victoria Land | East Antarctica | Givetian | in [77] |
| *Bothriolepis macrocephala* | Upper Old Red Sandstones (England) | England | United Kingdom | Upper Devonian | in [2] |
| *Bothriolepis maeandrina* | Timan | Timan | Russia | Upper Devonian | in [2] |
| *Bothriolepis major* | Edenkillie beds | Scotland | United Kingdom | Frasnian | in [2] |
| *Bothriolepis markovskii* | Serpievka | Ural | Russia | Frasnian | southern Ural Mountains, Chelyabinsk Region, IvanKatavskii District, Sim River Basin, village of Serpievka; Upper Devonian, Upper Frasnian, Askyn Regional Stage, Orlovka Formation (in [80]) |
| *Bothriolepis mawsoni* | Aztec | Victoria Land | East Antarctica | Givetian | in [77] |
| *Bothriolepis maxima* | Baltic States | Baltic States | Baltic States | Frasnian | "Baltic States and northwestren USSR", in [2]; see also [37] for mid-Late Franian age |
| *Bothriolepis minor* | Oneonta Formation | New York (NY) | U.S.A. | Upper Devonian | in [2] |
| *Bothriolepis minor* | Chemung | Pennsylvania (PA) | U.S.A. | Upper Devonian | in [2] |
| *Bothriolepis minor* | Hyner | Pennsylvania (PA) | U.S.A. | Upper Devonian | in [2]; in [77] (Late Famennian) |
| *Bothriolepis nielseni* | Aina Dal | Greenland | Greenland | Famennian | East Greenland, Remigolepis series; in [2] |
| *Bothriolepis nikitinae* | Central Kazakhstan | Central Kazakhstan | Kazakhstan | Frasnian | Central Kazakhstan, right bank of the Shiderty River, 75 km northeast of the village of Molodezhnyi, Krasnyi Pakhar’ locality; Upper Devonian, Frasnian, basal Sofa Formation ([80]). |
| *Bothriolepis nitida* | Blossburg | Pennsylvania (PA) | U.S.A. | Famennian | Catskill Formation, Catskill Group, NY state and Pennsylvania, in [2]; in [13] |
| *Bothriolepis nitida* | Hyner | Pennsylvania (PA) | U.S.A. | Famennian | in [77] |
| *Bothriolepis nitida* | Leroy | Pennsylvania (PA) | U.S.A. | Famennian | Catskill Formation, Catskill Group, NY state and Pennsylvania, in [2]; in [13] |
| *Bothriolepis niushoushanensis* | Zhongning | Ningxia | China | Eifelian | strati in [76]- |
| *Bothriolepis obesa* | Jedburgh beds | Scotland | United Kingdom | Upper Devonian | in [2] |
| *Bothriolepis obrutschewi* | Baltic States | Baltic States | Baltic States | Frasnian | Podsnetogor (Amata) stage; Baltic States, USSR, in [2]. Considered end-Givetian in [37] (Amata regional stage). |
| *Bothriolepis ornata* | Tervete | Tervete | Latvia | Famennian | in [77] |
| *Bothriolepis ornata* | Leningrad | Russia | Russia | Famennian | Leningrad Area, Nadbilovo stage, in [2] |
| *Bothriolepis ornata* | Timan | Timan | Russia | Famennian | in [2] |
| *Bothriolepis panderi* | Wietrznia | Holy Cross Mountain | Poland | Frasnian | in [77]; synonym of *B. favosa* in [2] |
| *Bothriolepis paradoxa* | Scaat Craig | Scotland | United Kingdom | Famennian | Upper Devonian in [2]; Famennian in [77]. Scaat Craig beds corresponds to a Low level of Alves beds ([87]) |
| *Bothriolepis paradoxa* | Alves beds | Scotland | United Kingdom | Upper Devonian | in [2] |
| *Bothriolepis pavariensis* | Latvia | Latvia | Latvia | Famennian | Upper Ketleri stage, Latvia, in [2] |
| *Bothriolepis perija* | Cano Colorado | Zulia | Venezuela | Frasnian | early mid Frasnian ([66]:164) |
| *Bothriolepis portalensis* | Aztec | Victoria Land | East Antarctica | Givetian | in [77] |
| *Bothriolepis prima* | Armenia | Armenia | Armenia | Frasnian | doubtful, in [2] |
| *Bothriolepis prima* | Latvia | Latvia | Latvia | Frasnian | Podsnetogor (Amata) beds; in [2]. Considered end-Givetian in [37] (Amata regional stage). |
| *Bothriolepis rex* | Ellesmere Island | Nunavut (NUN) | Canada | Frasnian | [71] |
| *Bothriolepis sanzarensis* | Samarkand | Samarkand | Uzbekistan | Frasnian | Uzbekistan, vicinity of Samarkand, western marginal area of the Turkestan Mountain Range, Malguzar Mountains, Sanzar River Basin, village of Katta_Kara_Shak_Shak; Upper Devonian, Frasnian, Upper Frasnian Substage (in [80]) |
| *Bothriolepis shaokuanensis* | Shaoguan | Guangdong | China | Eifelian | in [2]; [76] |
| *Bothriolepis siberica* | Oryol | Oryol | Russia | Famennian | in [77] |
| *Bothriolepis siberica* | Oryol | Oryol | Russia | Famennian | in [77] |
| *Bothriolepis siberica* | Minusinsk Basin | Siberia | Russia | Frasnian | Kokhai formation; in [2] |
| *Bothriolepis siberica* | Tuva Basin | Tuva Republic | Russia | Frasnian | Kokhai formation; in [2] |
| *Bothriolepis sinensis* | Changsha | Hunan | China | Eifelian | in [76]--Tiaomanjian Fm. |
| *Bothriolepis sinensis* | Yiyang | Hunan | China | Eifelian | in [76]--Tiaomanjian Fm. |
| *Bothriolepis sinensis* | Kunming | Yunnan | China | Eifelian | Haikou Fm; in [76]. |
| *Bothriolepis sosnensis* | Livny | Oryol | Russia | Famennian | [67] |
| *Bothriolepis stevensoni* | Upper Old Red Sandstones (Scotland) | Scotland | United Kingdom | Upper Devonian | in [2] |
| *Bothriolepis tastenica* | Central Kazakhstan | Central Kazakhstan | Kazakhstan | Frasnian | Central Kazakhstan, right bank of the Shiderty River, 65 km east of the village of Molodeznyi, Saumalkol’ locality; Upper Devonian, Frasnian, middle part of the Sofa Formation; in [81] |
| *Bothriolepis tatongensis* | Tatong | Victoria | Australia | Frasnian | late Givetian - early Frasnian in [59] |
| *Bothriolepis tatongensis* | Tatong | Victoria | Australia | Givetian | late Givetian - early Frasnian in [59] |
| *Bothriolepis taylori* | Edenkillie beds | Scotland | United Kingdom | Frasnian | in [2] |
| *Bothriolepis traquairi* | Miguasha | Québec (QBC) | Canada | Frasnian | Escuminac Fm., in [2]; [77] |
| *Bothriolepis traudscholdi* | Stolbovo | Russia | Russia | Frasnian | in [77] |
| *Bothriolepis tungseni* | Qujing | Yunnan | China | Eifelian | in [76] |
| *Bothriolepis tungseni* | Wuding | Yunnan | China | Eifelian | in [76] |
| *Bothriolepis tungseni* | Haikou | Yunnan | China | Givetian | in [77] |
| *Bothriolepis turanica* | ZaktanSai | Tien Shan | Uzbekistan | Frasnian | in [2]; [80] |
| *Bothriolepis virginiensis* | Chemung | Pennsylvania (PA) | U.S.A. | Upper Devonian | Upper Devonian in [51] |
| *Bothriolepis volongensis* | Timan | Timan | Russia | Frasnian | upper part of Rassokha Fm. (in [64]) |
| *Bothriolepis vuwae* | Aztec | Victoria Land | East Antarctica | Givetian | in [77] |
| *Bothriolepis wilsoni* | Harelaw beds | Scotland | United Kingdom | Upper Devonian | in [2]; Upper part of the Upper Old Red Sandstone Lithofacies in [84]) |
| *Bothriolepis yeungae* | Canowindra | New South Wales | Australia | Famennian | in [77] |
| *Bothriolepis yunnanensis* | Wuding | Yunnan | China | Eifelian | in [2]; [76] |
| *Bothriolepis sp.* | Bobai | Guangxi | China | Eifelian | in [76] |
| *Bothriolepis sp.* | Qujing | Yunnan | China | Eifelian | in [76] |
| *Bothriolepis sp.* | Wuding | Yunnan | China | Eifelian | in [76] |
| *Bothriolepis sp.* | Qujing | Yunnan | China | Emsian | in [76] |
| *Bothriolepis sp.* | Wuding | Yunnan | China | Emsian | in [76] |
| *Bothriolepis sp.* | Evieux | Wallonie | Belgium | Famennian | in [77]; obviously duplicate of several localities in the Evieux Fm. (Modave in [86]) |
| *Bothriolepis sp.* | Lengshuijiang | Hunan | China | Famennian | in [76] |
| *Bothriolepis sp.* | Liuyang | Hunan | China | Famennian | in [76] |
| *Bothriolepis sp.* | Andreyevka-2 | Siberia | Russia | Famennian | in [77] |
| *Bothriolepis sp.* | Gogo formation | Western Australia | Australia | Frasnian | in [77] |
| *Bothriolepis sp.* | Taojiang | Hunan | China | Frasnian | in [76] |
| *Bothriolepis sp.* | Floresta | Boyaca | Colombia | Frasnian | in [88] |
| *Bothriolepis sp.* | Chahriseh | Isfahan | Iran | Frasnian | in [77] |
| *Bothriolepis sp.* | Kerman | Kerman | Iran | Frasnian | in [77] |
| *Bothriolepis sp.* | Taishan | Guangdong | China | Givetian | in [76] |
| *Bothriolepis sp.* | Rockport | Michigan (MI) | U.S.A. | Givetian | in [77]; reattributed to *Protitanichthys rockportensis* by [89] |
|  |  |  |  |  |  |
| ***Briagalepis*** |  |  |  |  |  |
| *Briagalepis warreni* | Mount Howitt | Victoria | Australia | Middle - Upper Devonian | From the lower site at Freestone Creek, within the sediments of the Wellington Rhyolites, and from the upper conglomerate of the Bindaree -- the Bluff section of the Avon River Gp ([90], p. 100) cropping out along Bindaree Road, near Mt Howitt (in [59]). |
|  |  |  |  |  |  |
| ***Dianolepis*** |  |  |  |  |  |
| *Dianolepis liui* | Qujing | Yunnan | China | Eifelian | in [2]; [76] |
| *Dianolepis liui* | Wuding | Yunnan | China | Eifelian | in [2]; [76] |
| *Dianolepis liui* | Haikou | Yunnan | China | Givetian | in [77] |
|  |  |  |  |  |  |
| ***Grossilepis*** |  |  |  |  |  |
| *Grossilepis brandi* | Hazeldean Burn Beds | Scotland | United Kingdom | Frasnian | in [2] |
| *Grossilepis rikiki* | Strud | Wallonie | Belgium | Famennian |  |
| *Grossilepis spinosa* | Gauja | Lode | Latvia | Frasnian | Sallan and Coates (77) mentioned a Givetian age, but Lukševičs (37) mentioned a middle Frasnian age (Pamūšis regional stage, favoured here) |
| *Grossilepis tuberculata* | Snetnaya Gora Beds | Latvia | Latvia | Frasnian | in [2] |
| *Grossilepis tuberculata* | Minusinsk Basin | Siberia | Russia | Upper Devonian | in [2], but doubtful according to him |
|  |  |  |  |  |  |
| ***Houershanoelepis*** |  |  |  |  |  |
| *Houershanolepis changi* | Dushan | Guizhou | China | Early Devonian | Mount Houershan in Dushu county; based on a single AMD plate; [91] |
|  |  |  |  |  |  |
| ***Livnolepis*** |  |  |  |  |  |
| *Livnolepis zadonica* | Oryol | Oryol | Russia | Famennian | in [77] |
|  |  |  |  |  |  |
| ***Monarolepis*** |  |  |  |  |  |
| *Monarolepis verrucosa* | Taemas Wee Jasper | New South Wales | Australia | Emsian | [25, 50] |
|  |  |  |  |  |  |
| ***Rossolepis*** |  |  |  |  |  |
| *Rossolepis brodensis* | Oryol | Oryol | Russia | Famennian | in [77] |
|  |  |  |  |  |  |
| ***Vietnamaspis*** |  |  |  |  |  |
| *Vietnamaspis trii* | Do Son | North Vietnam | Vietnam | Middle - Upper Devonian | [54] |
|  |  |  |  |  |  |
| ***Wudinolepis*** |  |  |  |  |  |
| *Wudinolepis weni* | Wuding | Yunnan | China | Emsian | in [76] |
|  |  |  |  |  |  |
| ***Wufensgshania*** |  |  |  |  |  |
| *Wufengshania magniforaminis* | Wuding | Yunnan | China | Emsian | [92] |

Table B. List of Bothriolepididae with stratigraphic and geographic indications.

# Data matrix taxa * characters

## Data matrix

The data matrix with complete and reduced list of characters (see below) and indices (character CI and RI, taxon and character completeness and polymorphism) is given as separate XL file. Mesquite-, TNT-, and PAUP-formatted files corresponding to the different steps and results of the phylogenetic analyses are given as Supplementary Files. All analyses were run on a portable DELL PC running Windows 10 64 bits; Compatibility with reader’s operating system not guaranteed.

## List of characters

### Original complete set

Although unused, this list is provided as a basis for further studies.

1. shape of preorbital recess ([50]:text-fig. 68, character 27e, i, r)

0: absent

1: simple and rounded (see *B. askini* in [50])

2: trilobate (clover; see *B. barretti* in [50])

3: pentagonal (spades; see *B. portalensis* in [50])

4: trifid (see *B. nitida* in [50])

5: trapezoidal (see *Livnolepis* (*Bothriolepis*) *zadonica* in [93])

2. anterior extension of postorbital process (new)

0: remains behind orbital fenestra

1: extends beyond orbital fenestra anteriorly

3. skull roof shape (for adults only; new)

0 round

1 quadrangular

2 hexagonal (with pointed anterior tip)

3 heptagonal

4. orbitonasal fenestra shape (new)

0 anterior and posterior edges straight and parallel

1 anteriorly concave and posteriorly convex (inversed bean)

2 anteriorly convex and posteriorly concave (bean)

3 concave anteriorly and posteriorly (peanut shaped)

4 convex anteriorly and posteriorly (rounded)

5. ratio length anterior tip / width between lateral corners (new)

This characters is difficult to assess with animals reconstructed flattened or in 3d (width shorter)

calculation relatively consistent throughout *Bothriolepis* species: 0,6<L/W<0,9 (new)

6. preorbital region of skull

0:short

1: elongated

Character unifying *Grossilepis* and *Bothriolepis* (contra other antiarchs) ([50]:text-fig. 68, character 27l)

7. bone thickness on orbital edges of PrM and La

0: even with rest of the plates

1: thicker at orbital edge ([50]:text-fig. 68, character 27o; [37]:char 8)

8. mesial lamina vs ventral lamina of La and PaN (vs. ioc) (new)

0 same level

1 lag

9. position of lateral corner of skull roof relative to PM-La-PaN point (new)

0 corner anterior

1 same level

2 corner posterior

10. position of posterior edge of orbit (contact PPi-Nu-orb) compared to position of La-PM-PaN plate (new)

0 orbit anterior

1 same level

2 orbit posterior

11. posterolateral indentation on PM and PAN for thoracic armour (new)

0: unmarked, rather flat (see *B. askini* in [50])

1: well marked (see *B. portalensis* in [50])

12. Lateral edge of premedian plate (new)

0 straight

1 concave

2 convex

13. Premedian plate posterior (new)

0 flat / straight

1 convex

2 concave

14. Premedian plate ratio anterior edge/mesial constriction width (new)

0 <1

1 =1

2 >1

15. Premedian plate ratio anterior edge/posterior edge (new)

0 <1

1 =1

2 >1

16. Premedian plate ratio mesial constriction width / posterior edge (new)

0 <1

1 =1

2 >1

17. lateral pits (internal side of La)

0: small

1: enlarged ([50]:text-fig. 68, character 27d)

18. separate ventrally facing attachment surface for PreLateral on Lateral plate

0: absent

1: present ([50]:text-fig. 68, character 27m)

19. Orbital margin of postpineal PPi at the level of lateral corners of skull roof (modified from [37]:char 18; was binary character)

0 lateral corner anterior to PPi orbital margin

1 same level

2 lateral corner posterior to PPi orbital margin

20. postpineal plate anterior edge (new)

0 concave

1 flat

2 convex

21. postpineal plate (new)

0 without lateral flanges

1 with anterior flanges

22. Nuchal orbital edge (new)

0 absent

1 present

23. Nuchal plate shape #1 ([50]:text-fig. 68, character 27p, modified and split into 3 characters)

0: wider than long

1: longer than wide

2: as wide as long

24. Nuchal plate shape #2 ([50]:text-fig. 68, character 27p, split into 3 characters)

0: contact edge with La straight

1: contact edge with La convex

25. Nuchal plate shape #3 (modified from [50]:text-fig. 68, character 27p. split into 3 characters; original states reversed)

0 long posterolateral corners

1 short posterolateral corners

26. shape contact Nu/PaN (new)

This character is a possible alternative to [50] (character p in text.-fig. 68) and [37] (character 13)

0 straight

1 Nu convex

2 Nu concave

3 sigmoid

27. Nuchal plate ratio width intermesiolateral edge / width interoposterolateral edge (new)

0 <1

1 =1

2 >1

28. ratio length anterolateral margin (Nu-La) / length posterolateral margin (Nu-PaN) (new)

0 <1

1 =1

2 >1

29. Nuchal plate ratio anterolateral edge/posterolateral edge length (in [63]:char 10); [37]:char 13)

0

1 squarish Nu with convex anterior division of the lateral margin and short posterolateral corners

30. obtected nuchal area present on PaN ([37]:char 6)

0 absent

1 present

31. Shape of postmarginal plate (similar to [37]:char 15)

0 triangular

1 quadrangular

2 pentagonal (extra angle on edge with La)

32. Ratio Length/Width of postmarginal plate (new)

0 <1

1 =1

2 >1

33. SM plate shape ([37]:char 10 pro parte; [50]:text-fig. 68, character 27g)

0: long and shallow

1: short and deep

34. anterior SM plate attachment on Lateral plate as a transverse ridge ([50]:text-fig. 68, character 27j)

0: no

1: yes (in transverse lateral groove)

35. anterior portion of submarginal attachment covers spiracular groove ([63])

0 no

1 yes

36. AMD width: broadest at lateral angles in adults ([50]:text-fig. 68, character 27b); [37]:char 1)

0: no

1: yes

37. lateral angles of AMD appear early in ontogeny ([50]:text-fig. 68, character 27c; also in [37]:char 2)

0: no

1: yes

38. Median Dorsal plates crest ([50]:text-fig. 68, character 27f); [37]:char 17))

0: none

1: minor

2: well marked

39. margin AMD-ADL (new)

0 straight

1 sigmoid / sinuous

40. margin AMD-MxL (new)

0 straight

1 sigmoid / sinuous

41. MxL broadest at its dorsal corners in adults ([50]:text-fig. 68, character 27b)

0: no

1: yes

42. crista transversalis interna anterior transversally oriented on ventral lamina of AVL ([50]:text-fig. 68, character 27n; [37]:char 11)

0: no

1: yes

43. ventrolateral ridge makes a keel ([37]:char 20)

0 no

1 yes

44. axillary foramen shape ([50]:text-fig. 68, character 27k; [37]:character 4)

0: higher than long

1: longer than high

2: as long as high

45. contact Cv1, Cv2, Mm1, Mm2 in pectoral fin ([50]:text-fig. 68, character 27h; [37]:char 3)

0 all contact in one point

1 Mm1+Cv2 separate Cv1 from Mm2

2 Cv1 and Mm2 contact

46. Cd5 on distal segment ([37]:char 14)

0 absent

1 present

47. middle pit-line long ([37]:char 9 pro parte)

0 absent

1 present

48. supra-occipital sensory groove long ([37]:char 9 pro parte)

0 absent

1 present

49. branch of infraorbital sensory line diverging on PrL parallel to the rostral margin of the head-shield (also in [37]:char 12)

0 no

1 yes

50. main lateral line canal/groove reaches the posterior margin of the MxL plate ([37]:char 16)

0 no

1 yes

51. Long branch of the infraorbital sensory groove/canal diverging on PrL (also in [37]:char 19)

0 no

1 yes

52. Junction of rostral ifc in midline on PRM (new)

0 straight

1 light posterior indet

2 very deep posterior indent

53. Semi-circular groove on La (new)

0 reduced

1 developed

54. central sensory groove shape (new)

0 straight

1 curved

55. central sensory groove on Nu (new)

0 only Nu

1 extends on La

56. central sensory groove (new; slightly different from character 42 about extension of csc on Nu only or onto La)

0 directed to semicircular groove

1 directed to La/PaNu/Nu point

57. Pectoral pit-line traced on the Cv1 continuing on the Cv2 ([37]:char 5)

0 absent

1 present

58. dermal ornamentation ([50]:text-fig. 68, character 27a)

0: reticulate in juveniles only

1: reticulate in adults only

2: reticulate in juveniles and adults

59. general size and ornamentation

No state coded: Young says large size, vermiculate ornamentation, small orbital fenestra and elongate nuchal for cluster *wilsoni* + *mawsoni*] but no definite information for other taxa ([50]:text-fig. 68, character 27q)

### Final reduced set

1. shape of preorbital recess ([50]:text-fig. 68, character 27e, i, r)

0: absent

1: simple and rounded (see *B. askini* in [50])

2: trilobate (clover; see *B. barretti* in [50])

3: pentagonal (spades; see *B. portalensis* in [50])

4: trifid (see *B. nitida* in [50])

5: trapezoidal (see *Livnolepis* (*Bothriolepis*) *zadonica* in [93])

2. anterior extension of postorbital process (new)

0: remains behind orbital fenestra

1: extends beyond orbital fenestra anteriorly

3. skull roof shape (for adults only; new)

0 round

1 quadrangular

2 hexagonal (with pointed anterior tip)

3 heptagonal

4. orbitonasal fenestra shape (new)

0 anterior and posterior edges straight and parallel

1 anteriorly concave and posteriorly convex (inversed bean)

2 anteriorly convex and posteriorly concave (bean)

3 concave anteriorly and posteriorly (peanut shaped)

4 convex anteriorly and posteriorly (rounded)

5. preorbital region of skull

0:short

1: elongated

Character unifying *Grossilepis* and *Bothriolepis* (contra other antiarchs) ([50]:text-fig. 68, character 27l)

6. mesial lamina vs ventral lamina of La and PaN (vs. ioc) (new)

0 same level

1 lag

7. position of lateral corner of skull roof relative to PM-La-PaN point (new)

0 corner anterior

1 same level

2 corner posterior

8. position of posterior edge of orbit (contact PPi-Nu-orb) compared to position of La-PM-PaN plate (new)

0 orbit anterior

1 same level

2 orbit posterior

9. posterolateral indentation on PM and PAN for thoracic armour (new)

0: unmarked, rather flat (see *B. askini* in [50])

1: well marked (see *B. portalensis* in [50])

10. Lateral edge of premedian plate (new)

0 straight

1 concave

2 convex

11. Premedian plate posterior (new)

0 flat / straight

1 convex

2 concave

12. Premedian plate ratio anterior edge/mesial constriction width (new)

0 <1

1 =1

2 >1

13. Premedian plate ratio anterior edge/posterior edge (new)

0 <1

1 =1

2 >1

14. Premedian plate ratio mesial constriction width / posterior edge (new)

0 <1

1 =1

2 >1

15. lateral pits (internal side of La)

0: small

1: enlarged ([50]:text-fig. 68, character 27d)

16. Orbital margin of postpineal PPi at the level of lateral corners of skull roof (modified from [37]:char 18; was binary character)

0 lateral corner anterior to PPi orbital margin

1 same level

2 lateral corner posterior to PPi orbital margin

17. postpineal plate anterior edge (new)

0 concave

1 flat

2 convex

18. postpineal plate (new)

0 without lateral flanges

1 with anterior flanges

19. Nuchal orbital edge (new)

0 absent

1 present

20. Nuchal plate shape #1 ([50]:text-fig. 68, character 27p, modified and split into 3 characters)

0: wider than long

1: longer than wide

2: as wide as long

21. Nuchal plate shape #2 ([50]:text-fig. 68, character 27p, split into 3 characters)

0: contact edge with La straight

1: contact edge with La convex

22. shape contact Nu/PaN (new)

This character is a possible alternative to [50] (character p in text.-fig. 68) and [37] (character 13)

0 straight

1 Nu convex

2 Nu concave

3 sigmoid

23. Nuchal plate ratio width intermesiolateral edge / width intero posterolateral edge (new)

0 <1

1 =1

2 >1

24. ratio length anterolateral margin (Nu-La) / length posterolateral margin (Nu-PaN) (new)

0 <1

1 =1

2 >1

25. Nuchal plate ratio anterolateral edge/posterolateral edge length (in [63]:char 10); [37]:char 13)

0

1 squarish Nu with convex anterior division of the lateral margin and short posterolateral corners

26. obtected nuchal area present on PaN ([37]:char 6)

0 absent

1 present

27. Shape of postmarginal plate (similar to [37]:char 15)

0 triangular

1 quadrangular

2 pentagonal (extra angle on edge with La)

28. Ratio Length/Width of postmarginal plate (new)

0 <1

1 =1

2 >1

29. SM plate shape ([37]:char 10 pro parte; [50]:text-fig. 68, character 27g)

0: long and shallow

1: short and deep

30. AMD width: broadest at lateral angles in adults ([50]:text-fig. 68, character 27b); [37]:char 1)

0: no

1: yes

31. lateral angles of AMD appear early in ontogeny ([50]:text-fig. 68, character 27c; also in [37]:char 2)

0: no

1: yes

32. Median Dorsal plates crest ([50]:text-fig. 68, character 27f); [37]:char 17))

0: none

1: minor

2: well marked

33. margin AMD-ADL (new)

0 straight

1 sigmoid / sinuous

34. margin AMD-MxL (new)

0 straight

1 sigmoid / sinuous

35. MxL broadest at its dorsal corners in adults ([50]:text-fig. 68, character 27b)

0: no

1: yes

36. crista transversalis interna anterior transversally oriented on ventral lamina of AVL ([50]:text-fig. 68, character 27n; [37]:char 11)

0: no

1: yes

37. ventrolateral ridge makes a keel ([37]:char 20)

0 no

1 yes

38. axillary foramen shape ([50]:text-fig. 68, character 27k; [37]:character 4)

0: higher than long

1: longer than high

2: as long as high

39. contact Cv1, Cv2, Mm1, Mm2 in pectoral fin ([50]:text-fig. 68, character 27h; [37]:char 3)

0 all contact in one point

1 Mm1+Cv2 separate Cv1 from Mm2

2 Cv1 and Mm2 contact

40. Cd5 on distal segment ([37]:char 14)

0 absent

1 present

41. middle pit-line long ([37]:char 9 pro parte) (

0 absent

1 present

42. supra-occipital sensory groove long ([37]:char 9 pro parte)

0 absent

1 present

43. branch of infraorbital sensory line diverging on PrL parallel to the rostral margin of the head-shield (also in [37]:char 12)

0 no

1 yes

44. main lateral line canal/groove reaches the posterior margin of the MxL plate ([37]:char 16)

0 no

1 yes

45. Long branch of the infraorbital sensory groove/canal diverging on PrL (also in [37]:char 19)

0 no

1 yes

46. Junction of rostral ifc in midline on PRM (new)

0 straight

1 light posterior indet

2 very deep posterior indent

47. Semi-circular groove on La (new)

0 reduced

1 developed

48. central sensory groove shape (new)

0 straight

1 curved

49. central sensory groove on Nu (new)

0 only Nu

1 extends on La

50. central sensory groove (new; slightly different from character 42 about extension of csc on Nu only or onto La)

0 directed to semicircular groove

1 directed to La/PaNu/Nu point

# Phylogenetic analyses

## Different outgroups

|  | **complete taxa + char** | **complete taxa** | **OG *Remigolepis*** | **OG *Grossilepis*** | **OG *niushoushanensis*** | **OG *shaokuanensis*** | **OG *askinae*** | **OG *askinae* (-*virginiensis)*** |
| --- | --- | --- | --- | --- | --- | --- | --- | --- |
| **total char** | 59 | 33 | 50 | 50 | 50 | 50 | 50 | 50 |
| **total taxa** | 54 | 54 | 47 | 46 | 45 | 45 | 45 | 44 |
| **n_MPT_** | 10000 (maxtrees) | 10000 (maxtrees) | 50 | 50 | 50 | 54 | 54 | 96 |
| **L_MPT_** | 248 | 76 | 228 | 255 | 217 | 217 | 217 | 210 |
| **CI_MPT_** | 0.327 | 0.526 | 0.351 | 0.323 | 0.327 | 0.327 | 0.327 | 0.333 |
| **RI_MPT_** | 0.512 | 0.747 | 0.502 | 0.513 | 0.507 | 0.507 | 0.507 | 0.509 |
| **L_50%_** | 331 | 125 | 266 | 253 | 247 | 247 | 247 | 251 |
| **CI_50%_** | 0.245 | 0.32 | 0.282 | 0.292 | 0.287 | 0.287 | 0.287 | 0.279 |
| **RI_50%_** | 0.27 | 0.401 | 0.416 | 0.423 | 0.405 | 0.405 | 0.405 | 0.665 |

Table C. Phylogenetic indices obtained using various outgroups and data sets. n_MPT_, number of most parsimonious trees; L_MPT_, length of each most parsimonious tree; CI_MPT_, consistency index of each most parsimonious tree ; RI_MPT_, retention index of each most parsimonious tree ; L_50%_, length of the majority rule consensus tree ; CI_50%_, consistency index of the majority rule consensus tree ; RI*50%*, retention index of the majority rule consensus tree.

### all characters and all taxa (but Dianolepis)


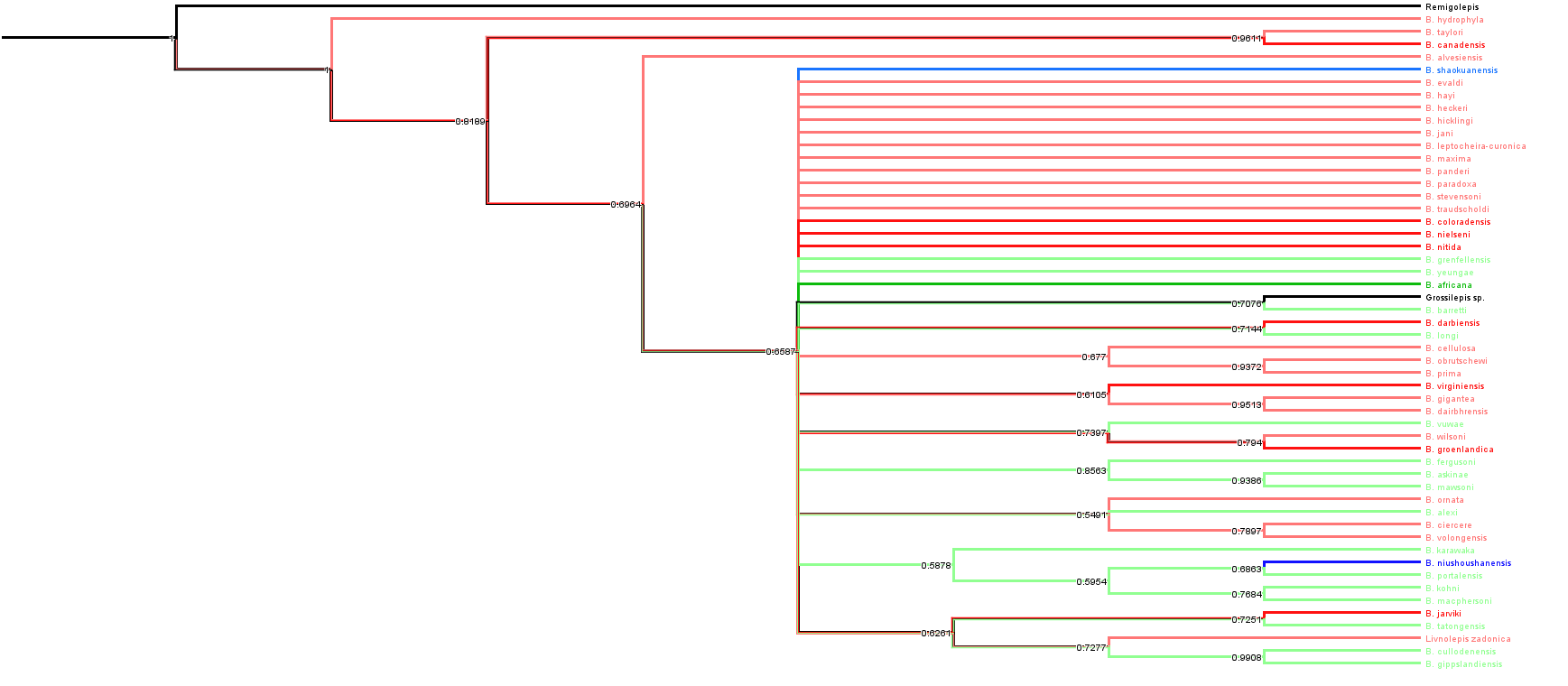


S9 Fig. Majority rule consensus tree of the phylogeny of the genus Bothriolepis using the complete data set (59 characters * 54 taxa). Colours indicate palaeogeographic affinities of taxa (red Laurussia, green Gondwana, blue China; black is outgroup).

### all taxa (but Dianolepis)


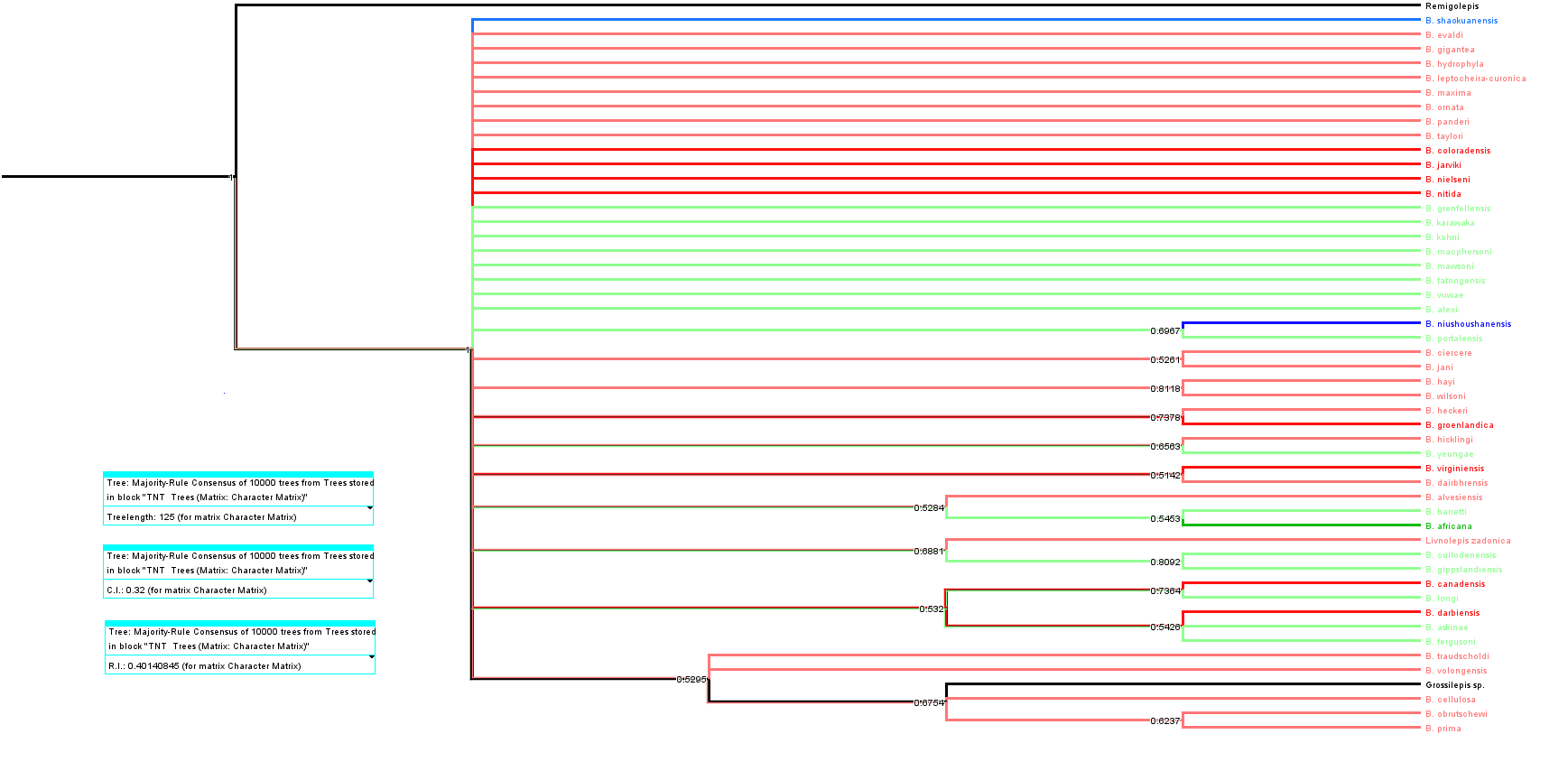


S10 Fig. Majority rule consensus tree of the phylogeny of the genus *Bothriolepis* using the complete data set (33 characters * 54 taxa). Colours indicate palaeogeographic affinities of taxa (red Laurussia, green Gondwana, blue China; black is outgroup).

| **n_MPT_** | 10000 (maxtrees) |
| --- | --- |
| **L_MPT_** | 76 |
| **CI_MPT_** | 0,526 |
| **RI_MPT_** | 0,747 |
| **L_50%_** | 125 |
| **CI_50%_** | 0,32 |
| **RI_50%_** | 0,401 |

### Outgroup = Remigolepis + Grossilepis

50 characters (bad ones removed) *47 taxa (bad ones removed)

LMPT=228

nMPT=50

CIMPT=0.351

RIMPT=0.502


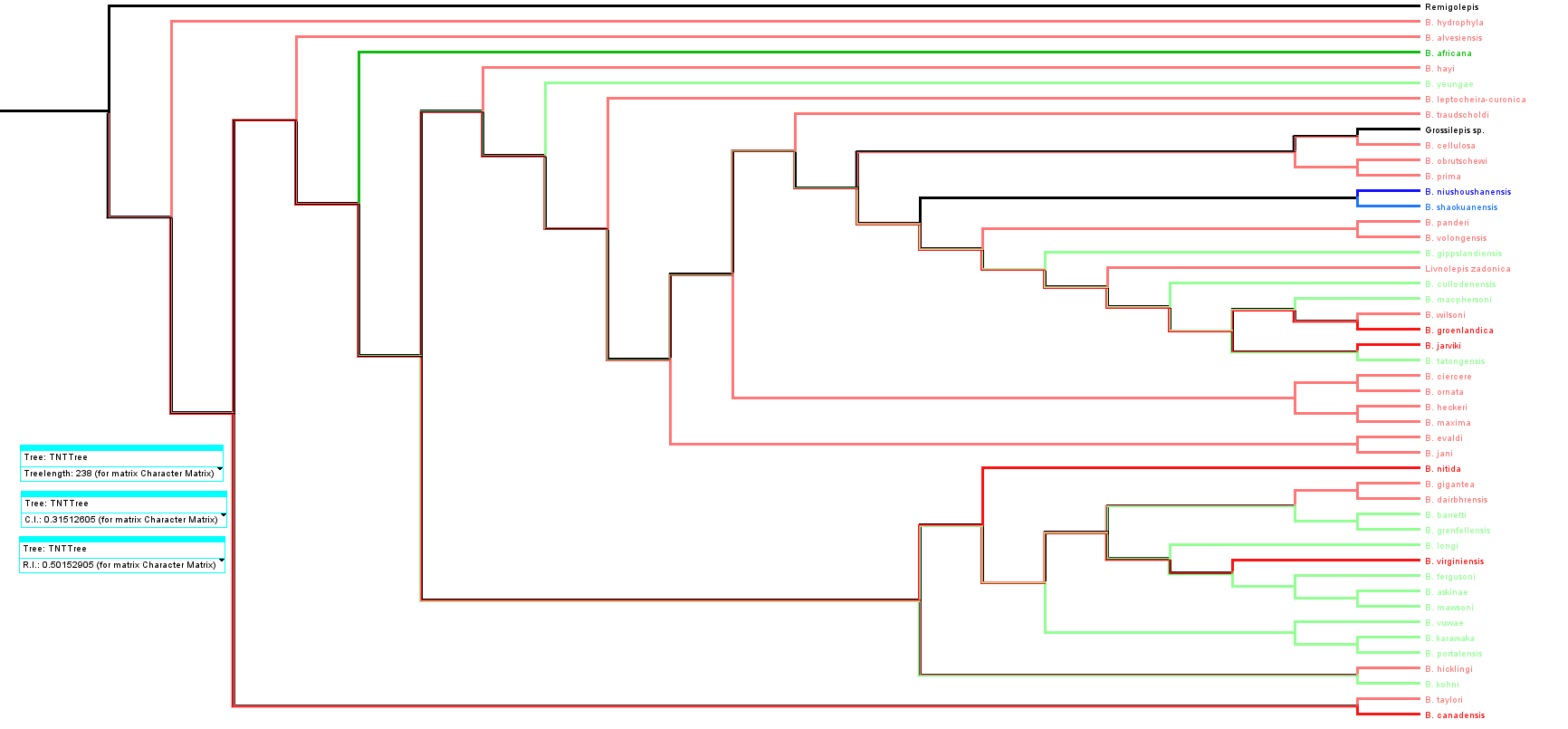


S11 Fig. Tree #1 (out of 50) showing the phylogeny of the genus *Bothriolepis* using the reduced data set (50 characters * 47 taxa). Colours indicate palaeogeographic affinities of taxa (red Laurussia, green Gondwana, blue China; black is outgroup).

-------------------

50% maj rule:

L50%=266

CI50%=0.282

RI50%=0.416


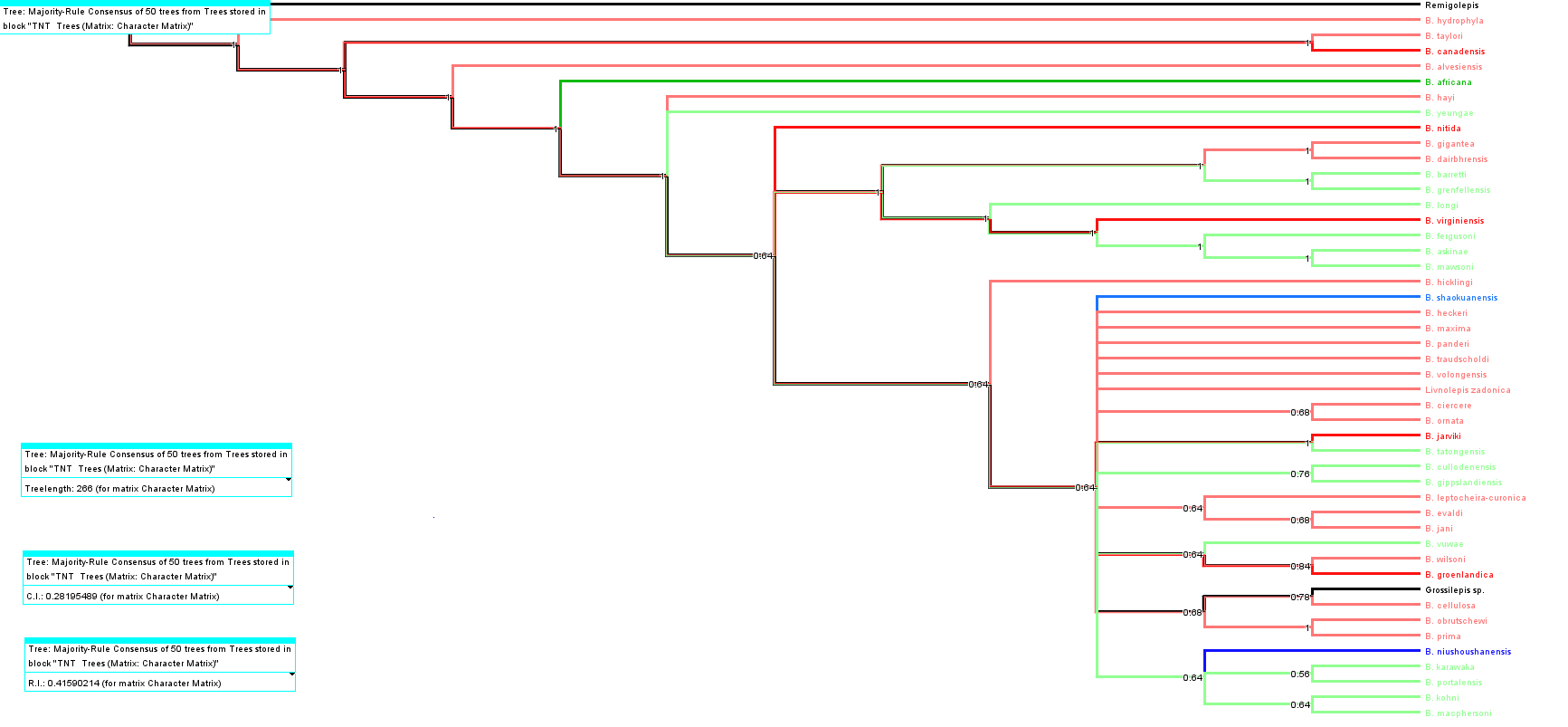


S12 Fig. Majority rule consensus tree of the phylogeny of the genus *Bothriolepis* using the reduced data set (50 characters * 47 taxa). Colours indicate palaeogeographic affinities of taxa (red Laurussia, green Gondwana, blue China; black is outgroup).

### Outgroup = Grossilepis

50 characters (bad ones removed) *46 taxa (bad ones removed)

LMPT=225

nMPT=50

CIMPT=0.323

RIMPT=0.513


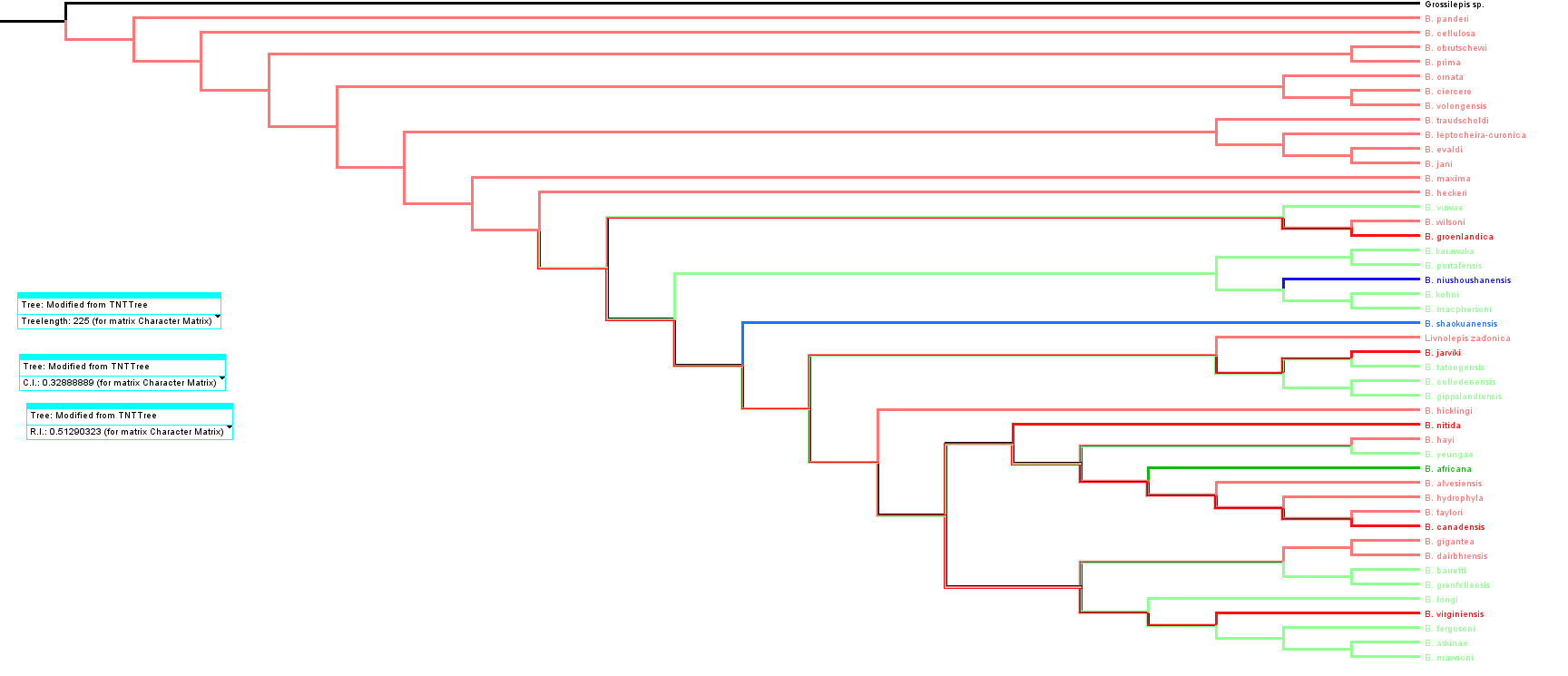


S13 Fig. Tree #1 (out of 50) showing the phylogeny of the genus *Bothriolepis* using the reduced data set (50 characters * 46 taxa). Colours indicate palaeogeographic affinities of taxa (red Laurussia, green Gondwana, blue China; black is outgroup).

---------------------

50% maj rule:

L50%=253

CI50%=0.292

RI50%=0.423


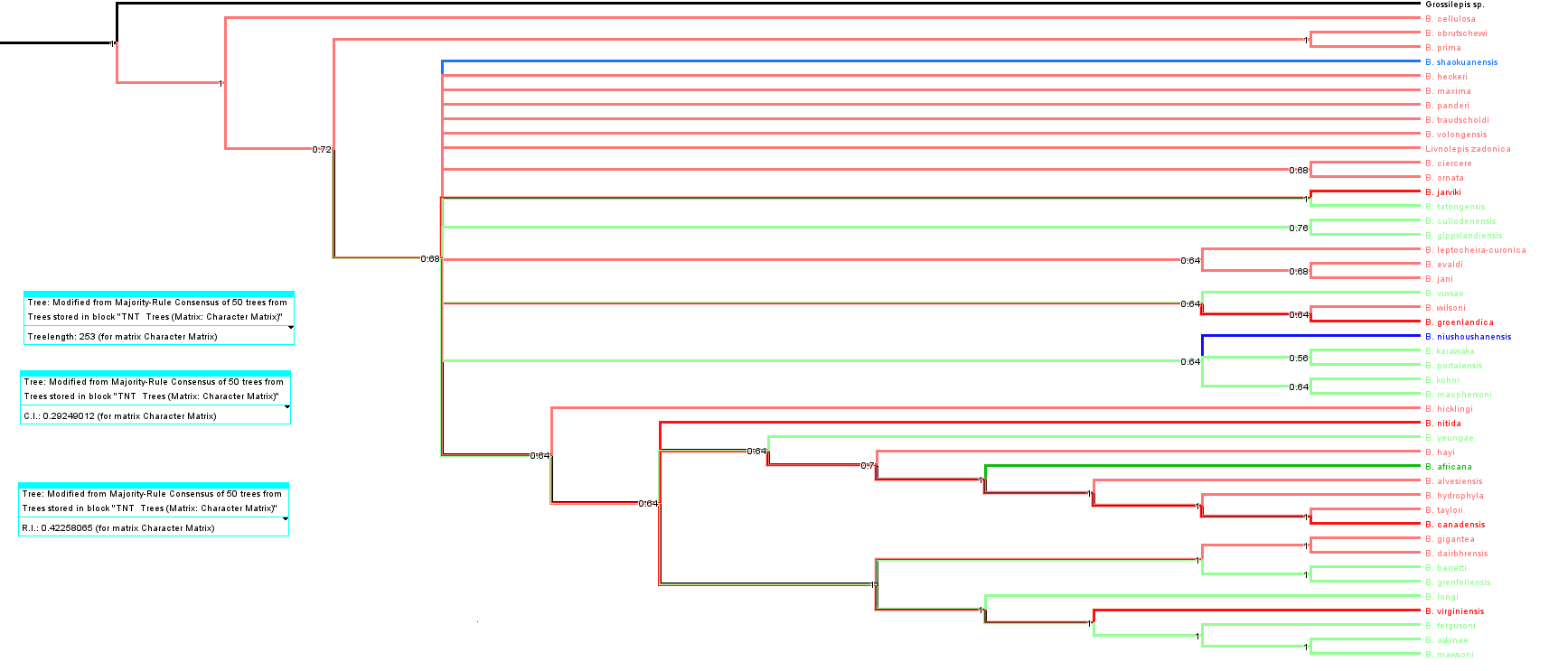


S14 Fig. Majority rule consensus tree of the phylogeny of the genus *Bothriolepis* using the reduced data set (50 characters * 46 taxa). Colours indicate palaeogeographic affinities of taxa (red Laurussia, green Gondwana, blue China; black is outgroup).

### Outgroup = B. niushoushanensis

50 characters (bad ones removed) *45 taxa (bad ones removed)

LMPT=217

nMPT=50

CIMPT=0.327

RIMPT=0.507


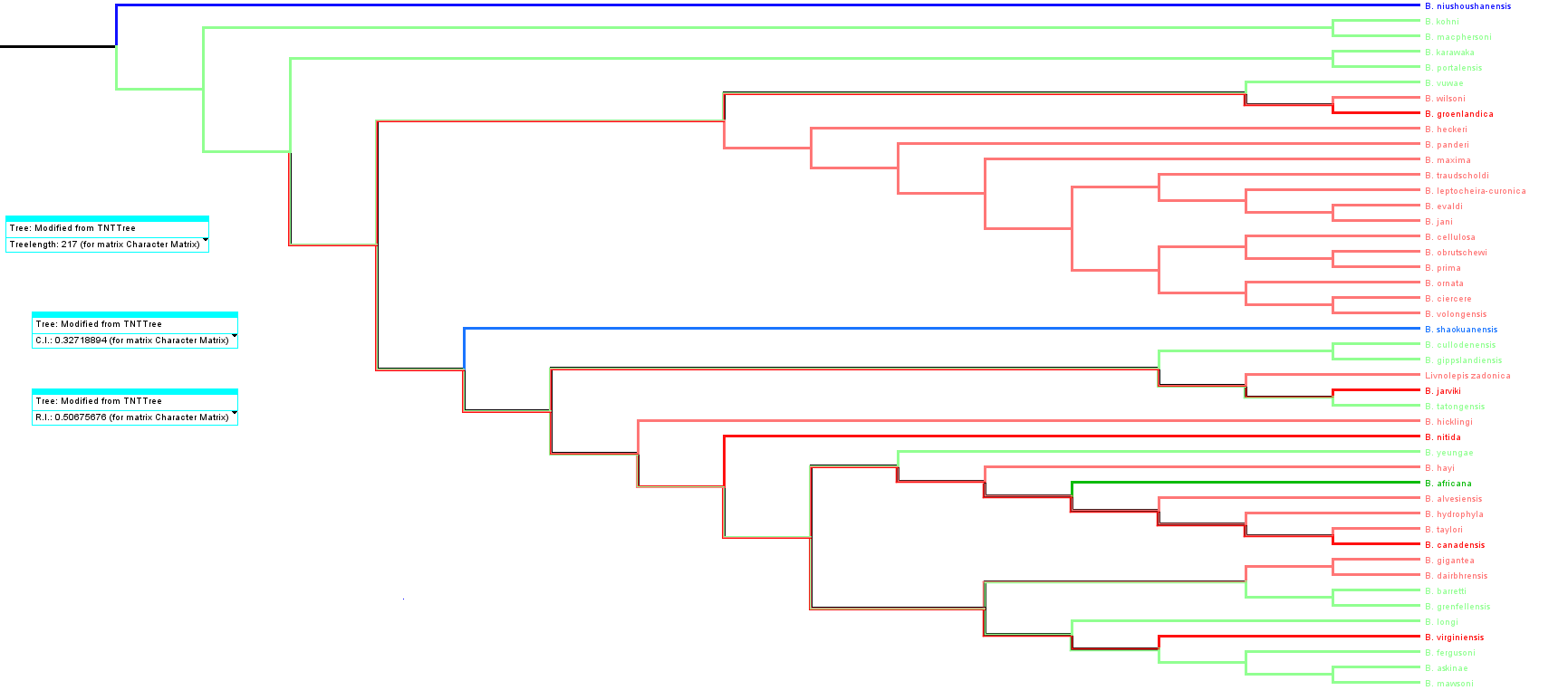


S15 Fig. Tree #1 (out of 50) showing the phylogeny of the genus *Bothriolepis* using the reduced data set (50 characters * 45 taxa). Colours indicate palaeogeographic affinities of taxa (red Laurussia, green Gondwana, blue China).

---------

50% maj rule:

L50%=247

CI50%=0.287

RI50%=0.405


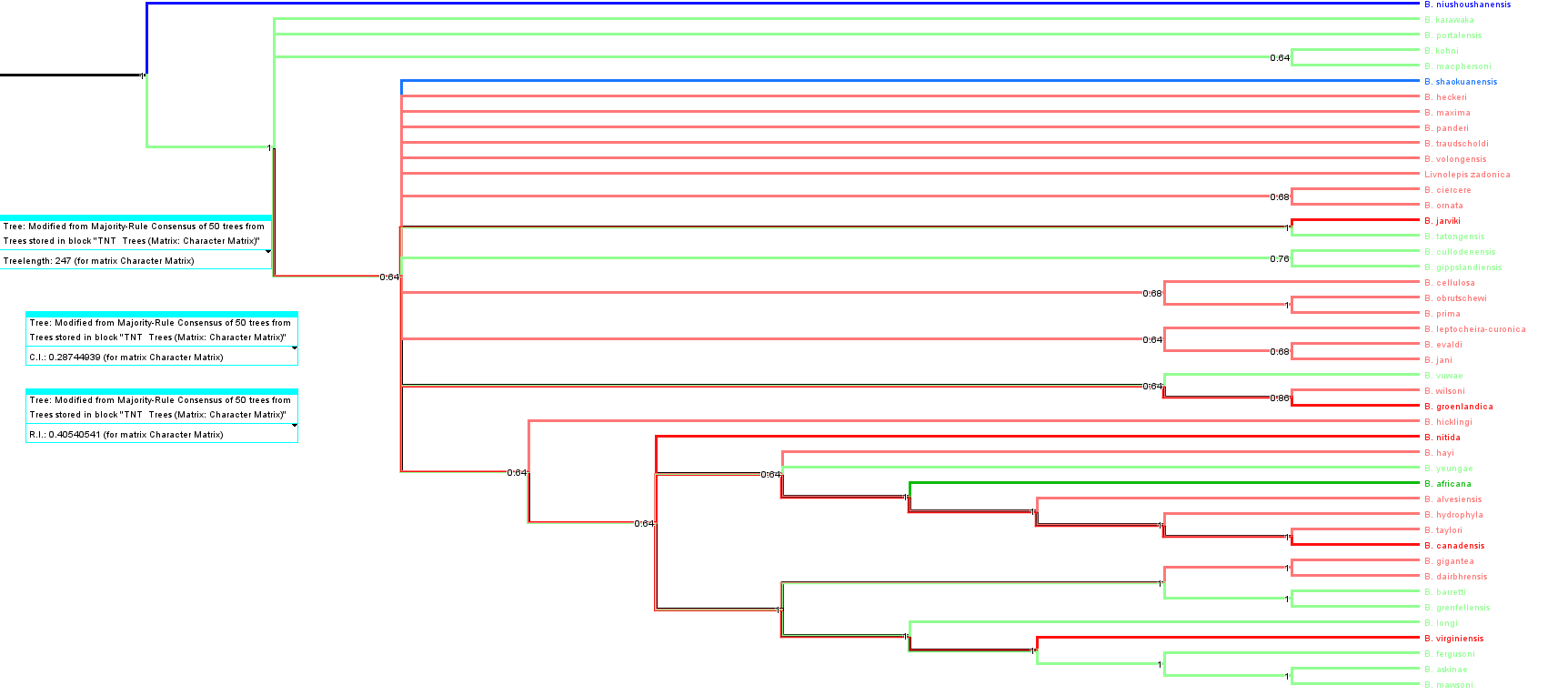


S16 Fig. Majority rule consensus tree of the phylogeny of the genus *Bothriolepis* using the reduced data set (50 characters * 45 taxa). Colours indicate palaeogeographic affinities of taxa (red Laurussia, green Gondwana, blue China).

### Outgroup = B. shaokuanensis

50 characters (bad ones removed) *45 taxa (bad ones removed)

LMPT=217

nMPT=54

CIMPT=0.327

RIMPT=0.507


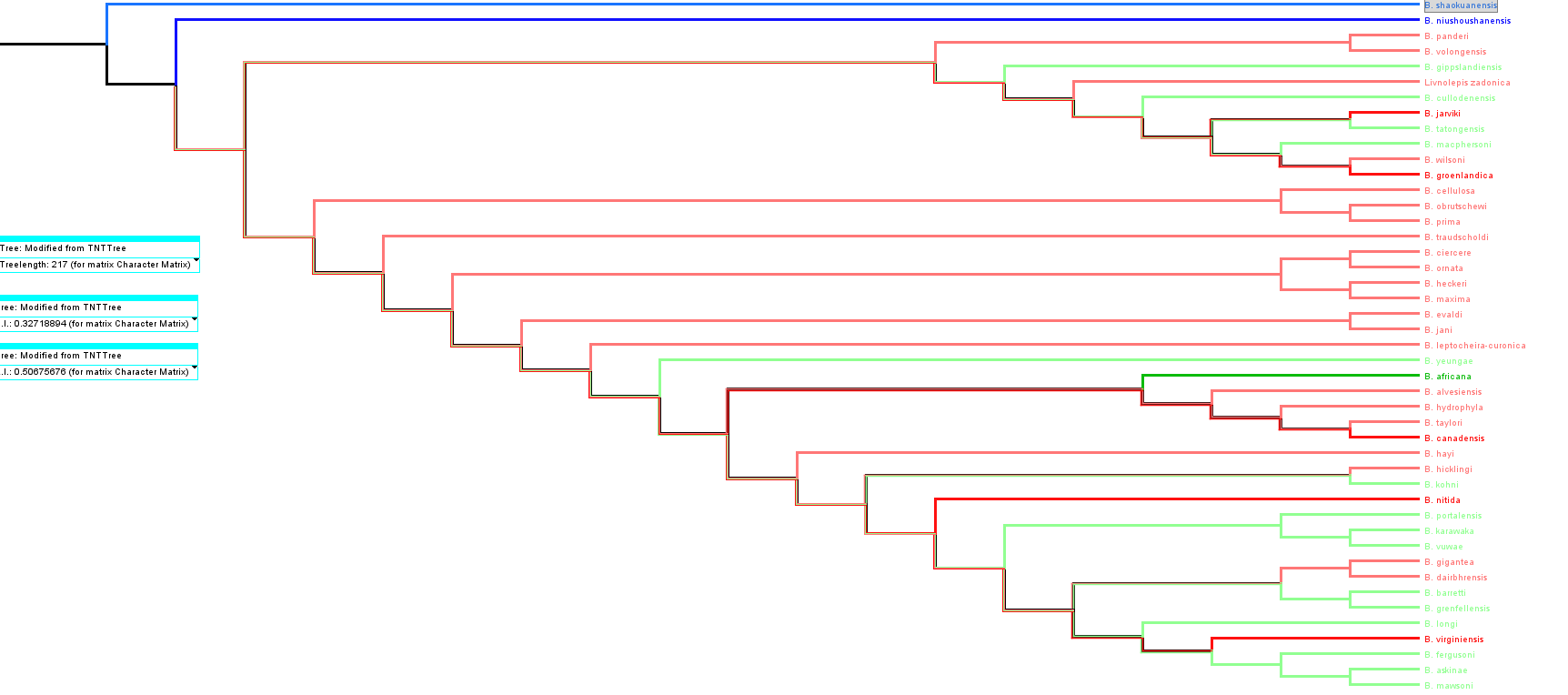


S17 Fig. Tree #1 (out of 50) showing the phylogeny of the genus *Bothriolepis* using the reduced data set (50 characters * 45 taxa). Colours indicate palaeogeographic affinities of taxa (red Laurussia, green Gondwana, blue China).

----------------

50% maj rule:

L50%=247

CI50%=0.287

RI50%=0.405


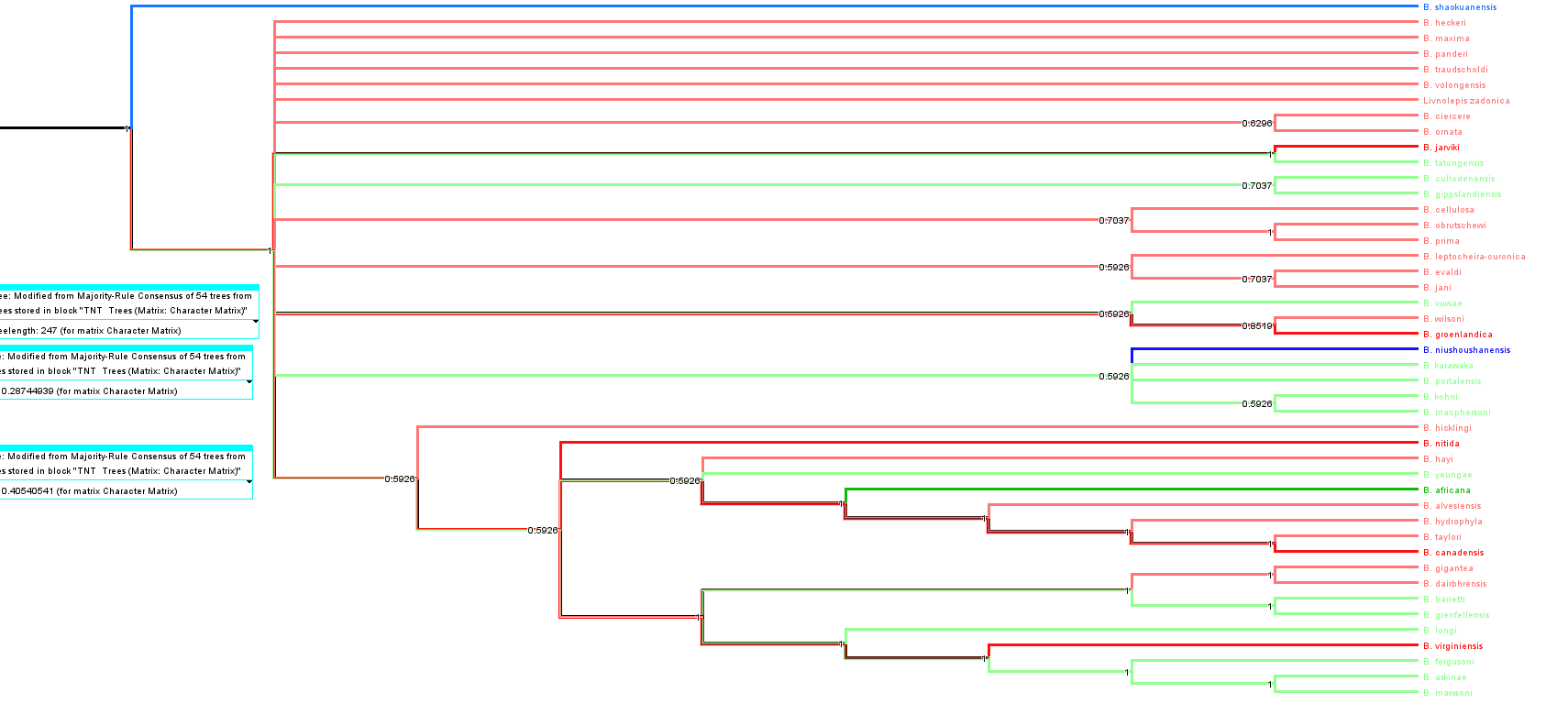


S18 Fig. Majority rule consensus tree of the phylogeny of the genus *Bothriolepis* using the reduced data set (50 characters * 45 taxa). Colours indicate palaeogeographic affinities of taxa (red Laurussia, green Gondwana, blue China).

### Outgroup = B. askinae

50 characters (bad ones removed) *45 taxa (bad ones removed)

LMPT=217

nMPT=54

CIMPT=0.327

RIMPT=0.507


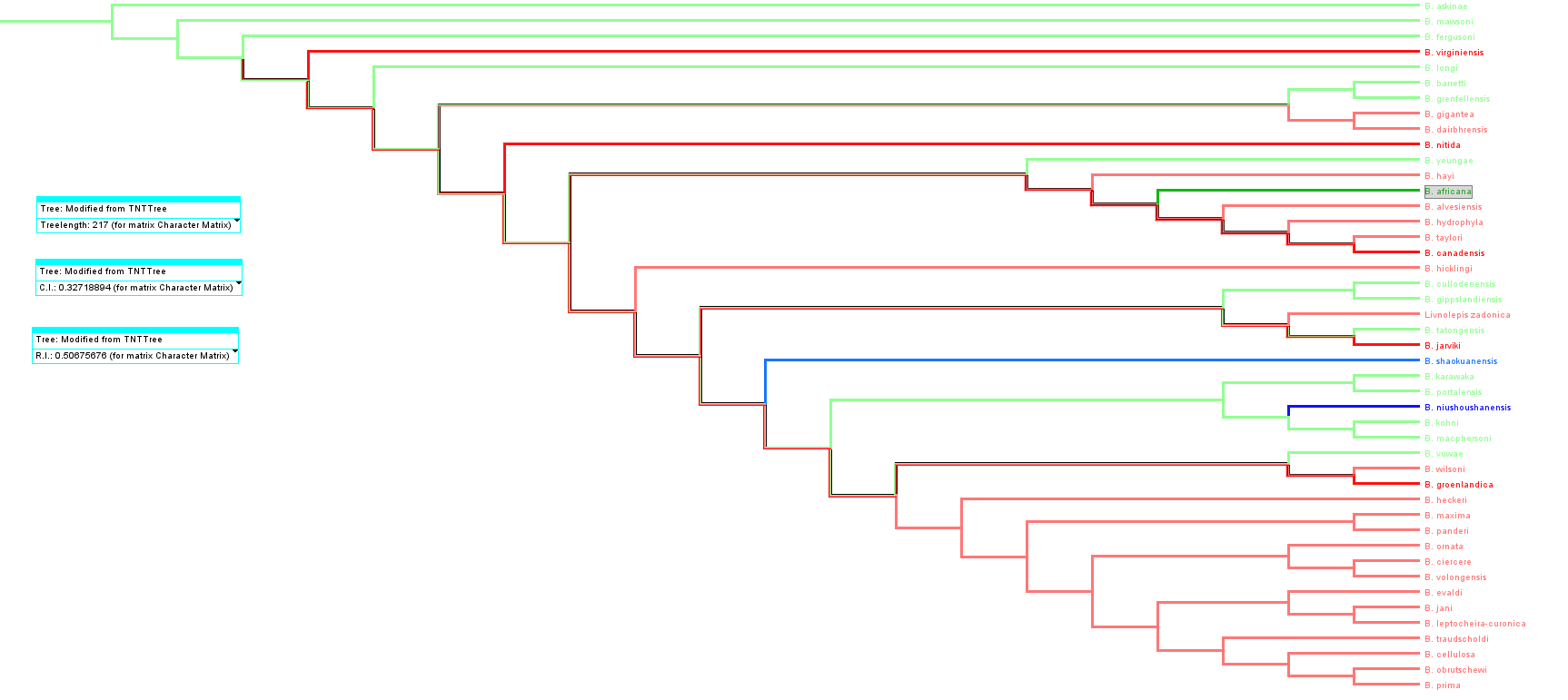


S19 Fig. Tree #1 (out of 50) showing the phylogeny of the genus *Bothriolepis* using the reduced data set (50 characters * 45 taxa). Colours indicate palaeogeographic affinities of taxa (red Laurussia, green Gondwana, blue China).

-------------------


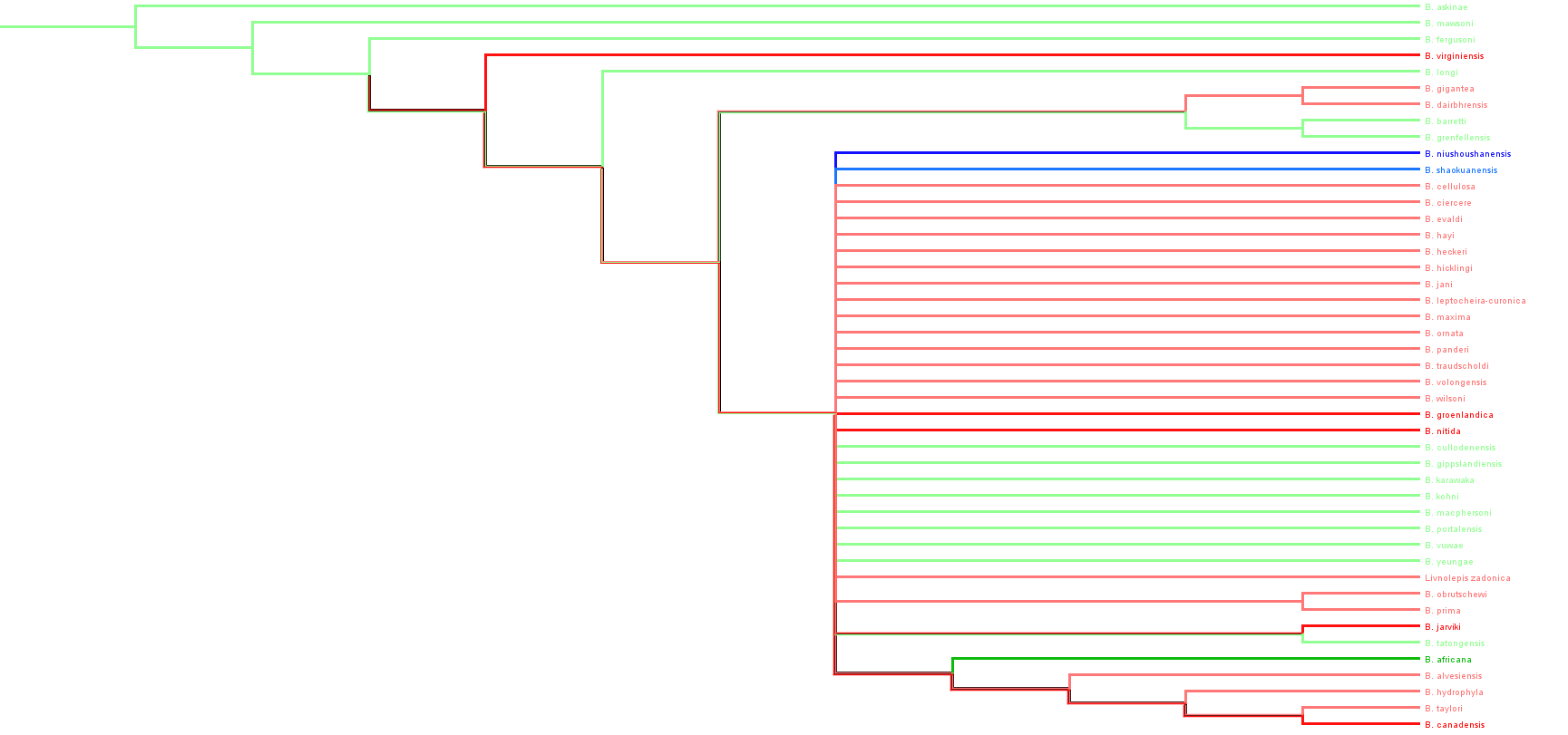


S20 Fig. Strict consensus tree of the phylogeny of the genus *Bothriolepis* using the reduced data set (50 characters * 45 taxa). Colours indicate palaeogeographic affinities of taxa (red Laurussia, green Gondwana, blue China).

L_CS_ = 308 steps,

CI_CS_ = 0.231,

RI_CS_ = 0.199

----------

50% maj rule:

L50%=247

CI50%=0.287

RI50%=0.405


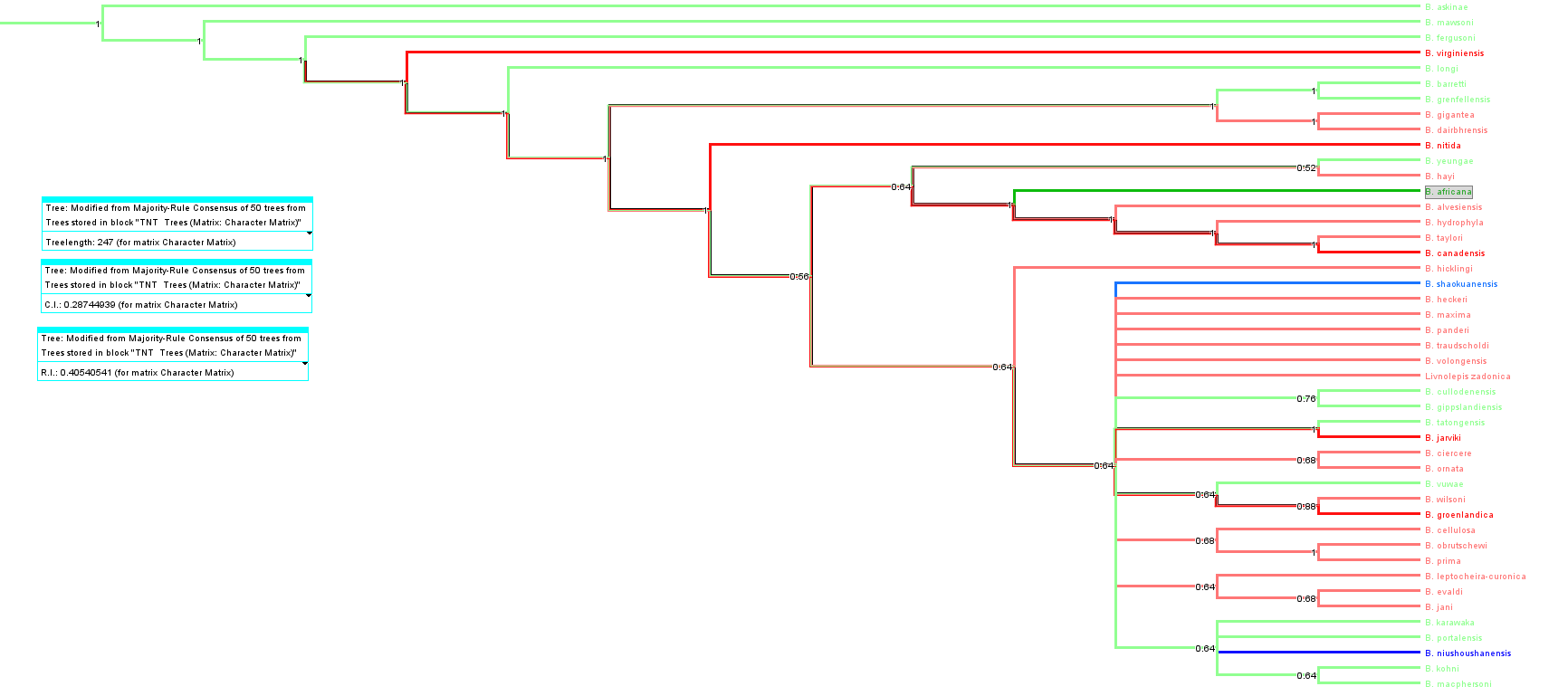


S21 Fig. Majority rule consensus tree of the phylogeny of the genus *Bothriolepis* using the reduced data set (50 characters * 45 taxa). Colours indicate palaeogeographic affinities of taxa (red Laurussia, green Gondwana, blue China).

------------------


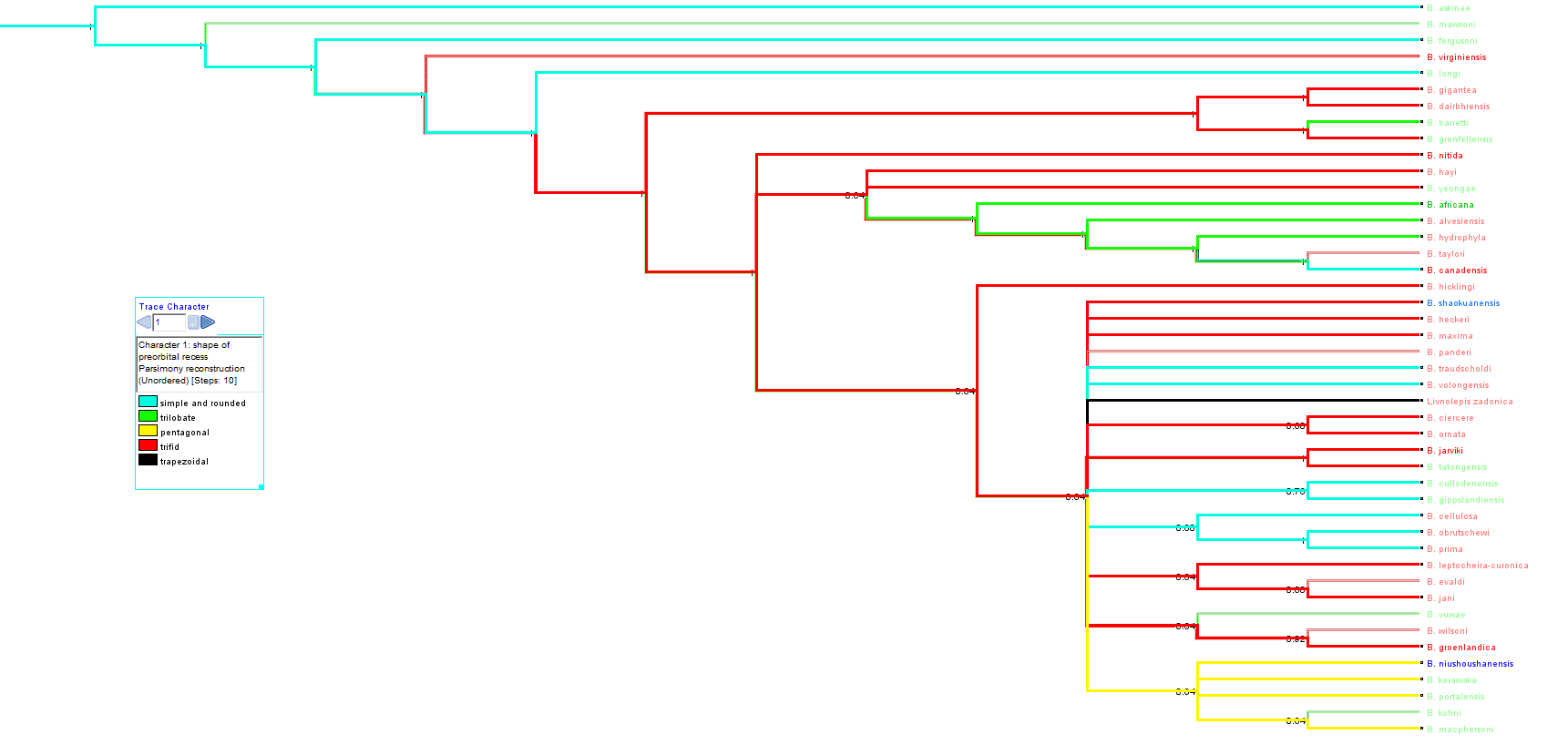


S22 Fig. Majority rule consensus tree of the phylogeny of the genus *Bothriolepis* using the reduced data set (50 characters * 45 taxa). Colours indicate distribution of preorbital recess shape (character #1; blue simple and rounded, green trilobate, yellow pentagonal, red trifid, black trapezoidal).

### Outgroup = B. askinae (B. virginiensis removed)

50 characters (bad ones removed) *44 taxa (bad ones removed)

LMPT=210

nMPT=96

CIMPT=0.333

RIMPT=0.509


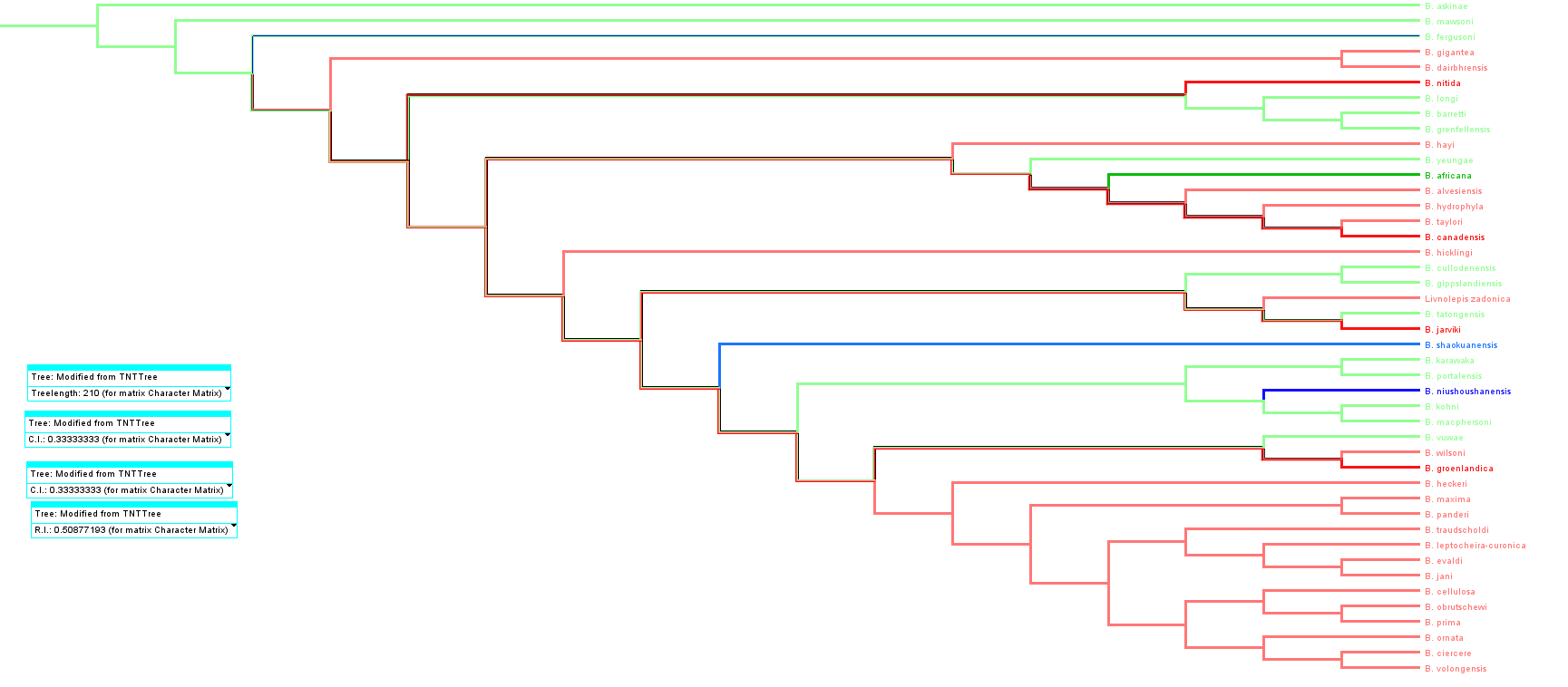


S23 Fig. Tree #1 (out of 50) showing the phylogeny of the genus *Bothriolepis* using the reduced data set (50 characters * 45 taxa). Colours indicate palaeogeographic affinities of taxa (red Laurussia, green Gondwana, blue China).

S24 Fig. Strict consensus tree of the phylogeny of the genus *Bothriolepis* using the reduced data set (50 characters * 45 taxa). Colours indicate palaeogeographic affinities of taxa (red Laurussia, green Gondwana, blue China).

L_CS_ = 253 steps,

CI_CS_ = 0.277,

RI_CS_ = 0.358

------------------------

50% maj rule:

L50%=251

CI50%=0.279

RI50%=0.665


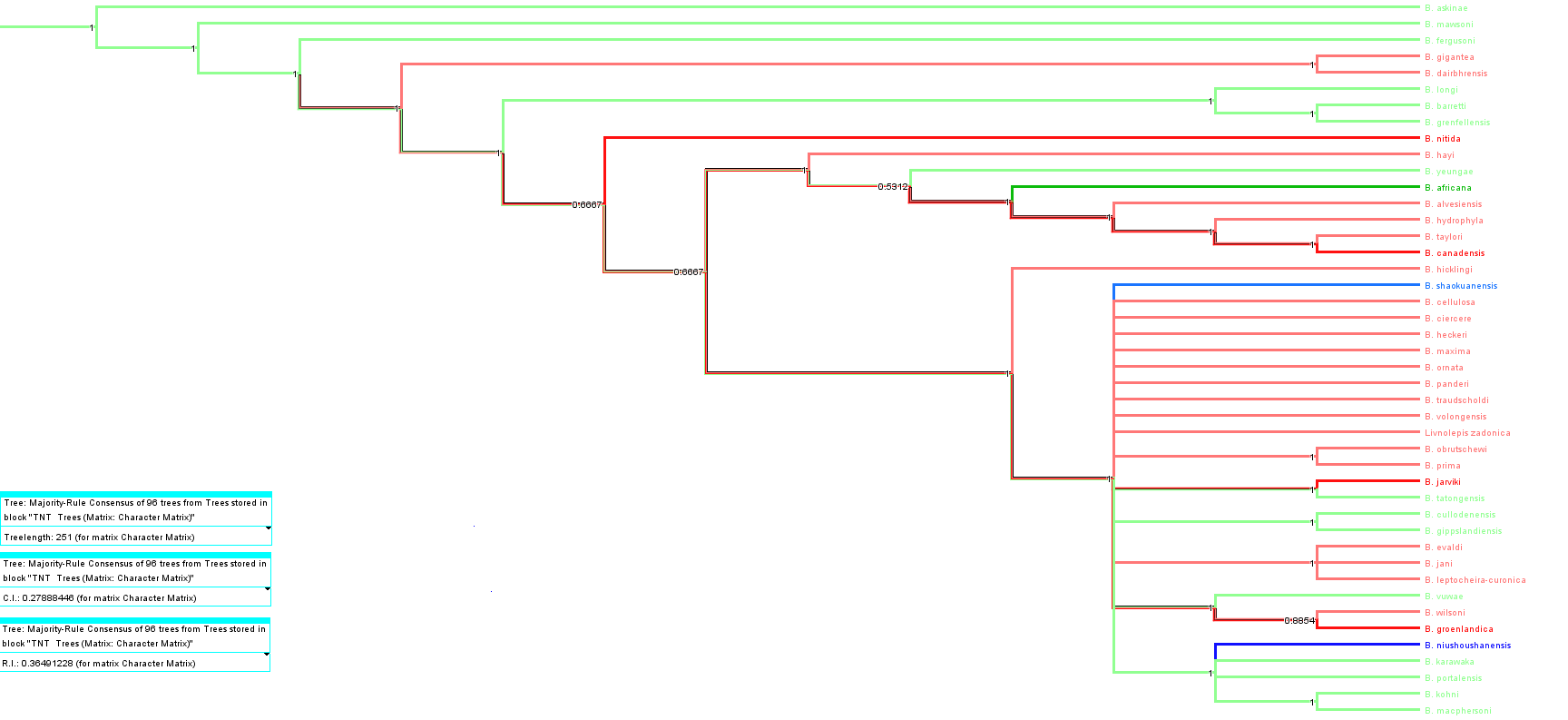


S25 Fig. Majority rule consensus tree of the phylogeny of the genus *Bothriolepis* using the reduced data set (50 characters * 45 taxa). Colours indicate palaeogeographic affinities of taxa (red Laurussia, green Gondwana, blue China).

------------------------


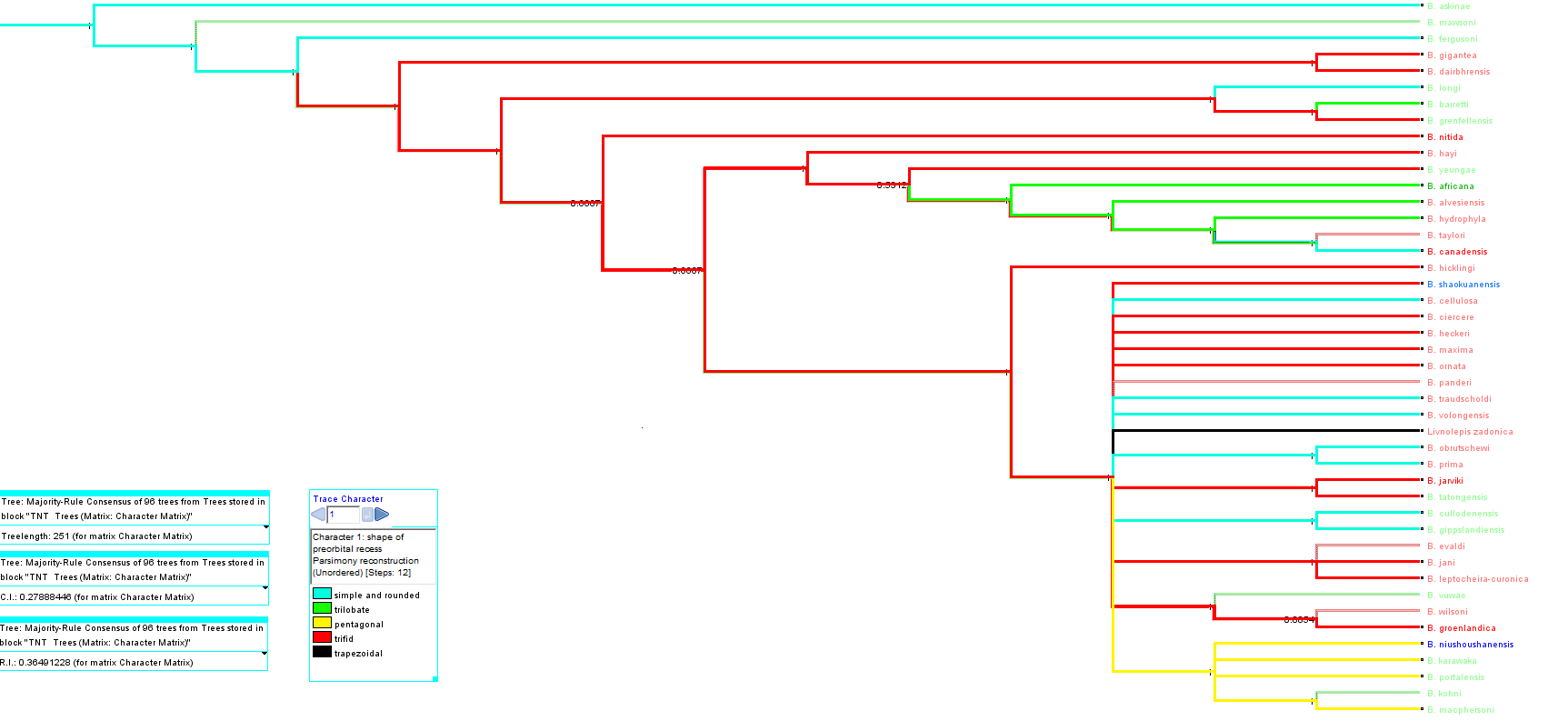


S26 Fig. Majority rule consensus tree of the phylogeny of the genus *Bothriolepis* using the reduced data set (50 characters * 45 taxa). Colours indicate distribution of preorbital recess shape (character #1; blue simple and rounded, green trilobate, yellow pentagonal, red trifid, black trapezoidal).

### Outgroup = Remigolepis + Grossilepis + Dianolepis

Another set of analyses incorporating *Dianolepis* as outgroup is attempted, as this genus is supposedly close to *Bothriolepis* but not as close at *Grossilepis* (see for example [94]). The results of these analyses prove unreliable for several reasons. Firstly, in case of several outgroups, one of them at least is resolved as part of the ingroup; Secondly, most often the most basal taxa are neither Gondwana nor Chinese but Euramerican, contradicting the current fossil record succession. Thirdly, the resulting topologies of the majority rule consensus trees present impressive polytomies basally.

#### Complete data set (59 characters, 55 taxa)

n=10000; L=258; CI=0.326 ; RI=0.508; L_50_=280; CI_50_=0.300; RI_50_=0.446.

The majority rule consensus tree is poorly resolved and shows a big basal polytomy. *Dianolepis* is resolved as sister group to Chinese clade, *Grossilepis* is resolved as sister group to B. panderi; both *Grossilepis* and *Dianolepis* are very apical in the tree.


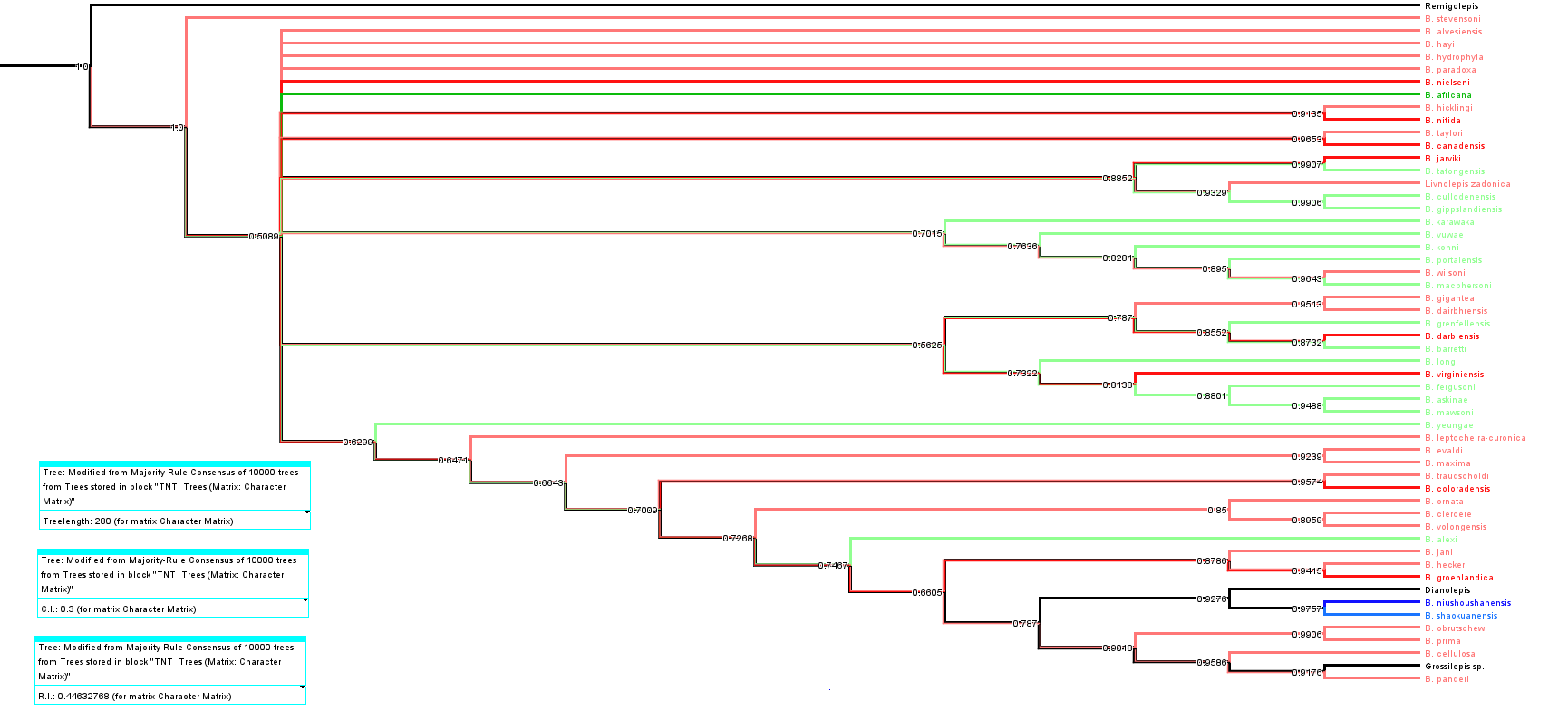


S27 Fig. Majority rule consensus tree of the phylogeny of the genus *Bothriolepis*, including *Remigolepis*, *Grossilepis* and *Dianolepis*, using the reduced data set (59 characters * 55 taxa). Colours indicate palaeogeographic affinities of taxa (red Laurussia, green Gondwana, blue China; black is outgroup).

#### Reduced data set (59 characters, 48 taxa)

n=8; L=257; CI=0.327; RI=0.503; L_50_=258; CI_50_=0.326; RI_50_=0.500.

The majority rule consensus tree is well resolved, but the most basal branching taxa are Euramerican species. *Dianolepis* and *Grossilepis* are again very apical, but relationships are very different from the previous topology. *Dianolepis* is sister group with *B. groenlandica*, *Grossilepis* is sister group *with B. cellulosa*.


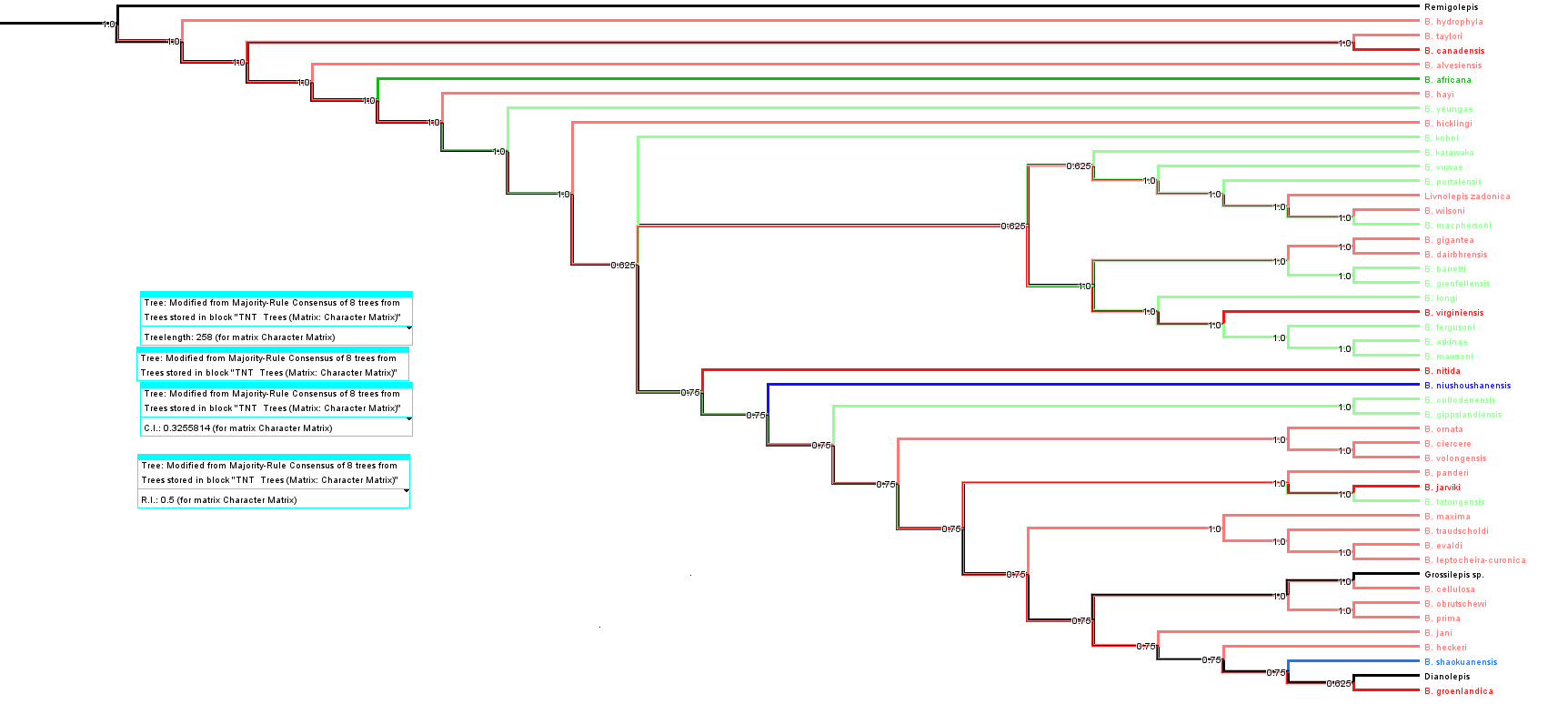


S28. Fig. Majority rule consensus tree of the phylogeny of the genus *Bothriolepis*, including *Remigolepis*, *Grossilepis* and *Dianolepis*, using the reduced data set (59 characters * 48 taxa). Colours indicate palaeogeographic affinities of taxa (red Laurussia, green Gondwana, blue China; black is outgroup).

#### Reduced data set (49 characters, 48 taxa)

n=534; L=246; CI=0.313; RI=0.499; L_50_=300; CI_50_=0.256; RI_50_=0.338.

The majority rule consensus tree shows a basal polytomy and a few more apical ones, but the most basal branches are Gondwanan ; *Dianolepis* is resolved as sister group for *B. barretti*, and *Grossilepis* as sister group for *B. cellulosa*.


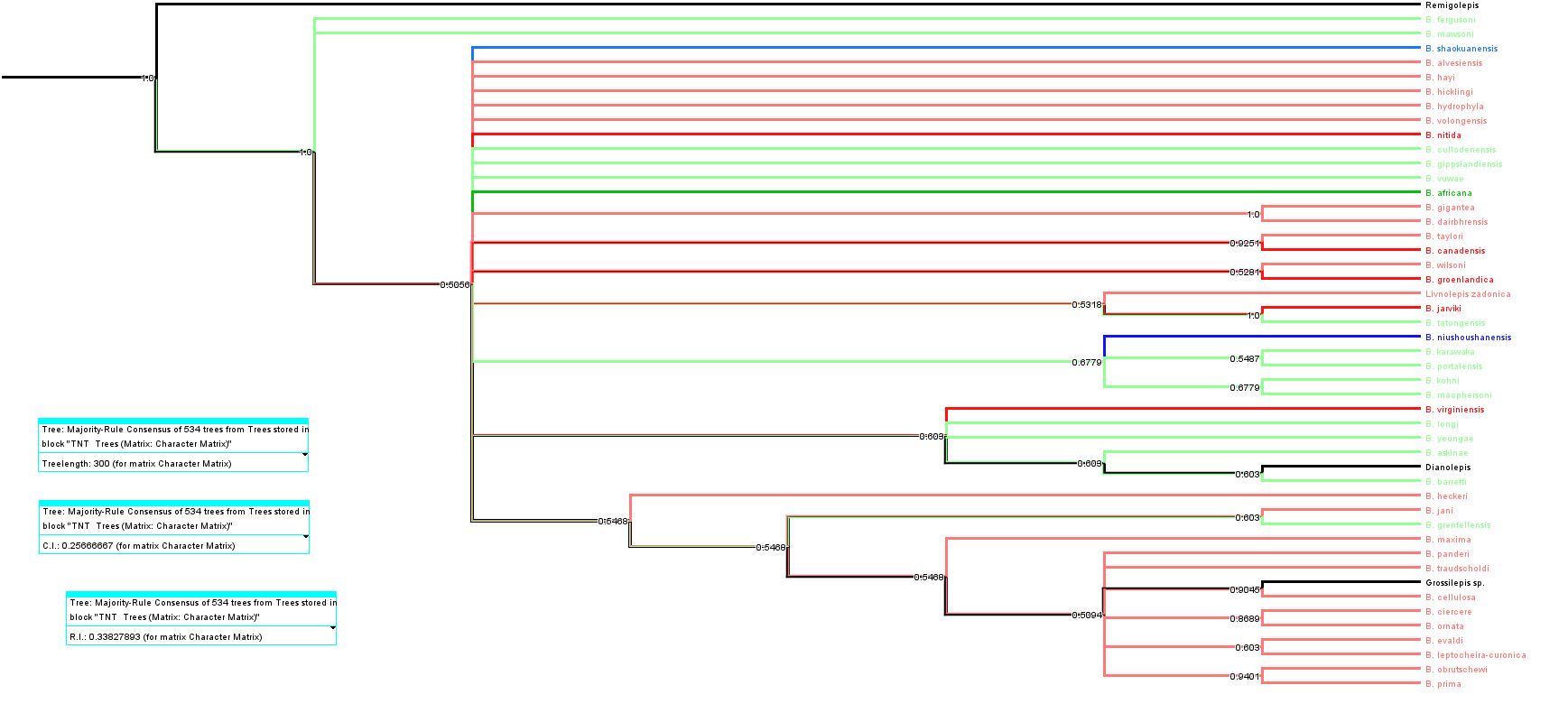


S29 Fig. Majority rule consensus tree of the phylogeny of the genus *Bothriolepis*, including *Remigolepis*, *Grossilepis* and *Dianolepis*, using the reduced data set (49 characters * 48 taxa). Colours indicate palaeogeographic affinities of taxa (red Laurussia, green Gondwana, blue China; black is outgroup).

### Outgroup = Grossilepis + Dianolepis

n=332; L=233; CI=0.330; RI=0.511; L_50_=286; CI_50_=0.269; RI_50_=0.345.

The majority rule consensus tree shows a basal polytomy, and the most basal branch is Euramerican; *Dianolepis* is unresolved in a basal polytomy; the Chinese species form a clade branching from this polytomy.


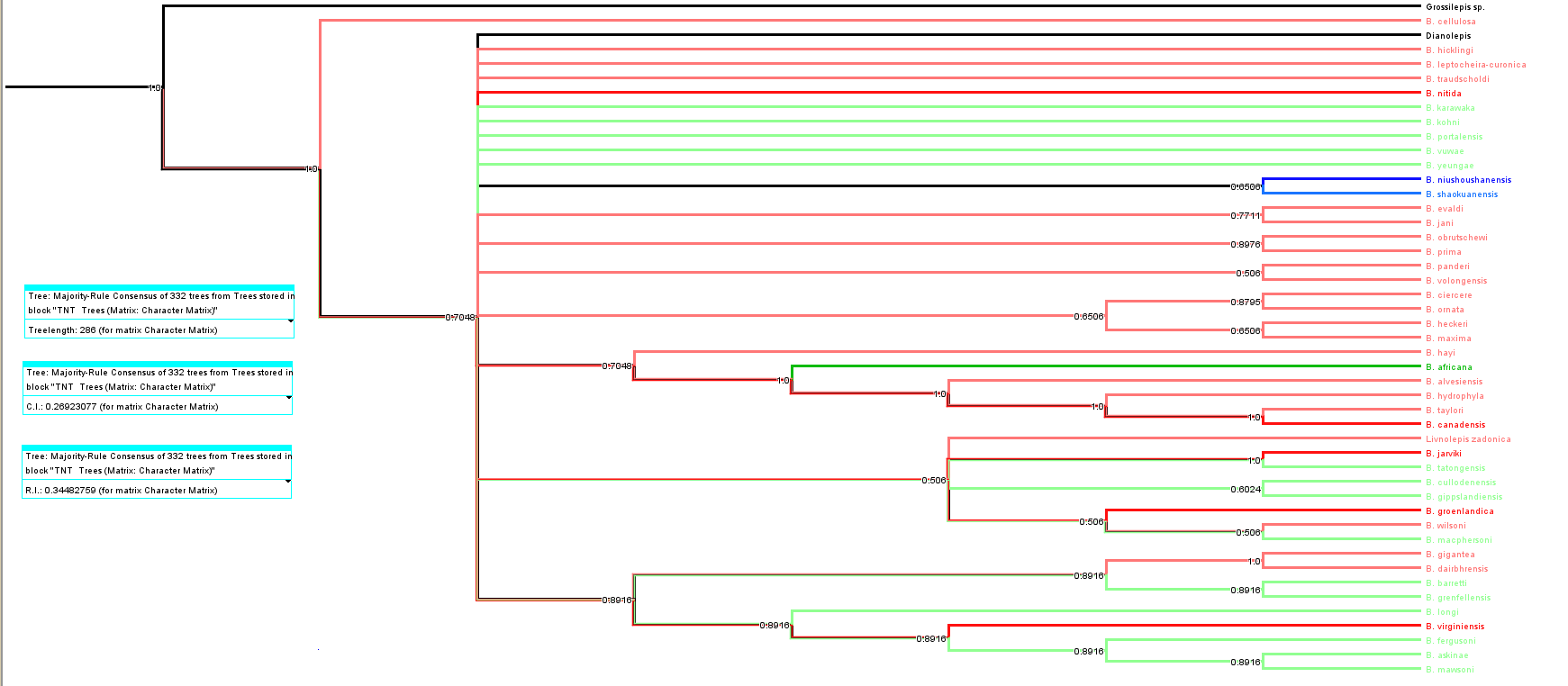


S30 Fig. Majority rule consensus tree of the phylogeny of the genus *Bothriolepis*, including *Grossilepis* and *Dianolepis*, using the reduced data set (49 characters * 47 taxa). Colours indicate palaeogeographic affinities of taxa (red Laurussia, green Gondwana, blue China; black is outgroup).

### Outgroup = Dianolepis + Grossilepis

n=262; L=233; CI=0.330; RI=0.511; L_50_=299; CI_50_=0.258; RI_50_=0.304.

The majority rule consensus tree shows a basal polytomy, and no basal branch can be identified; *Grossilepis* is resolved as sister group for *B. cellulosa*, and the clade branches from the basal polytomy; the Chinese species are scattered.


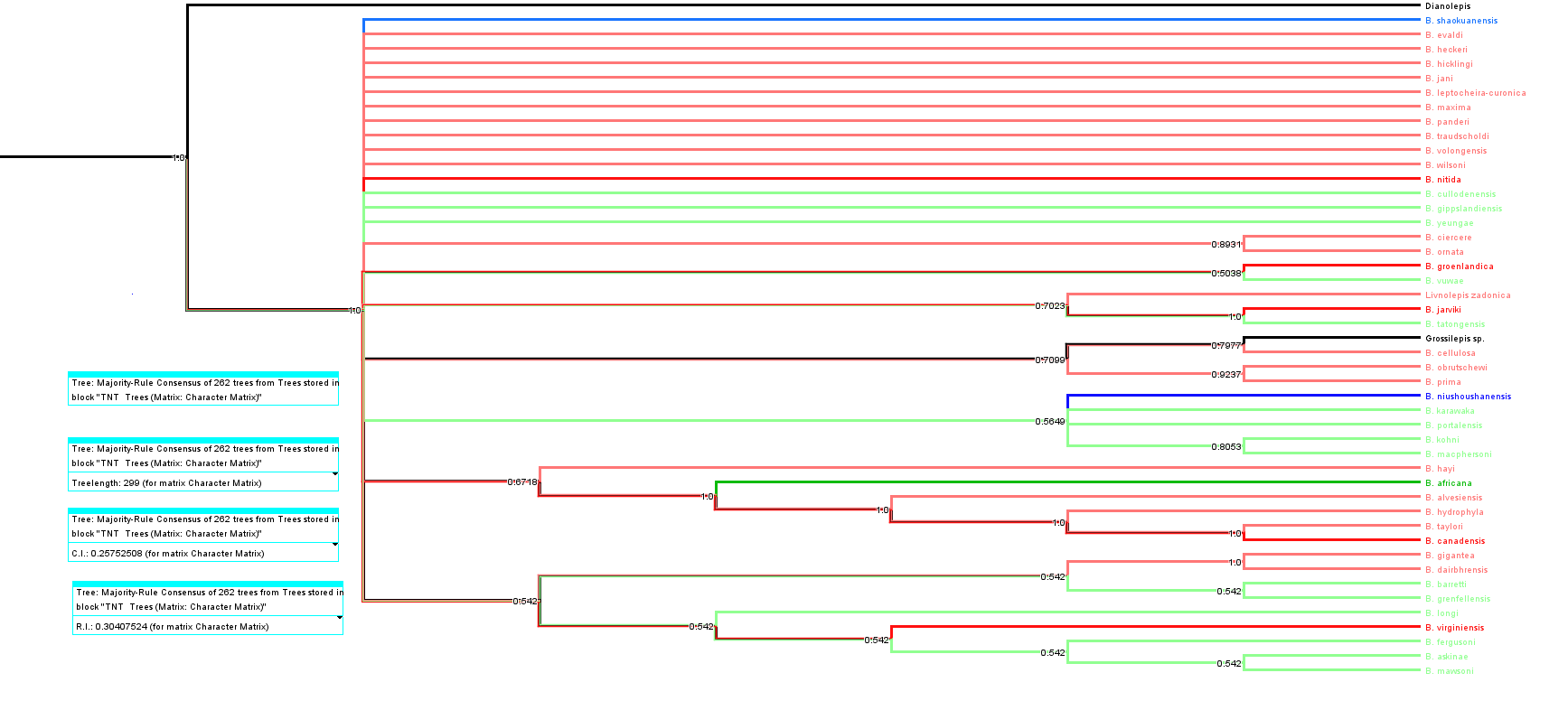


S31 Fig. Majority rule consensus tree of the phylogeny of the genus *Bothriolepis*, including *Grossilepis* and *Dianolepis*, using the reduced data set (49 characters * 47 taxa). Colours indicate palaeogeographic affinities of taxa (red Laurussia, green Gondwana, blue China; black is outgroup).

### Outgroup = Dianolepis

n=386; L=226; CI=0.332; RI=0.505; L_50_=295; CI_50_=0.254; RI_50_=0.279.

The majority rule consensus tree shows a basal polytomy, and no basal branch can be identified; *Grossilepis* is resolved as sister group for *B. cellulosa*, and the clade branches from the basal polytomy; the Chinese species are resolved as a clade branching from the basal polytomy.


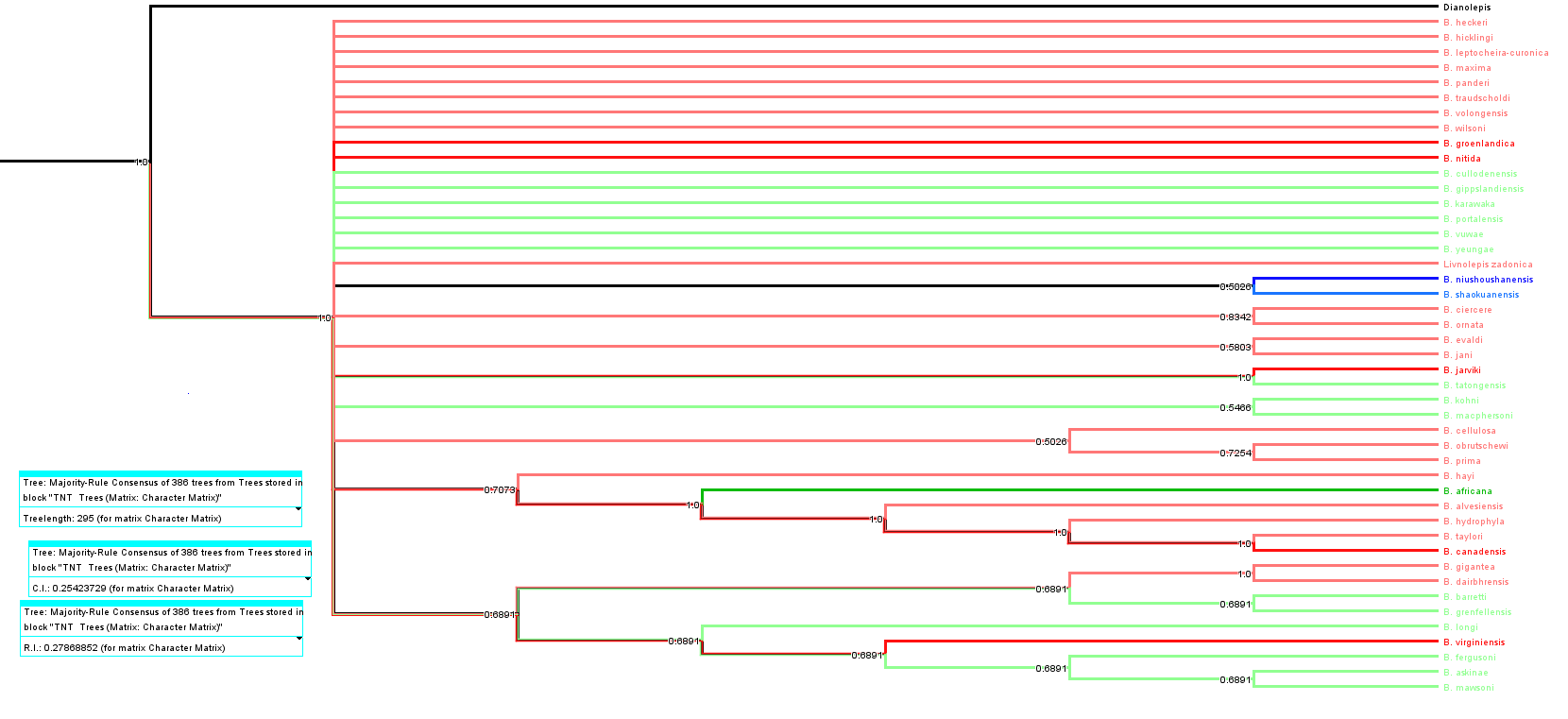
 S32 Fig. Majority rule consensus tree of the phylogeny of the genus *Bothriolepis*, including *Dianolepis*, using the reduced data set (49 characters * 46 taxa). Colours indicate palaeogeographic affinities of taxa (red Laurussia, green Gondwana, blue China; black is outgroup).

## Successive weighting of character “shape of preorbital recess”

### indices

|  | | |  |  |  |  |  |  |  |  |  |
| --- | --- | --- | --- | --- | --- | --- | --- | --- | --- | --- | --- |
| **weight prh** | **n** | **L** | **CI** | **RI** | **CI_prh_** | **RI_prh_** | **L_50%_** | **CI_50%_** | **RI_50%_** | **CI_50%prh_** | **RI_50%prh_** |
| 1 | 96 | 210 | 333 | 509 | 364 | 562,5 | 251 | 279 | 365 | 333 | 500 |
| 2 | 268 | 219 | 338 | 518 | 667 | 875 | 247 | 300 | 425 | 400 | 625 |
| 3 | 154 | 225 | 470 | 536 | 800 | 935 | 232 | 336 | 514 | 667 | 875 |
| 4 | 30 | 230 | 357 | 556 | 800 | 937,5 | 233 | 352 | 547 | 800 | 937,5 |
| 5 | 67 | 235 | 366 | 573 | 1000 | 1000 | 249 | 345 | 533 | 800 | 938 |
| 6 | 34 | 239 | 377 | 592 | 1000 | 1000 | 243 | 370 | 581 | 1000 | 1000 |
| 7 | 34 | 243 | 387 | 609 | 1000 | 1000 | 247 | 381 | 598 | 1000 | 1000 |
| 8 | 34 | 247 | 397 | 625 | 1000 | 1000 | 251 | 390 | 615 | 1000 | 1000 |

Table D: “Evolution” of indices related to the reweight of the character “shape of the preorbital recess” (all CI and RI values are multiplied by 100).

S33 Fig. “Evolution” of indices related to the reweight of the character “shape of the preorbital recess” (all CI and RI values are multiplied by 100). n, number of equiparsimonious trees; L, length of each equiparsimonious tree; CI, consistency index of each equiparsimonious tree; RI, retention index of each equiparsimonious tree; CIprh, consistency index of characters #1 for each equiparsimonious tree; RIprh, retention index of characters #1 for each equiparsimonious tree; L50, length of majority rule consensus tree; CI50, consistency index of majority rule consensus tree; RI50, retention index of majority rule consensus tree; CI50prh, consistency index of characters #1 for majority rule consensus tree; RI50prh, retention index of characters #1 for majority rule consensus tree

### Topologies and distribution of states of character #1

#### w=2

Palaeogeographic aspect


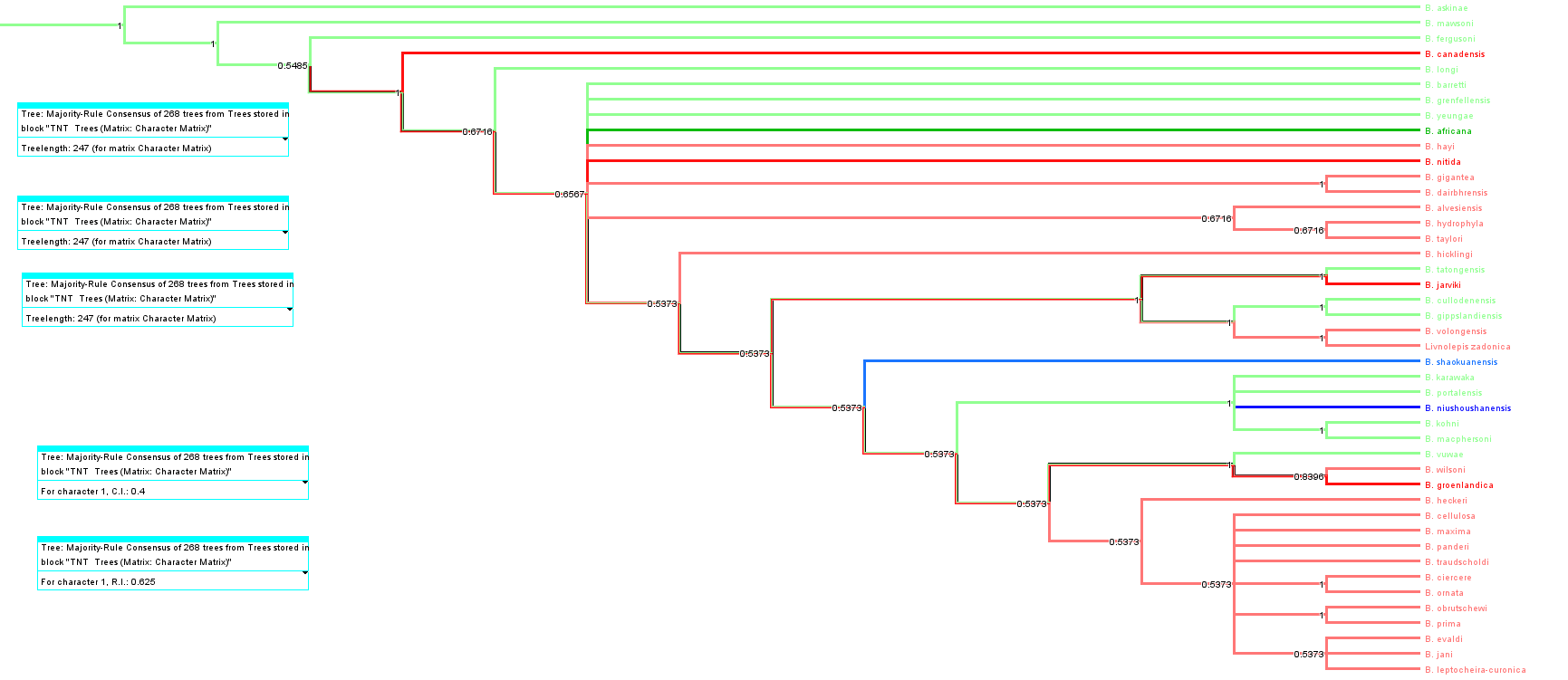


S34 Fig. Majority rule consensus tree of the phylogeny of the genus *Bothriolepis* using the reduced data set (49 characters * 46 taxa); reweight of character #1 = 2. Colours indicate palaeogeographic affinities of taxa (red Laurussia, green Gondwana, blue China; black is outgroup).

History of character 1


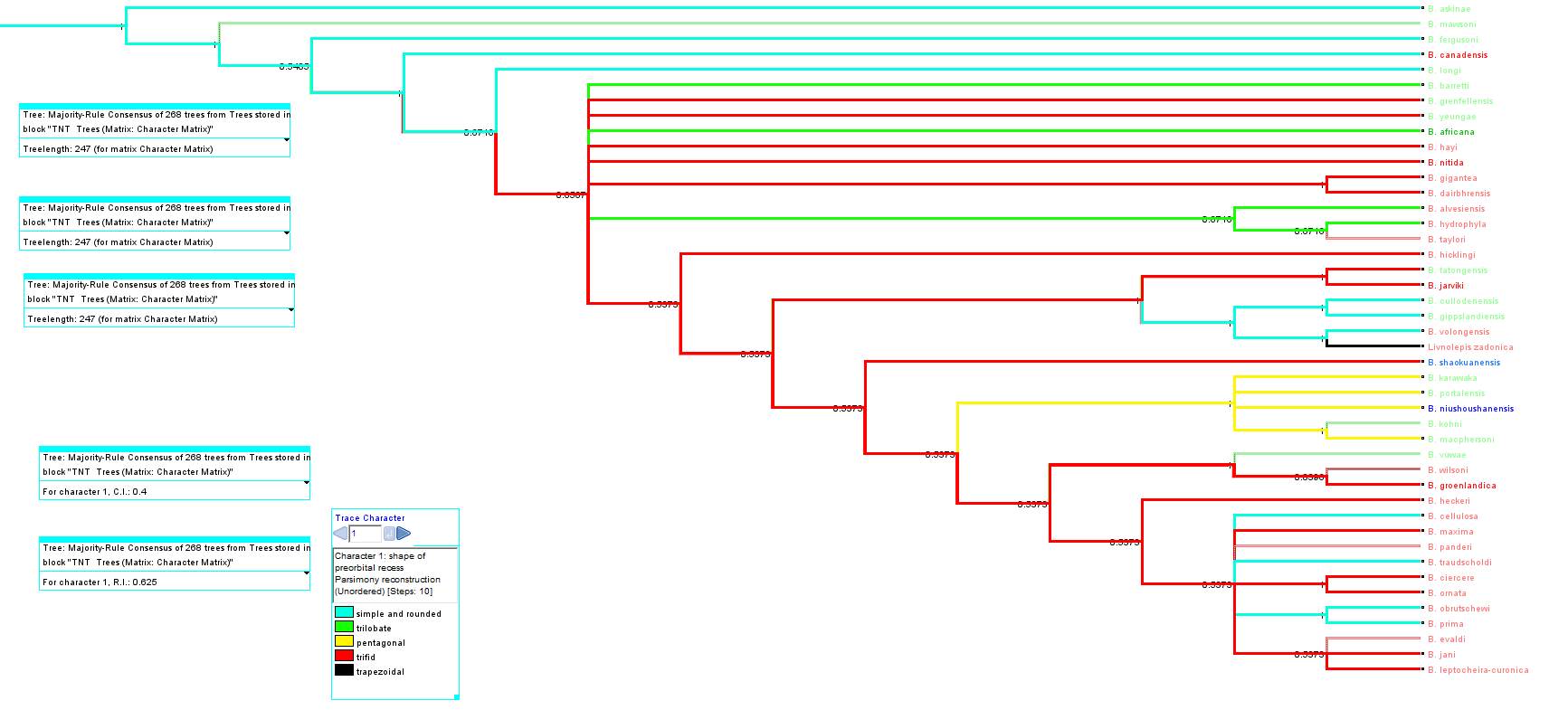


S35 Fig. Majority rule consensus tree of the phylogeny of the genus *Bothriolepis* using the reduced data set (49 characters * 46 taxa); reweight of character #1 = 2. Colours indicate distribution of preorbital recess shape (character #1; blue simple and rounded, green trilobate, yellow pentagonal, red trifid, black trapezoidal).

#### W=3

Palaeogeographic aspect


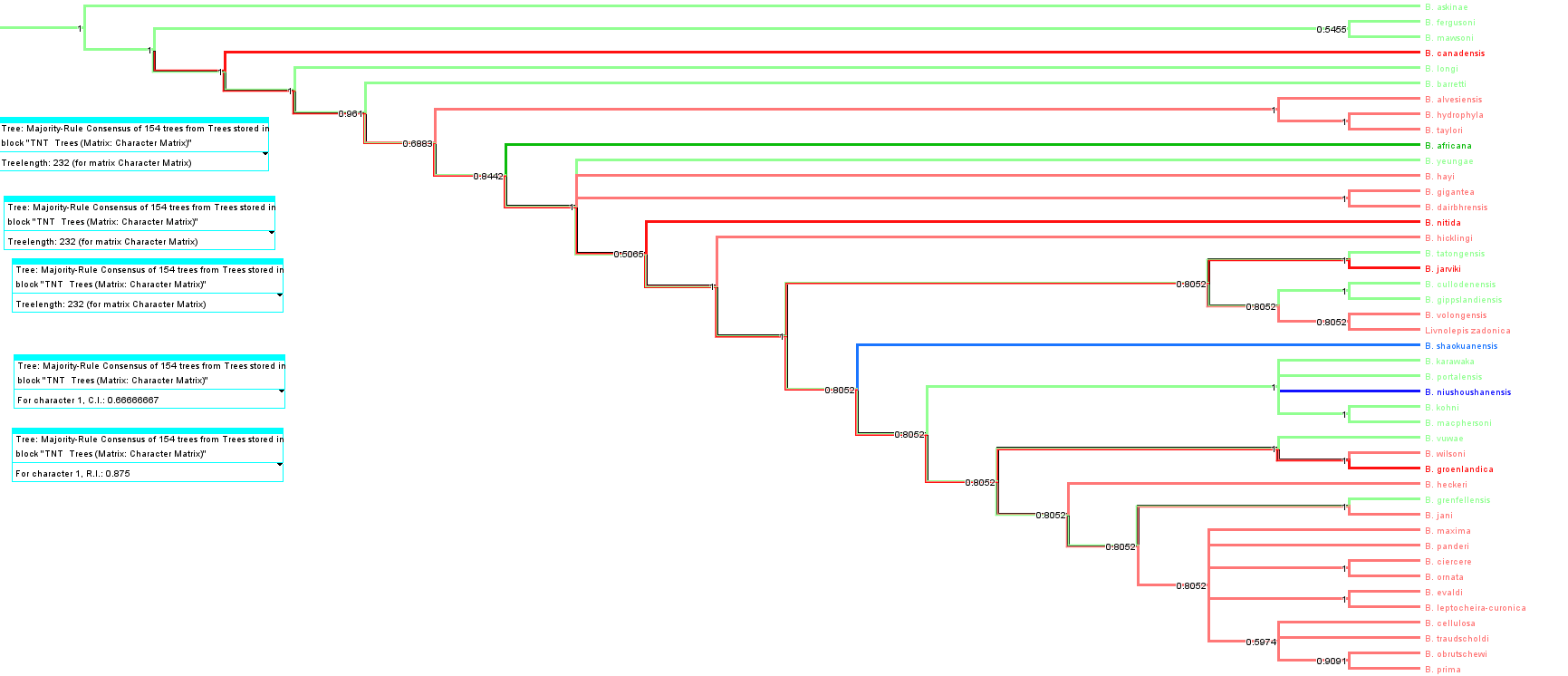


S36 Fig. Majority rule consensus tree of the phylogeny of the genus *Bothriolepis* using the reduced data set (49 characters * 46 taxa); reweight of character #1 = 3. Colours indicate palaeogeographic affinities of taxa (red Laurussia, green Gondwana, blue China; black is outgroup).

History of character 1


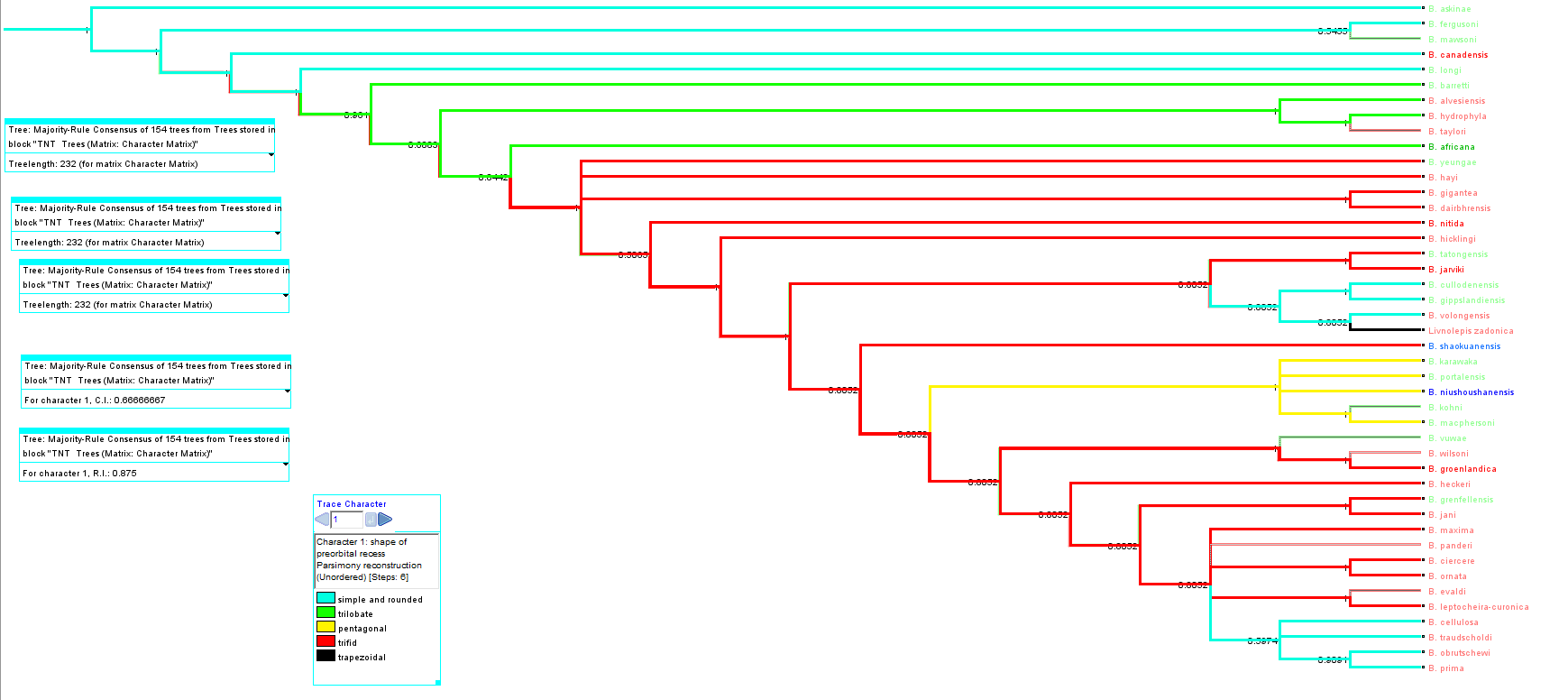


S37 Fig. Majority rule consensus tree of the phylogeny of the genus *Bothriolepis* using the reduced data set (49 characters * 46 taxa); reweight of character #1 = 3. Colours indicate distribution of preorbital recess shape (character #1; blue simple and rounded, green trilobate, yellow pentagonal, red trifid, black trapezoidal).

#### W=4

Palaeogeographic aspect


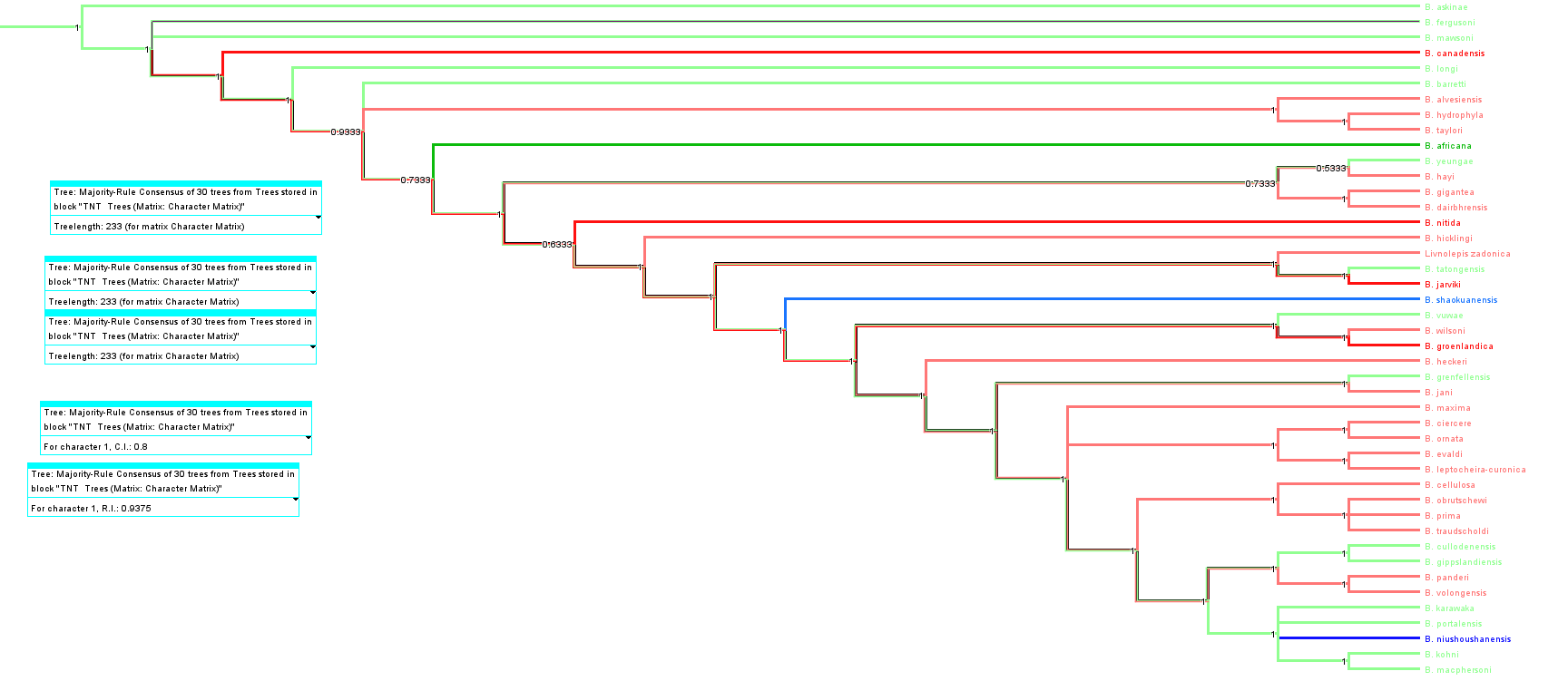


S38 Fig. Majority rule consensus tree of the phylogeny of the genus *Bothriolepis* using the reduced data set (49 characters * 46 taxa); reweight of character #1 = 4. Colours indicate palaeogeographic affinities of taxa (red Laurussia, green Gondwana, blue China; black is outgroup).

History of character 1


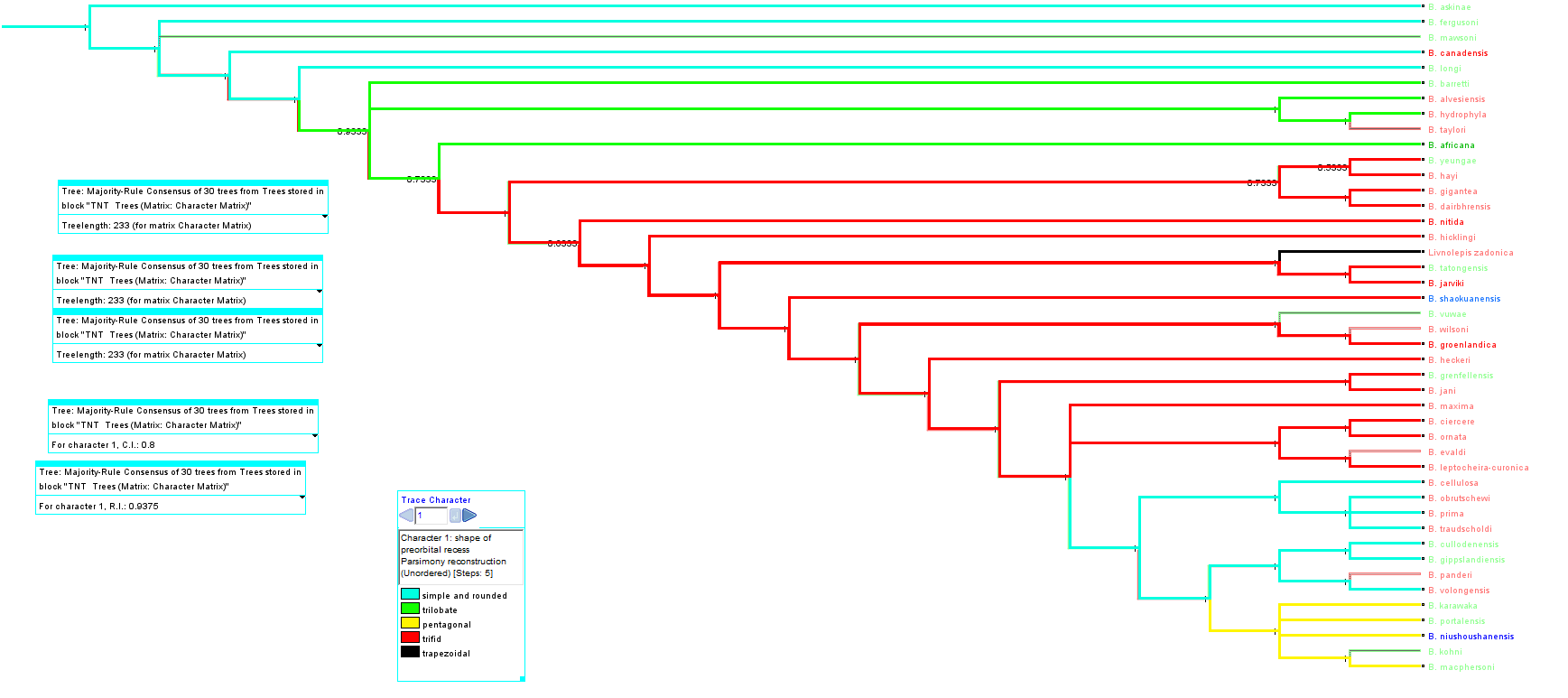


S39. Fig. Majority rule consensus tree of the phylogeny of the genus *Bothriolepis* using the reduced data set (49 characters * 46 taxa); reweight of character #1 = 4. Colours indicate distribution of preorbital recess shape (character #1; blue simple and rounded, green trilobate, yellow pentagonal, red trifid, black trapezoidal).

#### W=5

Palaeogeographic aspect


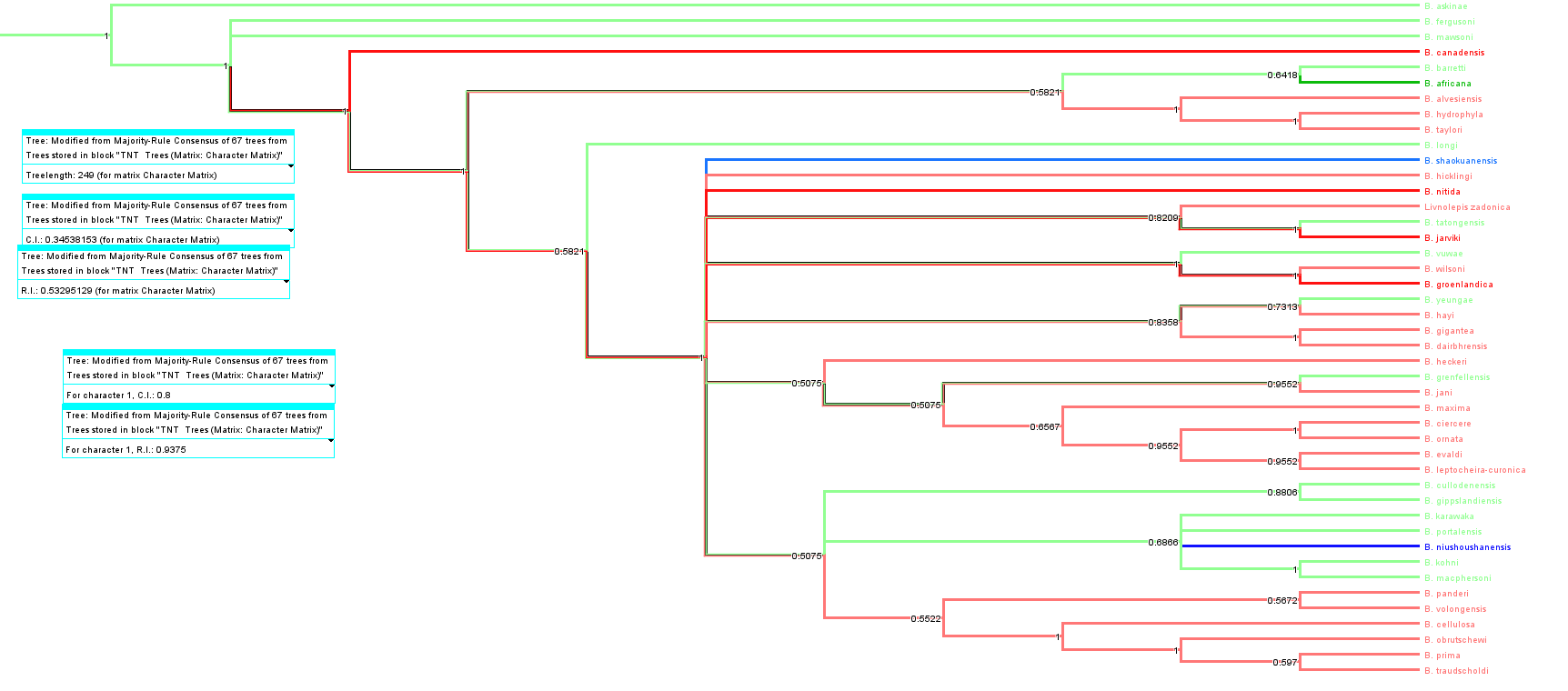


S40 Fig. Majority rule consensus tree of the phylogeny of the genus *Bothriolepis* using the reduced data set (49 characters * 46 taxa); reweight of character #1 = 5. Colours indicate palaeogeographic affinities of taxa (red Laurussia, green Gondwana, blue China; black is outgroup).

History of character 1


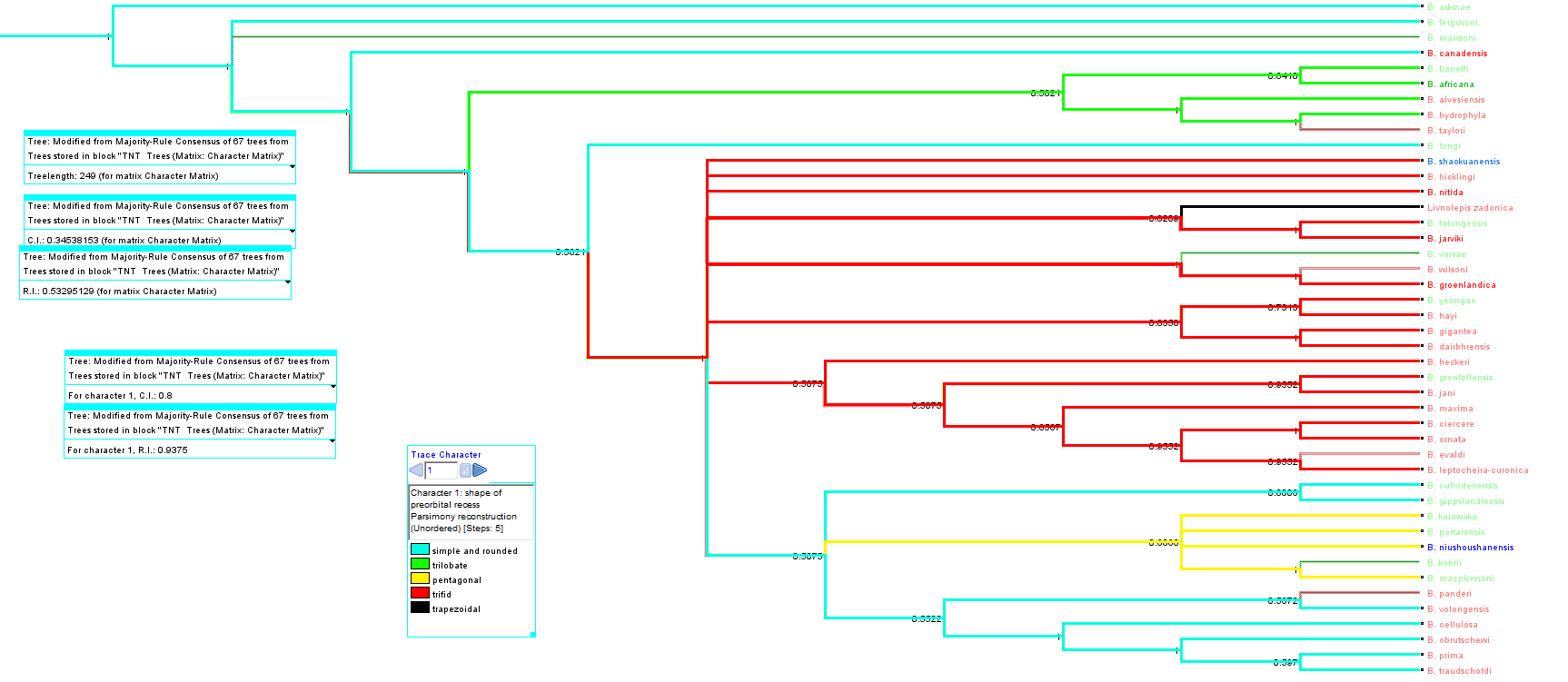


S41 Fig. Majority rule consensus tree of the phylogeny of the genus *Bothriolepis* using the reduced data set (49 characters * 46 taxa); reweight of character #1 = 5. Colours indicate distribution of preorbital recess shape (character #1; blue simple and rounded, green trilobate, yellow pentagonal, red trifid, black trapezoidal).

#### W=6

Palaeogeographic aspect


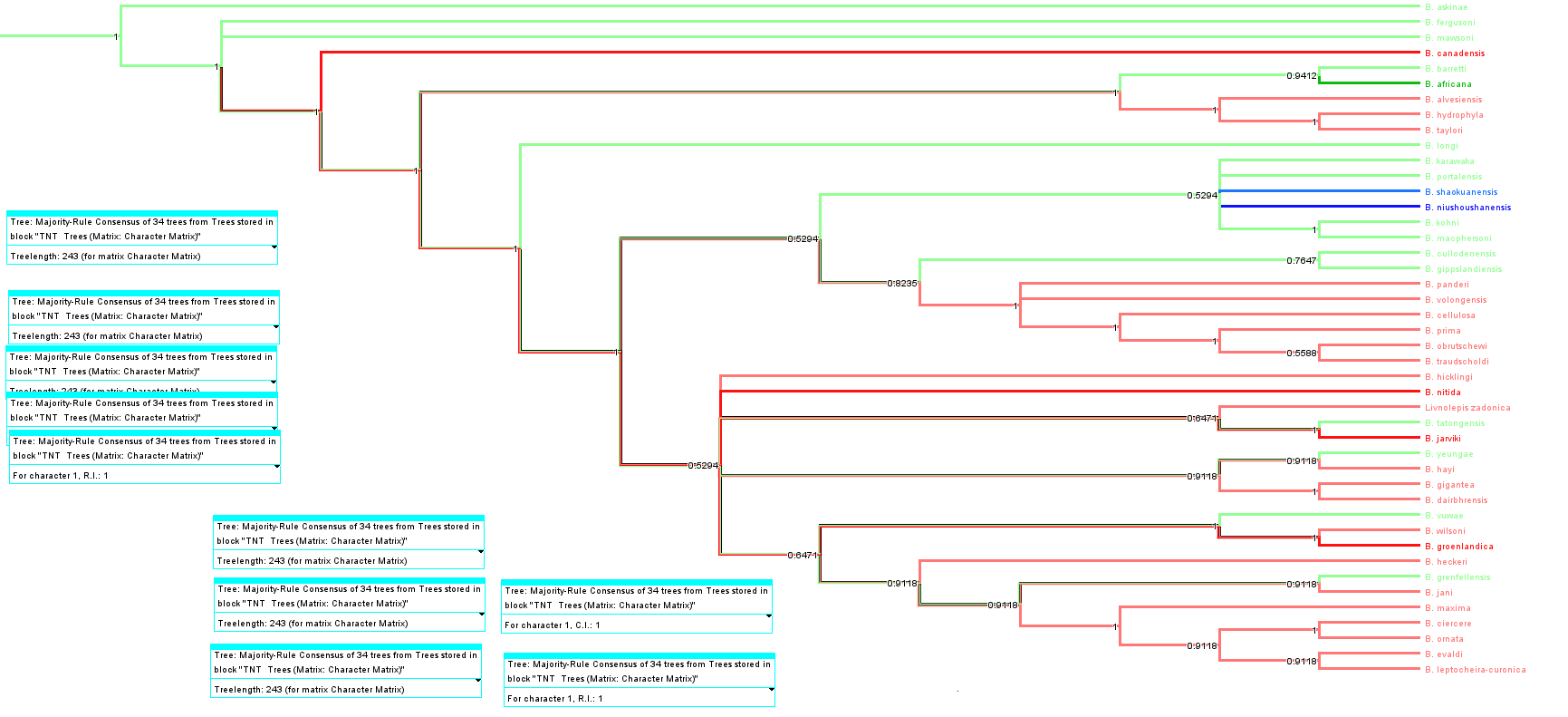


S42 Fig. Majority rule consensus tree of the phylogeny of the genus *Bothriolepis* using the reduced data set (49 characters * 46 taxa); reweight of character #1 = 6. Colours indicate palaeogeographic affinities of taxa (red Laurussia, green Gondwana, blue China; black is outgroup).

History of character 1


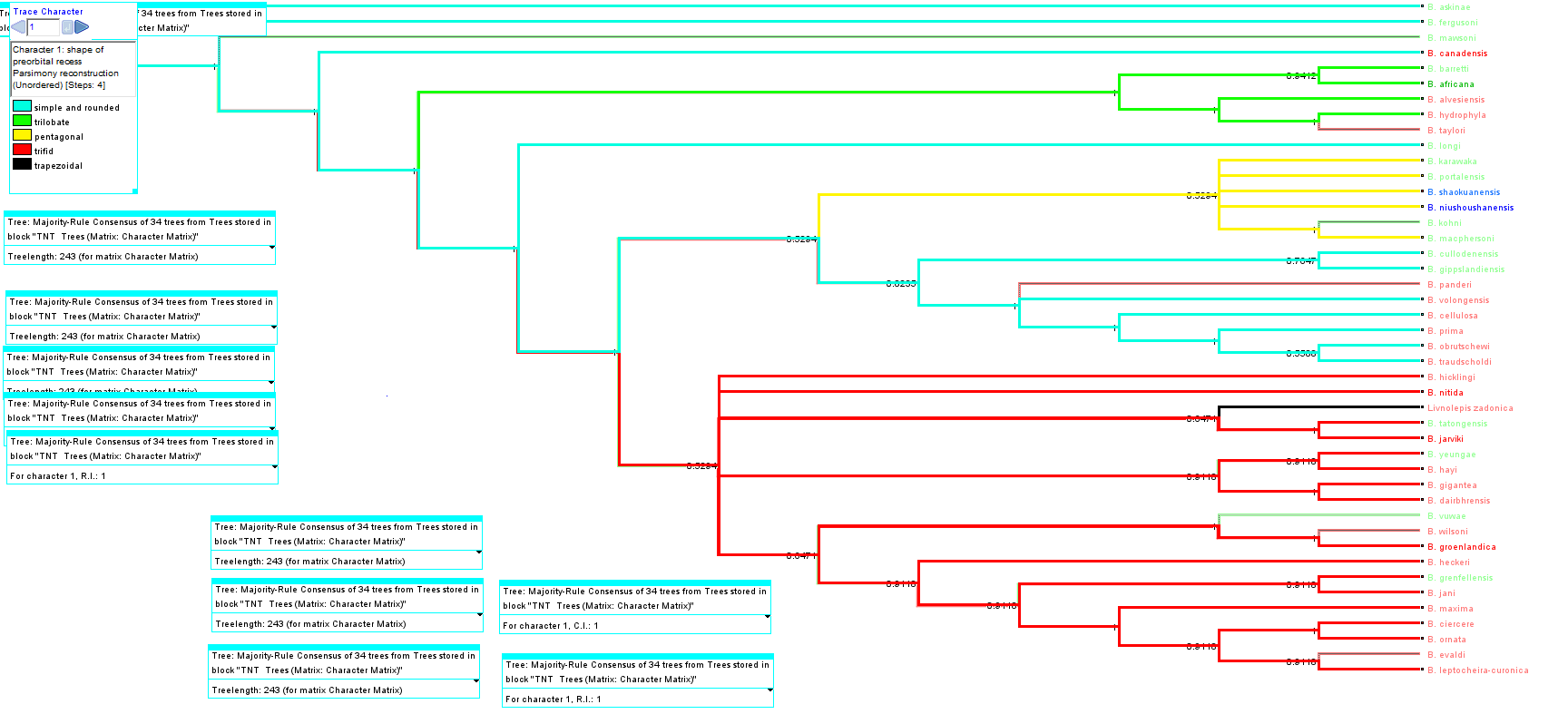


S43 Fig. Majority rule consensus tree of the phylogeny of the genus *Bothriolepis* using the reduced data set (49 characters * 46 taxa); reweight of character #1 = 6. Colours indicate distribution of preorbital recess shape (character #1; blue simple and rounded, green trilobate, yellow pentagonal, red trifid, black trapezoidal).

#### W=7

Palaeogeographic aspect


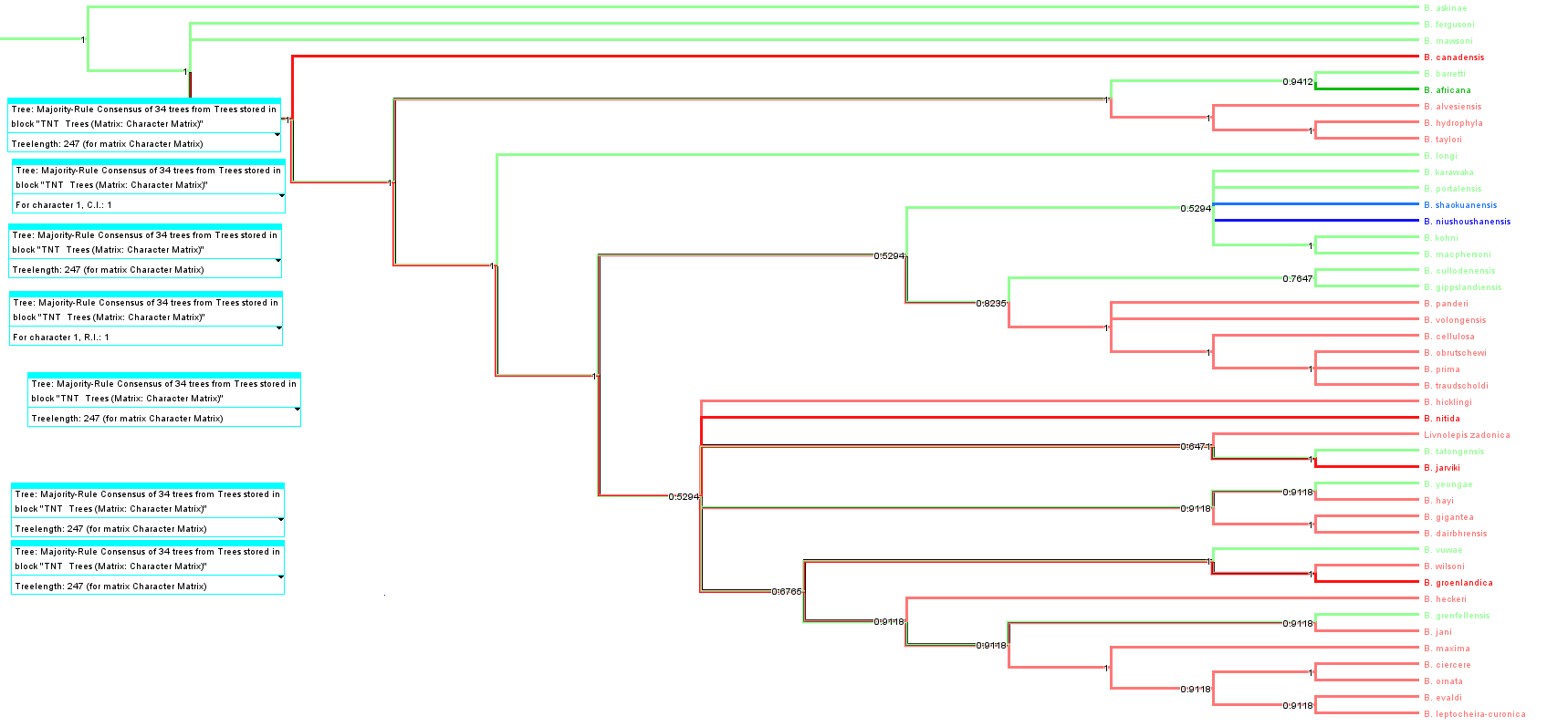


S44 Fig. Majority rule consensus tree of the phylogeny of the genus *Bothriolepis* using the reduced data set (49 characters * 46 taxa); reweight of character #1 = 7. Colours indicate palaeogeographic affinities of taxa (red Laurussia, green Gondwana, blue China; black is outgroup).

History of character 1


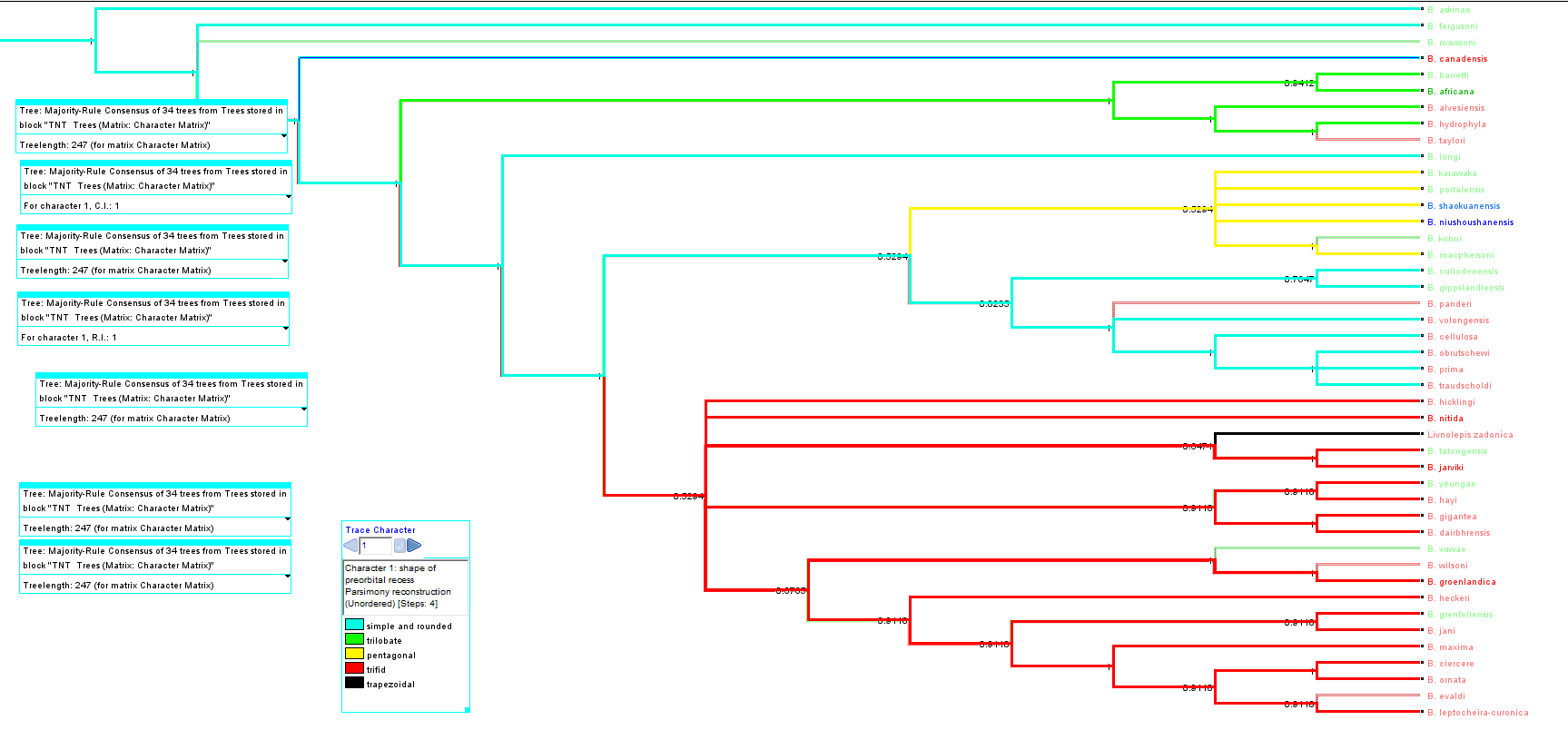


S45 Fig. Majority rule consensus tree of the phylogeny of the genus *Bothriolepis* using the reduced data set (49 characters * 46 taxa); reweight of character #1 = 7. Colours indicate distribution of preorbital recess shape (character #1; blue simple and rounded, green trilobate, yellow pentagonal, red trifid, black trapezoidal).

#### W=8

Palaeogeographic aspect


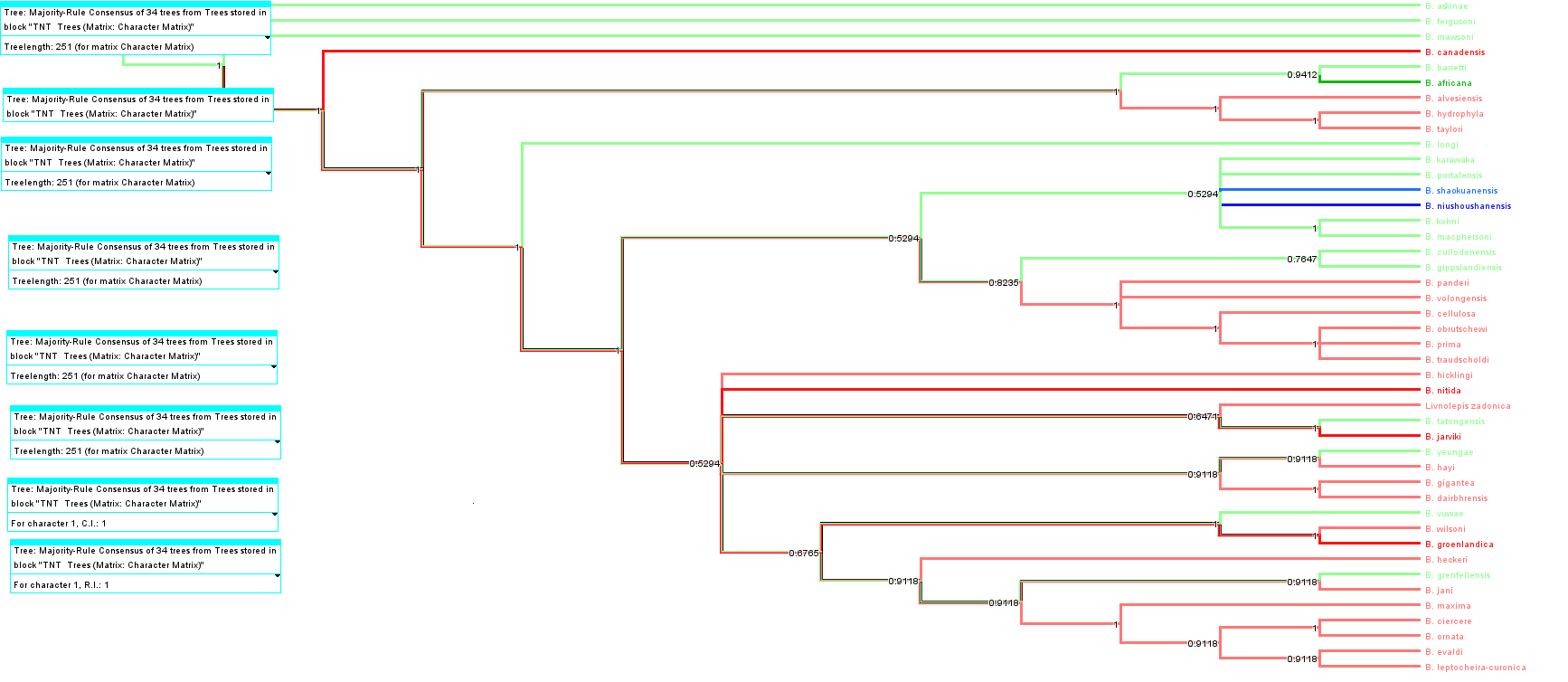


S46 Fig. Majority rule consensus tree of the phylogeny of the genus *Bothriolepis* using the reduced data set (49 characters * 46 taxa); reweight of character #1 =8. Colours indicate palaeogeographic affinities of taxa (red Laurussia, green Gondwana, blue China; black is outgroup).

History of character 1

–––––
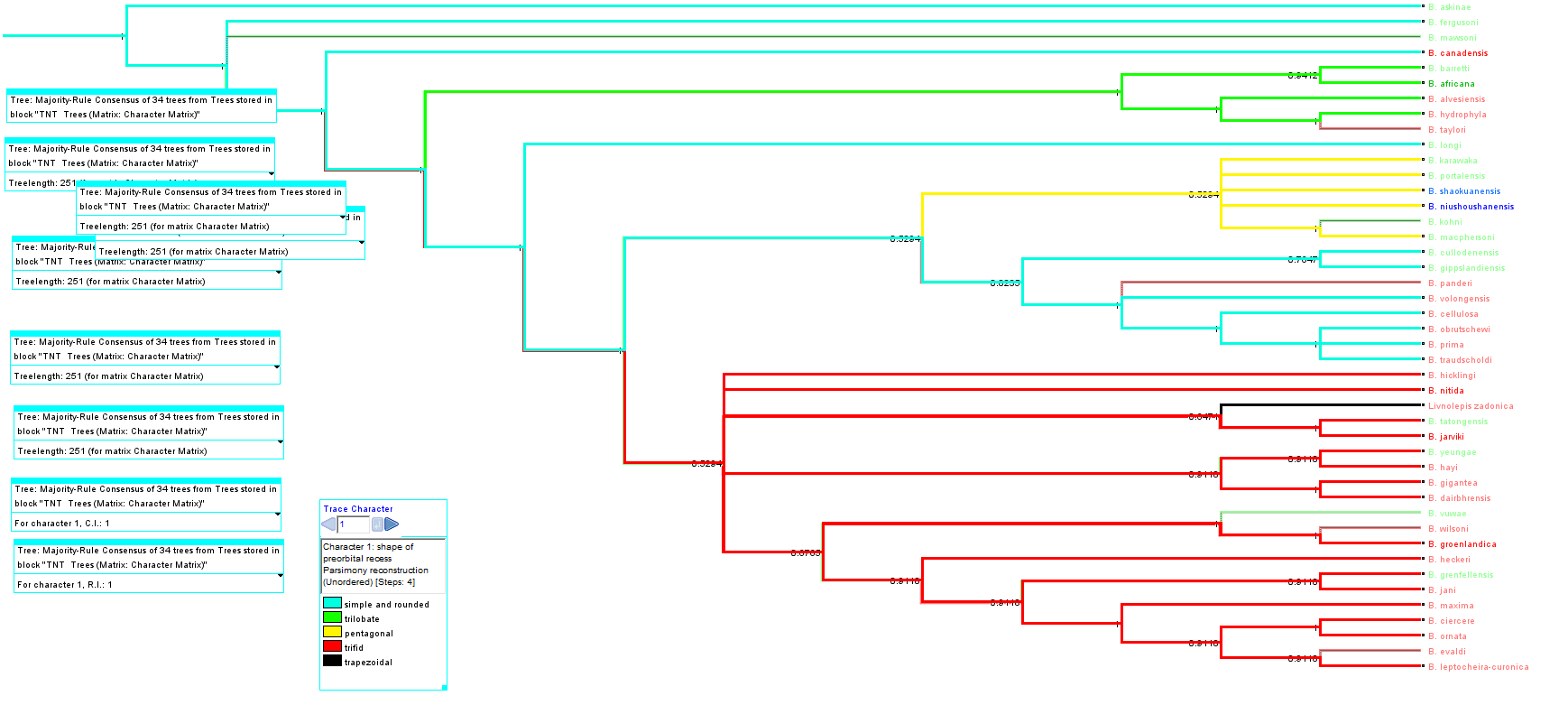


S47 Fig. Majority rule consensus tree of the phylogeny of the genus *Bothriolepis* using the reduced data set (49 characters * 46 taxa); reweight of character #1 = 8. Colours indicate distribution of preorbital recess shape (character #1; blue simple and rounded, green trilobate, yellow pentagonal, red trifid, black trapezoidal).

## Robustness of the tree: Bremer decay index


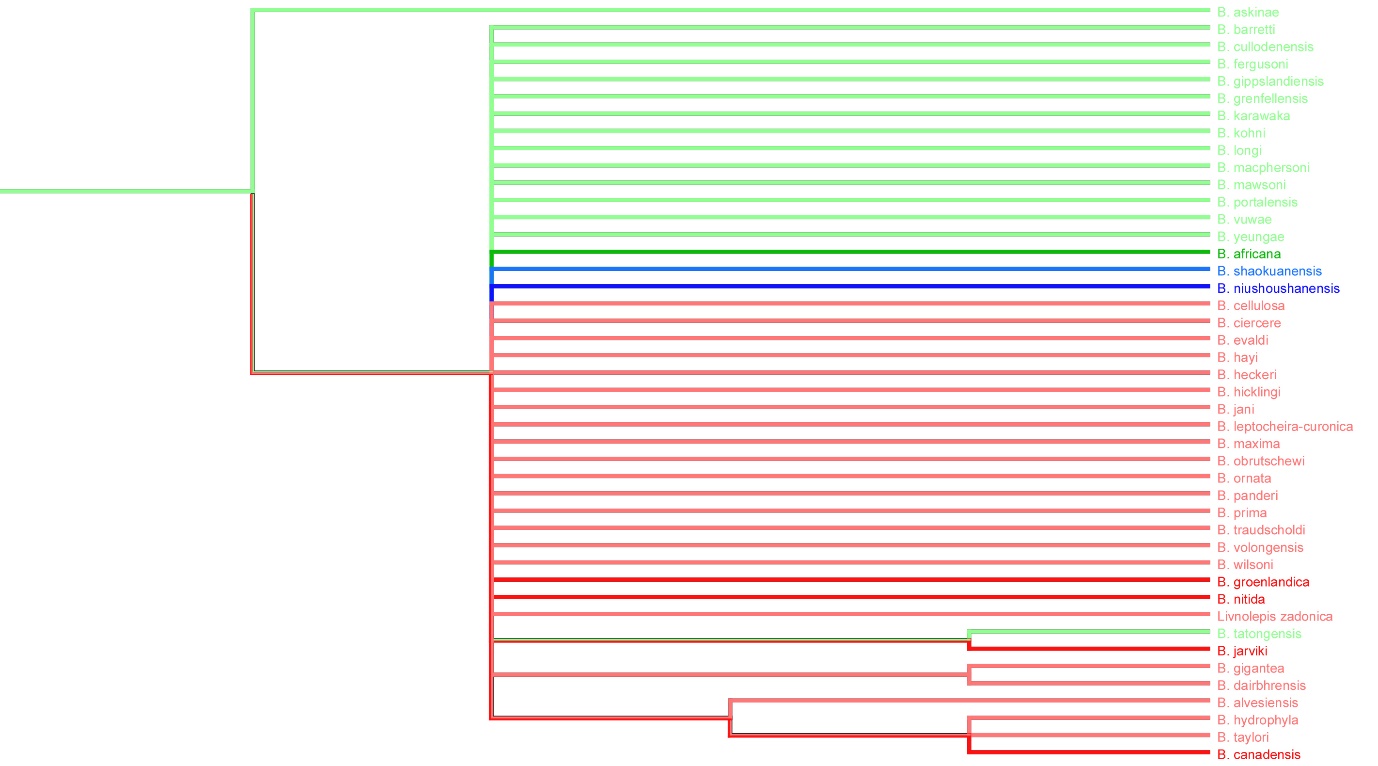


S48 Fig. Strict consensus tree of the phylogeny of the genus *Bothriolepis* using the reduced data set (49 characters * 46 taxa) with Bremer index BI = 1 (n = 12 629 trees). Colours indicate palaeogeographic affinities of taxa (red Laurussia, green Gondwana, blue China; black is outgroup).


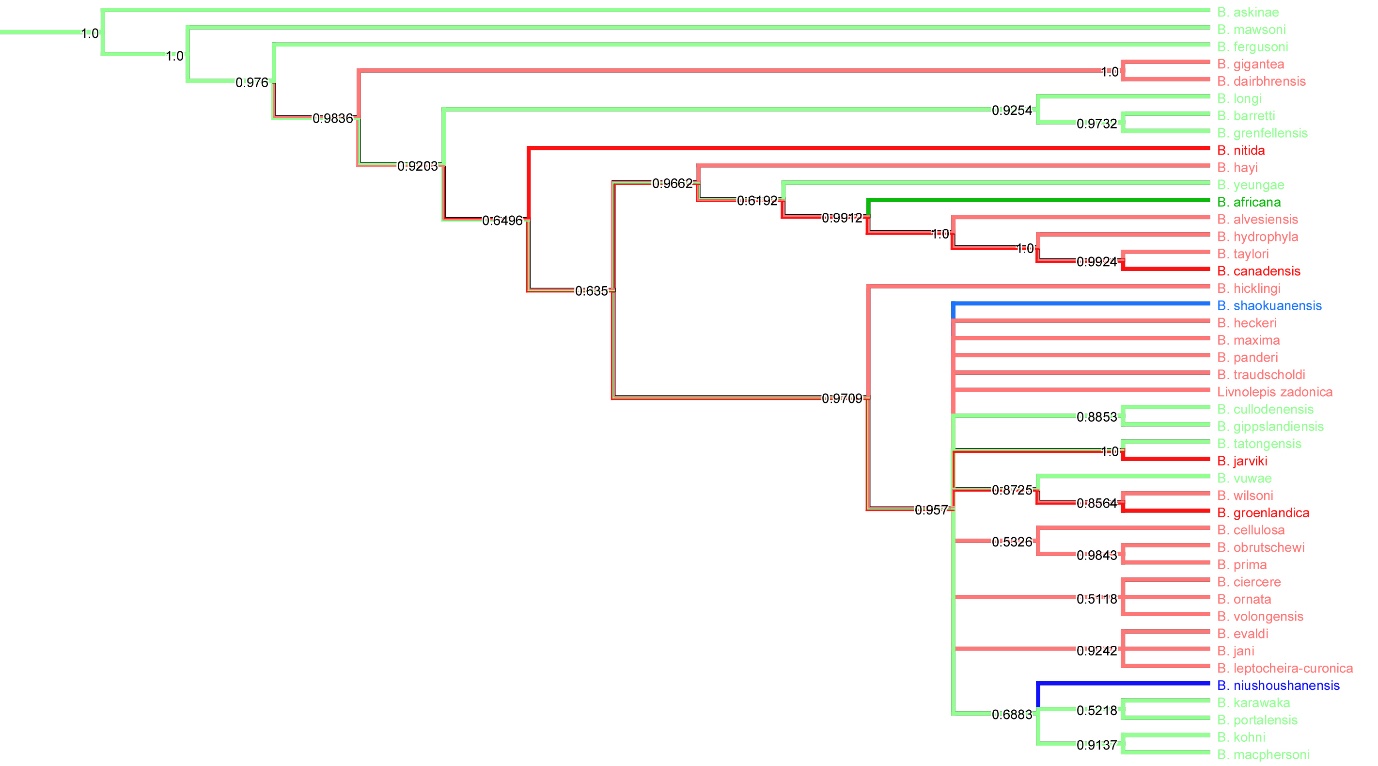


S49 Fig. Majority Rule consensus tree of the phylogeny of the genus *Bothriolepis* using the reduced data set (49 characters * 46 taxa) with Bremer index BI = 1 (n = 12 629 trees). Values on branches indicate retained frequency. Colours indicate palaeogeographic affinities of taxa (red Laurussia, green Gondwana, blue China; black is outgroup).


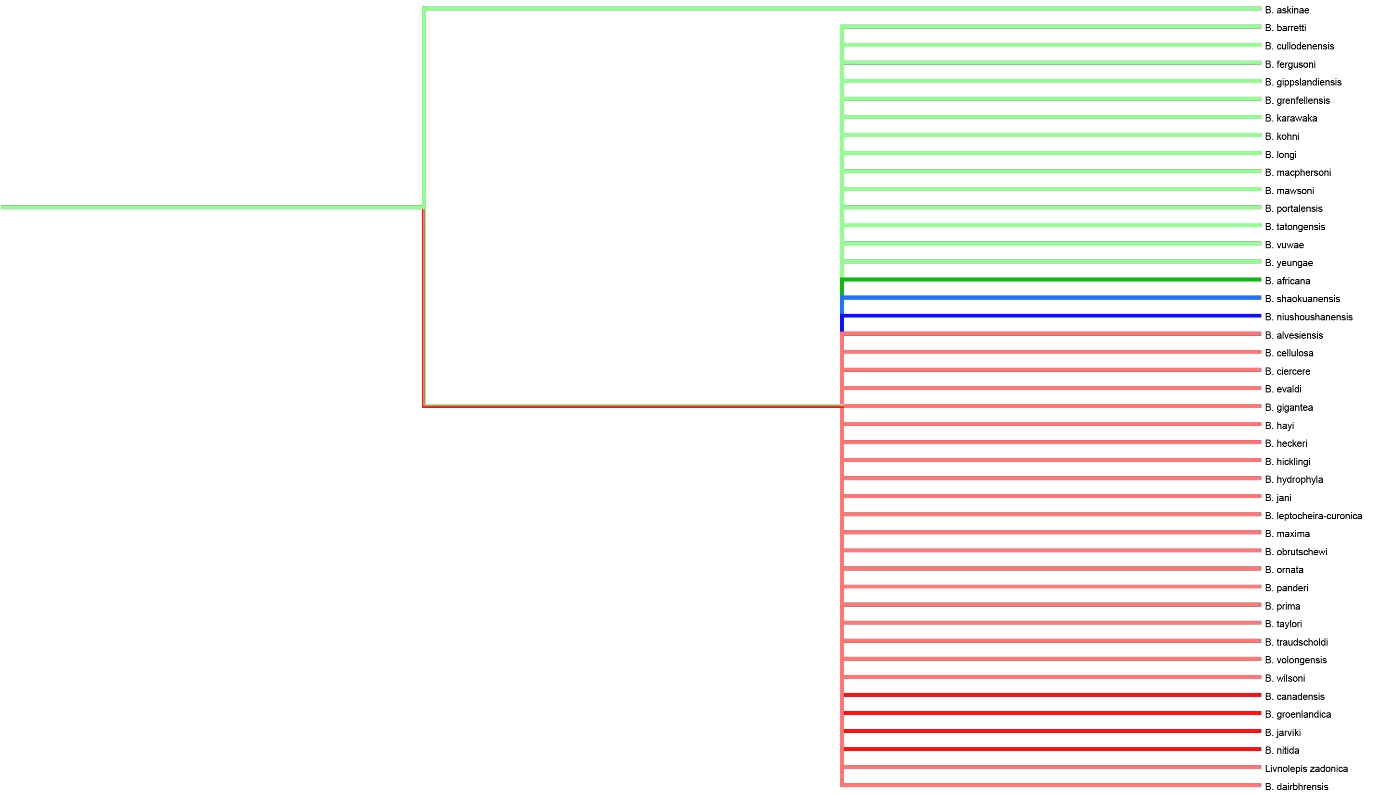


S50 Fig. Strict consensus tree of the phylogeny of the genus *Bothriolepis* using the reduced data set (49 characters * 46 taxa) with Bremer index BI ≥ 2 (n = 1 000 000 trees). Colours indicate palaeogeographic affinities of taxa (red Laurussia, green Gondwana, blue China; black is outgroup).


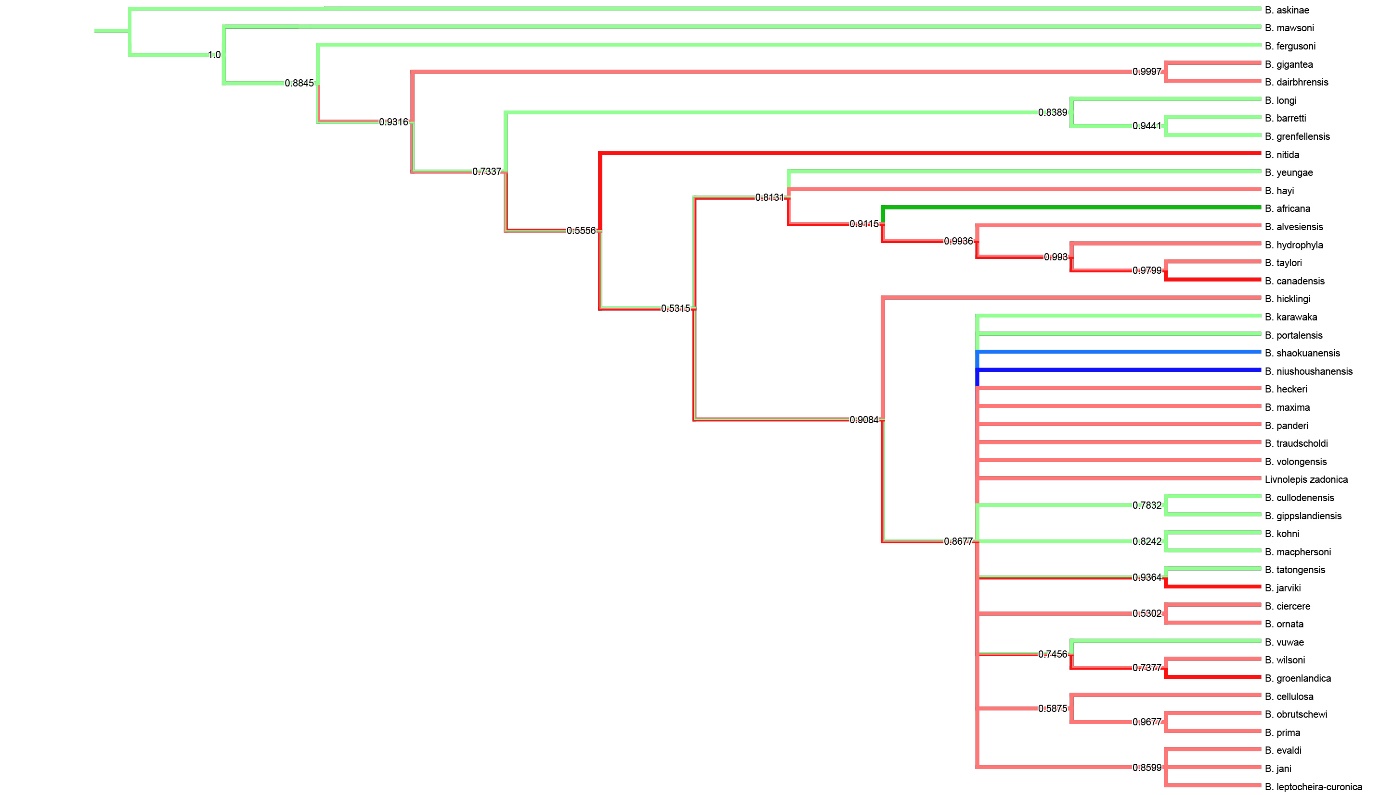


S51 Fig. Majority Rule consensus tree of the phylogeny of the genus *Bothriolepis* using the reduced data set (49 characters * 46 taxa) with Bremer index BI = 2 (n = 1 000 000 trees). Values on branches indicate retained frequency. Colours indicate palaeogeographic affinities of taxa (red Laurussia, green Gondwana, blue China; black is outgroup).


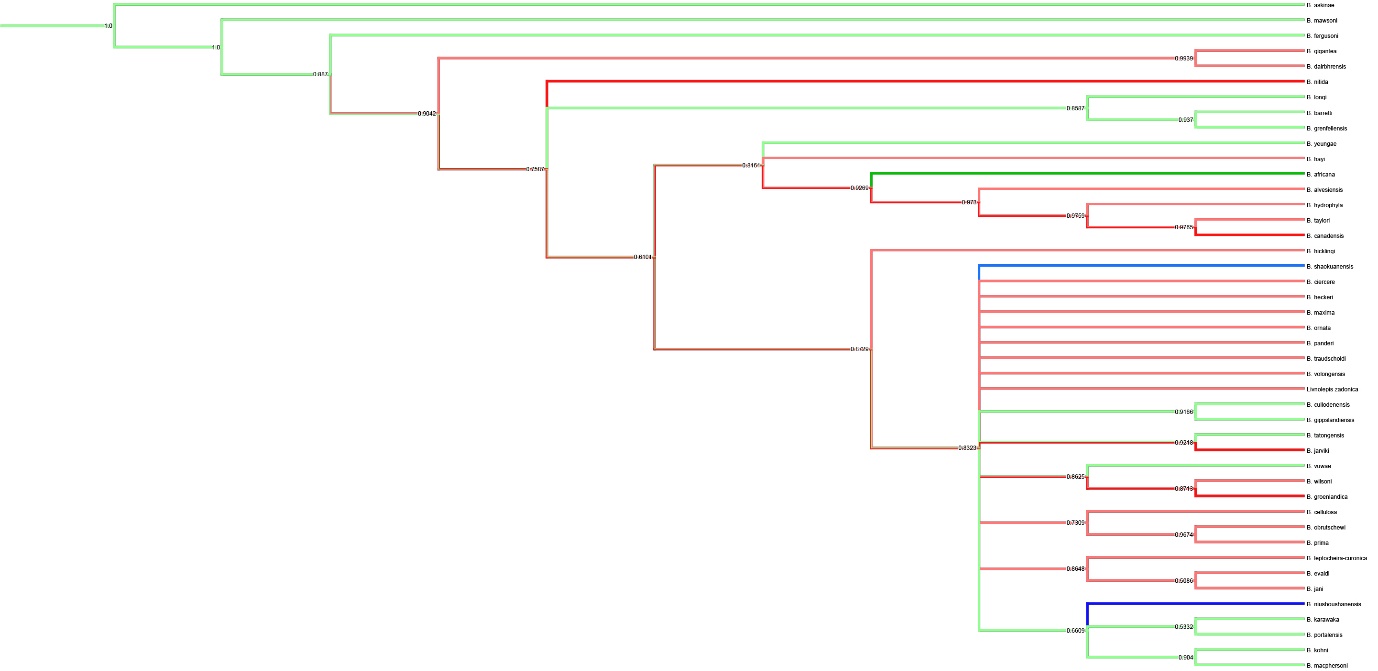


S52 Fig. Majority Rule consensus tree of the phylogeny of the genus *Bothriolepis* using the reduced data set (49 characters * 46 taxa) with Bremer index BI = 3 (n = 1 000 000 trees). Values on branches indicate retained frequency. Colours indicate palaeogeographic affinities of taxa (red Laurussia, green Gondwana, blue China; black is outgroup).


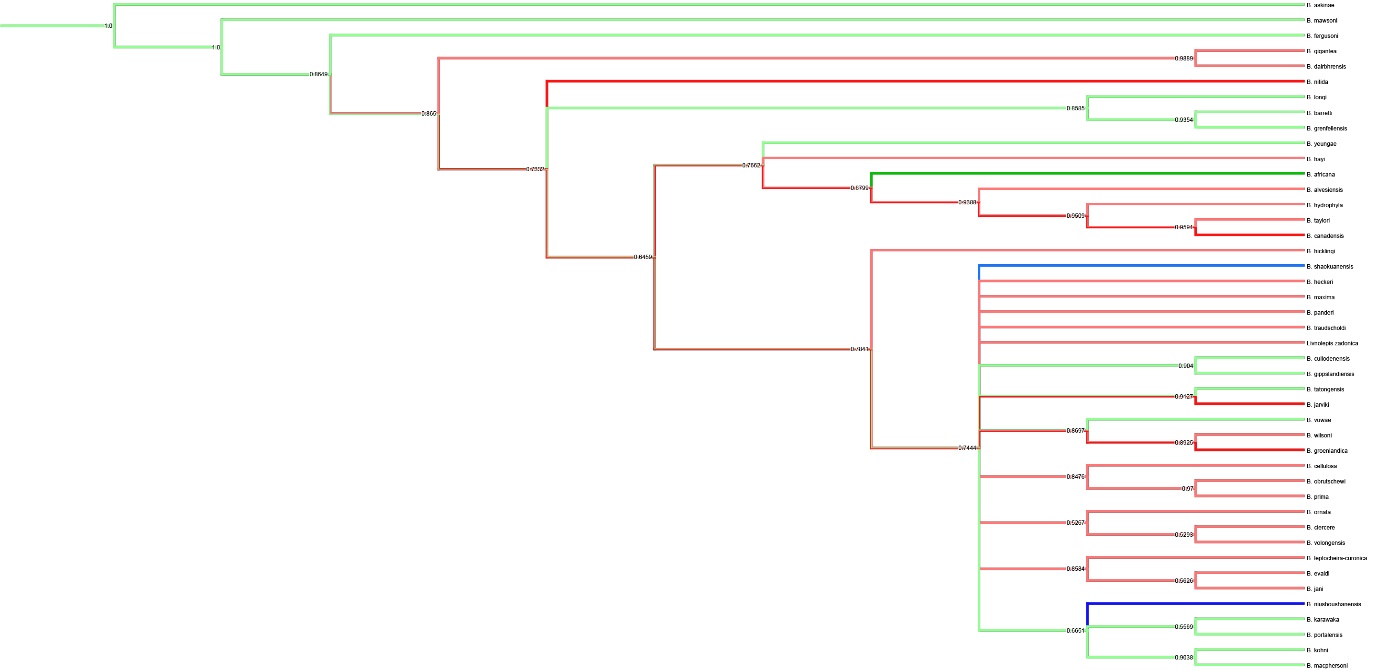


S53 Fig. Majority Rule consensus tree of the phylogeny of the genus *Bothriolepis* using the reduced data set (49 characters * 46 taxa) with Bremer index BI = 4 (n = 1 000 000 trees). Values on branches indicate retained frequency. Colours indicate palaeogeographic affinities of taxa (red Laurussia, green Gondwana, blue China; black is outgroup).


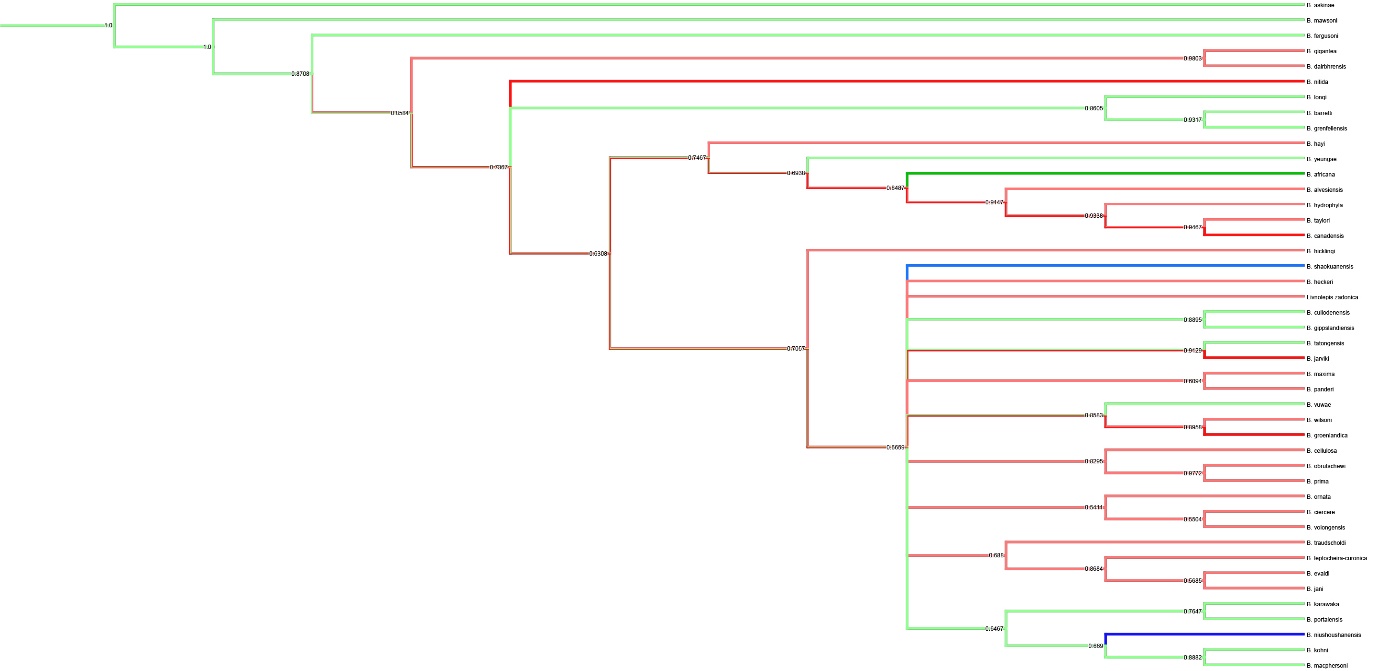
S54. Fig. Majority Rule consensus tree of the phylogeny of the genus *Bothriolepis* using the reduced data set (49 characters * 46 taxa) with Bremer index BI = 5 (n = 1 000 000 trees). Values on branches indicate retained frequency. Colours indicate palaeogeographic affinities of taxa (red Laurussia, green Gondwana, blue China; black is outgroup).

# 3D pdf captions

In each 3D-pdf, it is possible to change colour of the background, as well as light intensity. Each structure can be rendered opaque, semi-transparent or invisible. Depending on your OS and viewer, you may be prompted to manually authorize opening of the file. Each element can be selected manually either in the tree or in the 3D window for identification.

## NMING:F35203-UU201ab_HQ

*Bothriolepis dairbhrensis* sp. nov. Incomplete lateral plate (better rendering on Drishti) and fragment of indeterminate plate. Segmentation in Mimics, treatment of STLS and 3d PDF exportation in 3-matic. Scale cube edge is 10 mm

## NMING:F35229-CH003

*Bothriolepis dairbhrensis* sp. nov. Assemblage of indeterminate plate and indeterminate scavenging gastropods. Segmentation in Mimics, treatment of STLS and 3d PDF exportation in 3-matic. Scale cube edge is 10 mm.

## NMING:F35216-UUSFB001

*Bothriolepis dairbhrensis* sp. nov. Proximal segment of the pectoral fin, with AVL and articular condyle. [ORG] corresponds to plates in original position; [RELOC] corresponds to plates relocated in life position. Segmentation in Mimics, treatment of STLS and 3d PDF exportation in 3-matic. Scale cube edge is 10 mm.

## NHM P 59677 (complete, ORG end RELOC files)

*Bothriolepis dairbhrensis* sp. nov. Incomplete head (and endocranial processes), ventral body armour and proximal segment of the pectoral fin. [ORG] corresponds to plates in original position; [RELOC] corresponds to plates relocated in life position. Segmentation in Mimics, treatment of STLS and 3d PDF exportation in 3-matic. Scale cube edge is 10 mm.

## NHM P 59678

*Bothriolepis dairbhrensis* sp. nov. Incomplete lateral and indeterminate plates of an adult individual. Scale cube edge is 10 mm.

## NHM P 59679

*Bothriolepis dairbhrensis* sp. nov. Two indeterminate fragments. Scale cube edge is 10 mm.

## NHM P 59687

*Bothriolepis dairbhrensis* sp. nov. Indeterminate fragments and burrows. Scale cube edge is 10 mm.

# References

1. Eichwald E. Die Thier- und Pflanzenreste des alten rothen Sandsteins und Bergkalks im Novogrodschen Gouvernement. Bulletin Scientifique publié par l'Académie Impériale des Sciences de Saint-Pétersbourg et rédigé par son Secrétaire perpétuel. 1840;7(6-7):78–91.

2. Denison RH. Placodermi. Schultze H-P, editor. Stuttgart, New York: Gustav Fischer Verlag; 1978. 128 p.

3. Agassiz L. Monographie des poissons fossiles du Vieux Grès Rouge ou système Dévonien (Old Red Sandstone) des Isles britanniques et de Russie: Imprimerie Petitpierre, Neuchâtel et Soleure; 1844. 171 p.

4. Agassiz L. Lettres sur les poissons fossiles du système dévonien de la Russie. In: Murchinson RI, de Verneuil PE, von Keyserling A, editors. The geology of Russia and the Ural Mountains. London and Paris1845. p. 397–418.

5. Pander C, in Keyserling, A.,. Wissenschaftliche Beobachtungen auf einer Reise in das Petschora-Land. Saint-Petersburg1846. 106 p.

6. Keyserling A. Wissenschaftliche Beobachteungen auf eiпer Reise in das Petschora-Land. Saint Petersburg1846.

7. Leidy J. Descriptions of some remains of fishes from the Carboniferous and Devonian formations of the United States. Journal of the Academy of Natural Sciences, Philadelphia 2nd Series. 1856;3(3):156–60.

8. Egerton PdMG. On a new species of *Pterichthys* (*P. macrocephalus*, Egerton), from the yellow sandstone of Farlow, Co. Salop. Quaternary Journal of the Geological Society of London. 1862;18:103–8.

9. Lahusen J. Zur Kenntniss der Gattung *Bothriolepis* Eichw. . Verhandlungen der Russisch-Kaiserlichen Mineralogischen Gesellschaft zu Saint-Petersburg ser 2. 1880;15:125–38.

10. Whiteaves JF. On some remarkable fossil fishes from the Devonian rocks of Scaumenac Bay, in the Province of Quebec. Journal of Natural History Series 5. 1881;8(44):159–62.

11. Traquair RH. Notes on the nomenclature of the fishes of the Old Red Sandstone of Great Britain. Geological Magazine. 1888;5(3):507–17.

12. Newberry JS. The Paleozoic fishes of North America. Washington: Government Print Off; 1889. 340 p.

13. Thomson KS, Thomas B. On the status of species of *Bothriolepis* (Placodermi, Antiarchi) in North America. Journal of Vertebrate Paleontology. 2001;21(4):679–86.

14. Traquair RH. On the British species of Asterolepidae. Proceedings of the Royal Physical Society of Edinburgh. 1890-92;11:283–6.

15. Traquair RH. The extinct vertebrate animals of the Moray Firth area. In: Harvie-Brown JA, Buckley TE, editors. A vertebrate fauna of the Moray Firth basin. 2. Edinburgh1895. p. 235–85.

16. Rohon JV. Die devonischen Fische von Timan in Russland. Sitzungsberichte der Königlichen Böhmischen Gesellschaft der Wissenschaften, Mathematisch-naturwissenschaftliche Classe, 1899. 1900;8:1–77.

17. Lukševičs E, Beznosov P, Stūris V. A new assessment of the Late Devonian antiarchan fish *Bothriolepis leptocheira* from South Timan (Russia) and the biotic crisis near the Frasnian–Famennian boundary. Acta Palaeontologica Polonica. 2017;62:97–119. doi: 10.4202/app.00265.2016.

18. Eastman CR. On upper Devonian fish remains from Colorado. American Journal of Science. 1904;4 (18)(106):253–60. doi: 10.2475/ajs.s4-18.106.253.

19. Hoffman G. Über das Ruderorgan der Asterolepiden. Palaeontographica. 1911;57:286–312.

20. Woodward AS. Fish-remains from the Upper Old Red Sandstone of Granite Harbour, Antarctica. British Antarctica ("Terra Nova") expedition, 1910 Natural history report Geology. 1921;1(2):51–62.

21. Bryant WL. A new species of *Bothriolepis* from the Upper Devonian of Canada. Bulletin of the Buffalo Society of Natural Sciences. 1924;13:54–5.

22. Jaekel O. Der Kopf der Wirbeltiere. Ergebnisse der Anatomie und Entwickelungsgeschichte (III Abteil der Zeitschrift die gesamte Anatomie). 1927;27:815–974.

23. Hills ES. The geology and paleontography of the Cathedral Range and Blue Hills in northwestern Gippsland. Proceedings of the Royal Society of Victoria. 1929;41(N.S.):176–201.

24. Stensiö E. Arthrodires. In: Piveteau J, editor. Traité de Paléontologie. 4. Paris: Masson; 1969. p. 71–693.

25. Young GC, Gorter JD. A new fish fauna of Middle Devonian age from the Taemas/Wee Jasper region of New South Wales. Bureau of Mineral Resources, Geology and Geophysics Bulletin. 1981;209:83–147.

26. Leriche M. Les poissons famenniens de la Belgique - Les faciès du Famennien dans la région gallo-belge - Les relations entre les formations marines et les formations continentales du Dévonien supérieur sur la bordure méridionale du Continent Nord-Atlantique. Mémoire de la Classe des Sciences de l'Académie Royale de Belgique. 1931;4(10):1–72.

27. Stensiö E. Upper Devonian vertebrates from East Greenland. Collected by the Danish Greenland Expeditions in 1929 and 1930. Meddelelser om Grønland. 1931;86(1):1–212.

28. Gross W. Die Fische des baltischen Devons. Palaeontographica Abteilung A. 1933;69:1–74.

29. Sohn IG. *Bothriolepis stensiöi*, a new Devonian placoderm from Gaspé, Canada. Journal of Paleontology. 1938;12(1):111–3.

30. Robertson GM. Regarding *Bothriolepis stensiöi* Sohn. Journal of Palaeontology. 1938;12(3):299–300.

31. Obruchev DV. *Bothriolepis turanica* n. sp. from Western Tian-Shan. Doklady Akademii Nauk, s s. 1939;23:115–6.

32. Chi YS. On the discovery of *Bothriolepis* in the Devonian of central Hunan. Bulletin of the Geological Society of China. 1940;20(1):57–72. doi: 10.1111/j.1755-6724.1940.mp20001007.x.

33. Obruchev DV. Materials on Devonian Fishes of the USSR. Trudy Paleontologicheskogo Instituta Akademii︠a︡ Nauk SSSR. 1941;8:1–48.

34. Gross W. Die Bothriolepis-Arten der Cellulosa-Mergel Lettlands. Kungliga Svenska Vetenskaps-akademiens Handlingar. 1941;19(5):1–79.

35. Stensiö E. On the Placodermi of the Upper Devonian of East Greenland. II. Antiarchi. Sub-family Bothriolepinae. With an attempt at a revision of the previously described species of the family. Palaeozoologica Groenlandica. 1948;2:5–622.

36. Gross W. Die Fischfaunen des baltischen Devons und ihre biostratigraphische Bedeutung. Korrespondenz-blatt des Naturforscher-Vereins zu Riga. 1942;64:373–436.

37. Lukševičs E. Bothriolepid antiarchs (Vertebrata, Placodermi) from the Devonian of the north-western part of the East European Platform. Geodiversitas. 2001;23(4):489–609.

38. Denison RH. Late Devonian fresh-water fishes from the Western United States. Fieldiana: Geology. 1951;11(5):221–61.

39. Obrucheva OP. Upper Devonian Fishes of Central Kazakhstan. Soviet Geology. 1955;45:84–99.

40. Malinovskaya S. Devonian bothriolepids (Placodermi) from Central Kazakhstan. Byuleten’ Moskovskogo obschestva estestvoispytateley, geologiya. 1988;63(5):56–70.

41. Sergienko AA. A new species, *Bothriolepis extensa* sp. n., from the Tuba Formation of the Minusinsk Depressions. Tr Sib NauchIssled Inst Geol Geofiz Miner Syr ((Data on Paleontology and Stratigraphy of Western Siberia). 1961;15:139–40.

42. Panteleyev NV, Moloshnikov S. *Tubalepis* gen. nov. (Placodermi, Antiarchi) from the Upper Devonian of the Minusa Depression. Paleontological Journal. 2003;37:413–6.

43. Liu Y-H. A new species of *Bothriolepis* from Yunnan. Vertebrata PalAsiatica. 1962;6(1):80–7.

44. Chang K-J. A new species of *Bothriolepis* from Kwangtung. Vertebrata PalAsiatica. 1963;7(4):342–51.

45. P'an K. Some Devonian and Carboniferous fishes from South China. Acta Palaeontologica Sinica. 1964;12:139–68.

46. Chang K-J. New antiarchs from the Middle Devonian of Yunnan. Vertebrata PalAsiatica. 1965;9(pan1):1–14.

47. Miles RS. The Old Red Sandstone antiarchs of Scotland: Family Bothriolepididae. London: Palaeontographical Society; 1968 1968. 130 p.

48. Lyarskaja L, Savvaitova L. Structure and fossil fish fauna of Ketleri Formation of Latvia. In: Sorokin VS, editor. Rgional’naya geologiya Pribaltitki [= Regional Geology of Baltic]. Riga: Zinātne; 1974. p. 90–106.

49. Pan J, Wang S-T, Liu SY, Gu QC, Hang J. Discovery of Devonian *Bothriolepis* and *Remigolepis* in Ningxia. Acta Geologica Sinica. 1980;54:176–85.

50. Young GC. Antiarchs (placoderm fishes) from the Devonian Aztec Siltstone, Southern Victoria Land, Antarctica. Palaeontographica (A). 1988;202:1–125.

51. Weems RE, Beem KA, Miller TA. A new species of *Bothriolepis* (Placodermi: Bothriolepidae) from the Upper Devonian of Virginia (USA). Proceedings of the Biological Society of Washington. 1981;94(3-4):984–1004.

52. Weems RE. *Bothriolepis virginiensis*, a valid species of placoderm fish separable from *Bothriolepis nitida*. Journal of Vertebrate Paleontology. 2004;24(1):245–50.

53. Long JA. New bothriolepid fish from the Late Devonian of Victoria, Australia. Palaeontology. 1983;26(2):295–320.

54. Long JA, Burrett CF, Ngan PK, Janvier P. A new bothriolepid antiarch (Pisces, Placodermi) from the Devonian of Dô Son peninsula, northern Vietnam. Alcheringa: An Australasian Journal of Palaeontology. 1990;14(3):181–94. doi: 10.1080/03115519008619054.

55. Obrucheva HD, editor New bothriolepid species from deposits of the Zadonskian Regional Stage of the Central Devonian Field. Problemy sovremennoj paleoihtiologii (Materialy konferencii,posvâŝennoj D V Obručevu); 1983; Moscow: Nauka.

56. Moloshnikov SV. Devonian antiarchs (Pisces, Antiarchi) from central and southern European Russia. Palaeontological Journal. 2008;42(7):691–773.

57. Lyarskaja L. A new *Bothriolepis* (Antiarchi) from the Upper Devonian of Baltic states. In: Brangulis A, editor. Biofacii i fauna siluriyskogo i devonskikh basseynov Pribaltiki [=Biofacies and Fauna of Silurian and Devonian Basins of Baltics]. Riga: Zinātne; 1986. p. 123–30.

58. Lukševičs E. A new placoderm fish (Antiarchi) from Tervete Formation of Latvia. In: Brangulis A, editor. Biofacii i fauna siluriyskogo i devonskikh basseynov Pribaltiki [=Biofacies and Fauna of Silurian and Devonian Basins of Baltics]. Riga: Zinātne; 1986. p. 131–7.

59. Long JA, Werdelin L. A new late Devonian bothriolepid (Placodermi, Antiarcha) from Victoria, with descriptions of other species from the state. Alcheringa. 1986;10(4):355–99.

60. Long JA, Anderson ME, Gess R, Hiller N. New placoderm fishes from the Late Devonian of South Africa. Journal of Vertebrate Paleontology. 1997;17(2):253–68.

61. Johanson Z. New antiarchs (Placodermi) from the Hunter Siltstone (Famennian) near Grenfell, NSW. Alcheringa. 1997;21(3):191–217.

62. Johanson Z. The Upper Devonian fish *Bothriolepis* (Placodermi: Antiarchi) from near Canowindra, New South Wales, Australia. Records of the Australian Museum. 1998;50(3):315–48.

63. Johanson Z, Young GC. New Bothriolepis (Antiarchi: Placodermi) from the Braidwood region, New South Wales, Australia (Frasnian). Records of the Western Australian Museum Supplement. 1999;57:55–75.

64. Lukševičs E, Sorokin VS. A new species of armored fishes of the genus *Bothriolepis* (Placodermi) from the Upper Devonian of North Timan. Paleontological Journal. 1999;33(4):413–9.

65. Moloshnikov SV, Linkevich VV. Late Devonian bothriolepidids (Placodermi, Antiarchi) of the Tver Oblast. Paleontological Journal. 2020;54(2):157–65. doi: 10.1134/S0031030120020100.

66. Young GC, Moody JM. A Middle-Late Devonian fish fauna from the Sierra de Perijá. Mitteilungen des Museums für Naturkunde Berlin Geowissenschaftliche Reihe. 2002;5:155–206.

67. Moloshnikov SV. A new species of bothriolepidid antiarch (Pisces, Placodermi) from the Zadonskian Regional Stage (Upper Devonian) of the Central Devonian Field. Paleontological Journal (Paleontologicheskii zhurnal). 2003;37:69–72.

68. Szrek P. The first articulated antiarch (Vertebrata, Placodermi) from the Upper Devonian of the Holy Cross Mountains (central Poland). Acta Geologica Polonica. 2004;54(3):401–6.

69. Moloshnikov SV. New data on late Devonian bothriolepidid Placoderms (Pisces, Antiarchi) from Tuva. Paleontological Journal. 2009;43(5):558. doi: 10.1134/S0031030109050128.

70. Moloshnikov SV. A new find of the placoderm genus *Bothriolepis* Eichwald in the Upper Devonian of Uzbekistan. Paleontological Journal. 2010;44:79–83. doi: 10.1134/S0031030110010107.

71. Downs JP, Daeschler EB, Garcia VE, Shubin NH. A new large-bodied species of *Bothriolepis* (Antiarchi) from the Upper Devonian of Ellesmere Island, Nunavut, Canada. Journal of Vertebrate Paleontology. 2016;36(6).

72. Scotese C, cartographer Atlas of Devonian Paleogeographic Maps, PALEOMAP Atlas for ArcGIS, volume 4, The Late Paleozoic, Maps 65-72, Mollweide Projection, PALEOMAP Project, Evanston, IL2014.

73. Scotese C. Scotese, C.R., 2014. Atlas of Neogene Paleogeographic Maps (Mollweide Projection), Maps 1-7, Volume 1, The Cenozoic, PALEOMAP Atlas for ArcGIS, PALEOMAP Project, Evanston, IL2014.

74. Scotese C, Wright N. PALEOMAP Paleodigital Elevation Models (PaleoDEMS) for the Phanerozoic PALEOMAP Project, <https://www.earthbyte.org/paleodem-resourcescotese->. 2018.

75. Scotese CR. Paleogeographic Atlas, PALEOMAP Progress Report 90-0497. Arlington (Texas): University of Texas Press; 1997. 1–45 p.

76. Zhu M. Catalogue of Devonian vertebrates in China, with notes on bio-events. In: Blieck A, Turner S, editors. Palaeozoic Vertebrate Biochronology and Global Marine/Non-Marine Correlation Final Report of IGCP 328 (1991-1996). 223. Frankfurt: Courier Forschungsinstitut Senckenberg; 2000. p. 373–90.

77. Sallan LC, Coates MI. End-Devonian extinction and a bottleneck in the early evolution of modern jawed vertebrates. Proceedings of the National Academy of Sciences of the United States of America. 2010;107(22):10131–5.

78. Rogers DA. Devonian correlations, environments and tectonics across the Great Glen Fault (Unpublished PhD Thesis): University of Cambridge; 1987.

79. Becker RT, Gradstein FM, Hammer O. The Devonian Period. In: Gradstein FM, Ogg JG, Schmitz M, Ogg G, editors. The Geological Time Scale. Amsterdam: Elsevier; 2012. p. 559–601.

80. Moloshnikov SV. Middle-Late Devonian placoderms (Pisces: Antiarchi) from Central and Northern Asia. Paleontological Journal. 2012;46(10):1097–196.

81. Moloshnikov SV. Bothriolepiform antiarchs (Pisces, Placodermi) from the Devonian of Central Kazakhstan. Paleontological Journal. 2011;45(3):291–304. doi: 10.1134/S0031030111030099.

82. Lukševičs E, Zupiņš I. Sedimentology, fauna, and taphonomy of the Pavari site, Late Devonian of Latvia. Acta Universitatis Latviensis. 2004;(679):99–119.

83. Blom H, Clack JA, Ahlberg PE, Friedman M. Devonian vertebrates from East Greenland: a review of faunal composition and distribution. Geodiversitas. 2007;29(1):119–41.

84. Browne MAE, Smith RA, Aitken AM. Stratigraphical frmework for the Devonian (Old Red Sandstone) rocks of Scotland, south of a line from Fort William to Aberdeen Keyworth, Notthingham: British Geological Survey; 2002. 67 p.

85. Moloshnikov SV. Middle Devonian bothriolepiform antiarchs (Pisces, Placodermi) from central Kazakhstan and their implication for the antiarch system and phylogeny. Paleontological Journal. 2010;44:195–208. doi: 10.1134/S0031030110020127.

86. Olive S. Devonian antiarch placoderms from Belgium revisited. Acta Palaeontologica Polonica. 2015. doi: <http://dx.doi.org/10.4202/app.00015.2013>.

87. Trewin NH, editor. The Geology of Scotland. 4^th^ edition ed. London: The Geological Society; 2002.

88. Janvier P, Villaroel C. Devonian vertebrates from Colombia. Palaeontology. 2000;43:729–63.

89. Stack J, Sallan L. An examination of the Devonian fishes of Michigan. PeerJ. 2018;6:e5636–e. doi: 10.7717/peerj.5636. PubMed PMID: 30258725.

90. Marsden MA. Upper Devonian Carboniferous. In Geology of Victoria, Douglas, J. G. & Fergusson J. A. eds. Special Publication, Geological Society of Australia. 1976;5:77–124.

91. Lu L-W, Tan K, Wang X. A new Antiarchi (placoderm fishes) from Devonian strata of Dushan, Guizhou Province. Acta Geoscientia Sinica. 2017;38(2):144–8.

92. Pan Z-H, Zhu M, Zhu YA, Jia LT. A new antiarch placoderm from the Emsian (Early Devonian) of Wuding, Yunnan, China. Alcheringa: An Australasian Journal of Palaeontology. 2017;42(1):10–21. doi: 10.1080/03115518.2017.1338357.

93. Moloshnikov SV. Crested antiarch *Bothriolepis zadonica* H.D. Obrucheva from the Lower Famennian of Centrak European Russia. Acta Geologica Polonica. 2004;49(1):135–46.

94. Wang Y-J, Zhu M. Redescription of *Phymolepis cuifengshanensis* (Antiarcha: Yunnanolepididae) using high-resolution computed tomography and new insights into anatomical details of the endocranium in antiarchs. PeerJ. 2018;6:e4808. doi: 10.7717/peerj.4808.
