# Supplementary material for: The Bothriolepis (Placodermi, Antiarcha) material from the Valentia Slate Formation of the Iveragh Peninsula (middle Givetian, Ireland): Morphology, evolutionary and systematic considerations, phylogenetic and palaeogeographic implications
Source: PLoS One. 2023 Feb 23;18(2):e0280208. doi: 10.1371/journal.pone.0280208 (PMC9949654; doi:10.1371/journal.pone.0280208)
Supplement: Attachment — Submitted filename: rebuttal letter - answer to revewiers_20221215.pdf [file pone.0280208.s009.pdf]

Uppsala, 15th December 2022-

Dear Colleague,

It is with great pleasure that I hereby submit our revised version of the article entitled “The *Bothriolepis* (Placodermi, Antiarcha) material from the Valentia Slate Formation of the Iveragh Peninsula (middle Givetian, Ireland): morphology, evolutionary and systematic considerations, phylogenetic and palaeogeographic implications”. I have taken into account all of your comments as well as those of the reviewers. Details are listed below:

1. I have reformatted the structure of the text so that it fits PLOS ONE’s style requirements. Notably, there are only 3 levels of headings (as per our previous discussions). The figures were transformed to the following format: TIFF, single-layered, LZW compression, 300dpi, less than 10 Mo, and checked with PACE.
2. The new taxon and the submitted article have now a GUID and LSID at Zoobank. This information was added in the text at the required locations, together with the texts you mentioned (for which the subsection “Nomenclatural acts” was created in “Methods”).
3. Funding information was removed from the Acknowledgements section. Please update on our behalf the Funding Statement section in the online submission form with the following text. “This work is funded by a Wallenberg Scholarship from the Knut and Alice Wallenberg Foundation and an ERC Advanced Grant (ERC-2020-ADG 101019613 "Tetrapod Origin"), both awarded to P.E.A. Except for K.H. who personally funded the physical preparation of the material in Switzerland, the funders had no role in study design, data collection and analysis, decision to publish, or preparation of the manuscript.”
4. As corresponding author, I belong to Uppsala University, and my profile has been corrected since original submission. Billing details are Uppsala Universitet, Invoices, Box 148, 751 04 Uppsala. Reference code is 148A03.
5. The repository for data is currently under progress at DORIS SND (Swedish National Data Service). The provisional address is: https://snd.gu.se/en/catalogue/study/preview/01477b73-c184-4f67-8e62-cb5f97a42c30#dataset
6. 6.1. As for Fig. 1 of our article, it consists of a geological map extremely modified from the original one published by Russell in 1978 and could be considered as a new creation (who is also co-authoring the present article). I therefore propose to remove the mention “modified after Russel 1978”. I let you decide whether this is opportune; the original geological map is uploaded as “OTHER” as for the consideration of adequate editors (as per email sent 2022-11-28).
   6.2. For the Figures related to Palaeogeography in the main article as well as the Supplementary Information, I obtained the authorization from C. Scotese. A pdf of his email is part of the submitted files, and all captions were modified accordingly (Figs 15-17 and S3-8 Figs).
7. Supplementary Information captions are added at the end of the main manuscript. Tables S5-8 that are included in the Supplementary Text are now cited as “TABLE A-D in S1 Text”. Thus former S9,10 Tables are renamed S 5,6 Tables.
8. To our knowledge, the reference list is correct and up to date.
9. Answers to reviewer 1:

9.1. About some formal Latin names that were not italicised: This is related to WORD automated format for titles. In agreement with the Editor (email 2022-11-28), these mistakes will be managed by the editor himself from the original WORD document.
9.2. “Gondwana” vs. “Gondwanan” occurrences are corrected.
9.3. Fig. 4A,C were modified to appear lighter (as per reviewer and editor’s wish – email 2022-11-28).
9.4. The references mentioned by the reviewer as missing were actually cited in the text and listed in the References section.

Additionally, some new specimen numbers were added in the main text and Supplementary text (replacing the former temporary ones: also implemented in all captions). Mention of the regression line are removed from the text (p. 73) and from the S2 Figure (it had no biological significance because it related only to the publication dates of new taxa).

Some minor modifications were made in the main text and S1 text (e.g. captions) and marked up. Mentions of Supplementary Information referring to the word file were replaced with “”S1 Text”.

We thank you very much for the time spent at considering our research article.

Sincerely,

Vincent Dupret.
